# Successful prediction of LC8 binding to intrinsically disordered proteins sheds light on AlphaFold’s black box

**DOI:** 10.3389/fmolb.2025.1531793

**Published:** 2025-04-23

**Authors:** Douglas R. Walker, Gretchen Fujimura, Juan M. Vanegas, Elisar J. Barbar

**Affiliations:** Department of Biochemistry and Biophysics, Oregon State University, Corvallis, OR, United States

**Keywords:** AlphaFold 2, intrinsic disorder, LC8 dynein light chain, hub protein, explain AI, protein binding, binding predictions

## Abstract

**Introduction:**

LC8 is a hub protein involved in many processes from tumor suppression and cell cycle regulation to neurotransmission and viral infection. Despite recent progress, prediction of binding sites for LC8 is plagued by motif variability and a multitude of weakly binding motifs, especially when binding depends on multivalency. Our binding site prediction algorithm, LC8Pred has proven useful for uncovering new LC8 binders, but is insufficient for finding all LC8 binding sites.

**Methods:**

To address this, we probed the ability of a general structure predictor, AlphaFold, to predict whether a given sequence binds to LC8. Certain combinations of in-built AlphaFold scores were extracted and distributions of scores of binders were compared to scores of nonbinders.

**Results:**

AlphaFold successfully places proteins at the correct interface of LC8. A set of threshold values of built-in AlphaFold scores enables differentiation between known binders and nonbinders with minimal false positive (8%) and acceptable false negative rates (20%). This cutoff, along with a more inclusive cutoff, was used to predict elusive LC8 binding sites in proteins known to bind LC8.

**Discussion:**

Correlations between binding affinities and AlphaFold scores provide insight into the black box and indicate that AlphaFold learned an inaccurate energy function that nevertheless is useful for making inferences and conclusions about physical systems. Binding sites predicted by this method can be prioritized for investigation by comparing to result by LC8Pred, local structure, and evolutionary conservation.

## Introduction

It is estimated that there are between 130,000 and 600,000 protein–protein interactions (PPIs) at work in human biology (Bonetta, 2010; Venkatesan et al., 2009), which make up the human interactome. From enzymatic activity to gene regulation and repair, cell signaling, transport, structure, and the cell cycle, proteins are the workhorses of the cell, and protein interactions are the means by which cellular functions are carried out. One particularly large class of PPIs is mediated by short linear motifs (SLiMs) (Dinkel et al., 2012). Similarly, it has been predicted that there are on the order of hundreds of thousands of SLiMs in the human proteome, which likely dominate the human interactome (Tompa et al., 2014). However, despite the predominance of these interactions, they remain extraordinarily understudied since they are difficult to detect, even more difficult to pinpoint, and thus problematic to characterize.

Hub proteins and their interactions represent a significant proportion of the interactome, given that the majority of proteins only interact with a few partners, but hubs interact with a large number of proteins through complex networks (Jeong et al., 2001; Jeong et al., 2000). Hub proteins include (1) multi-interface hubs, which bind to many different proteins simultaneously at different sites of the protein, and (2) single-interface hubs, which bind to many different partners or locations on partners, but all at the same binding site (or symmetric binding sites), allowing interaction with only one (or two) partners at a time (Han et al., 2004; Kim et al., 2008). Interestingly, protein disorder plays an important role in the binding of single-interface hubs to their partners, whether that be in the hub or the partner (Kim et al., 2008; Patil et al., 2010; Uversky, 2016).

SLiMs (also called eukaryotic linear motifs [ELMs] or molecular recognition features [MoRFs]) are common in intrinsically disordered regions (IDRs) (Uversky, 2016; Oldfield et al., 2005; Vandereyken et al., 2018) and often serve as vehicles for interactions between hubs and their partners (Vandereyken et al., 2018; O’Shea et al., 2017). When the hub is disordered and the partner is ordered, the hub will often contain a SLiM to facilitate recognition. The reverse scenario is also true, as seen in the hub protein LC8 (Benison et al., 2007; Barbar, 2008; Rapali et al., 2011), where the hub is ordered and binds disordered proteins at SLiMs. Hub proteins like LC8 have recently been further classified as linear motif-binding (LMB) hubs (Jespersen and Barbar, 2020), which also include 14-3-3, calmodulin, SH3 domains, and the serine/threonine-specific protein kinase B. This reclassification formalizes and highlights the importance of the link between dynamic hub proteins and linear motifs, thereby bridging questions and problems observed in each area of research and hopefully leading to further collaboration and answers.

The intersection between SLiMs and hub proteins offers a promising avenue for expanding our grasp of the extent of protein interactions: the ability of a protein to recognize a variable motif across many different partners suggests that algorithmic reasoning can recognize such motifs and thereby discover previously unknown interactions. This was the impetus behind our design of the algorithm LC8Pred (Jespersen et al., 2019). The prediction of binding between the hub protein LC8 and new partners has been useful in elucidating the function of these partners (Howe et al., 2022). However, an algorithm like LC8Pred can only be trained on information from previously identified binding partners and their identified SLiMs. LC8-binding partners for which no SLiM has been experimentally identified (Di Bartolomeo et al., 2010; Den Hollander and Kumar, 2006; Nakano et al., 2010) have been reported, and in some of these cases, LC8Pred cannot confidently predict a binding site (Rayala et al., 2005; Emi et al., 2005). For this reason, it is important to go beyond simple motif recognition and use our understanding of protein structure, folding, and interactions to predict whether, and where, a protein will bind to LC8 (or, by extension, to predict interactions between any two proteins).

LC8 (dynein light chain 8) is an 89-residue protein that forms a rotationally symmetric homodimer. Along the edge of the binding interface, parallel to the axis of symmetry, LC8 binds to its partner proteins. Binding two copies of a client, one on each side, allows LC8 to duplex its partners, which is the mechanism by which LC8 functions. Duplexing by LC8 serves a variety of purposes such as associating two strands to enable functions inaccessible to monomers, strengthening the association of two already associated strands to increase the potency of a function, scaffolding structural proteins through multivalent interactions, and facilitating heterogeneous complexation for concentration sensing and regulation. LC8 binds its partners at an 8–10-residue-long motif. The most strongly conserved position, known as the anchor, is often glutamine (Q) (although asparagine, methionine, serine, and even glycine have been observed) with flanking threonines (T). The binding motif extends 2 residues C-terminally and 5–7 residues N-terminally from the anchor. For the remaining positions, subtle patterns are present and have been discussed previously (Jespersen et al., 2019; Erdős et al., 2017).

Currently, the most efficient way to use our knowledge base of protein folding and interactions is through structure prediction algorithms like AlphaFold2 (Baek et al., 2021). With the advent of AlphaFold-Multimer (Evans et al., 2021), we could predict the structures of multimeric protein complexes. Independent improvement packages like ColabFold (Mirdita et al., 2022) have accelerated AlphaFold calculations, making it more useful as a high-throughput technique and accessible to all. AFsample has further improved the quality of structural models using different sampling schemes to generate thousands of models (Wallner, 2023). Depending on the system, AlphaFold can predict alternate conformational states (Stein and Mchaourab, 2022). AlphaFold and RoseTTAFold (Baek et al., 2021) have been used to explore interactomes and reveal new protein interactions (Humphreys et al., 2021; Lim et al., 2023). EvoBind, based on AlphaFold, was developed to design peptides that bind to protein interfaces (Bryant and Elofsson, 2022). Multiple reports have discussed AlphaFold’s ability to distinguish binding from non-binding peptides, to characterize non-canonical binders, and even to rank the affinities of peptide binders to proteins (Bryant and Elofsson, 2022; Chang and Perez, 2023; Ibrahim et al., 2023). AlphaFold can predict the location of a SLiM within a long disordered region of a protein with high accuracy (>80%) (Bret et al., 2024). Even more promising, it successfully predicts structures of PPIs involving IDRs with similarly high accuracy (>75%) (Omidi et al., 2024). This analysis studied a variety of IDR binding modes, which included homogeneous binding and dynamic fuzzy complexes. These reports are significant because AlphaFold is often considered inadequate for predicting disordered domains.

AlphaFold evaluates its own structure predictions with four metrics: predicted local distance difference test (pLDDT), predicted aligned error (PAE), predicted template modeling (pTM), and interface pTM (ipTM) scores. pLDDT reports on local confidence (Mariani et al., 2013; Magana Gomez and Kovalevskiy, 2024) and ranges from 0 to 100. PAE measures the confidence in the relative position of two residues. According to the EMBL-EBI AlphaFold training (Magana Gomez and Kovalevskiy, 2024), PAE is defined as “the expected positional error at residue X, measured in Ångströms (Å), if the predicted and actual structures were aligned at residue Y.” PAEs range from 0 to 31.5. pTM and ipTM are specific to AlphaFold-Multimer and are derived from template modeling (TM) scores (Magana Gomez and Kovalevskiy, 2024; Xu and Zhang, 2010), which measure the accuracy of the protein structure globally, without overweighting the local inaccuracies. ipTM in particular reports on the interface between the proteins being predicted. When AlphaFold-Multimer ranks its predictions for reporting, it calculates a confidence score that is a weighted average of the TMs, specifically, it is 80% ipTM and 20% pTM.

AlphaFold has learned an energy function through its training (Roney and Ovchinnikov, 2022; Herzberg and Moult, 2023; Ahdritz et al., 2024), so it may predict rare structures or even ones that do not resemble anything found in the PDB (Berman et al., 2000; Berman et al., 2003). Duignan proposed that while this learned energy function must share minima with the true protein energy landscape due to the high representation in AlphaFold’s training set of energy minimum structures, the learned energy function will deviate from the true landscape at higher energies (Duignan, 2024). Although reports illustrating AlphaFold’s ability to predict alternate conformations (Stein and Mchaourab, 2022) imply that the magnitude of these “higher energies” necessary to observe significant differences may be greater than intuition would suggest, this is likely due to the presence of NMR solution structures in the PDB, on which AlphaFold was trained.

Given this context, AlphaFold is a promising tool for complementing our predictions of LC8 interactions through its general knowledge of proteins. However, for applications such as this, it is advised and important to include examples of nonbinders (Lee et al., 2024). Suggested approaches include randomizing protein sequences or mutating the SLiM to polyalanine. Similarly, concerns have been raised about the multiple different versions of AlphaFold and metrics used to distinguish its ability to accurately assess binding (Lee et al., 2024). In this work, we investigate AlphaFold’s ability to distinguish proteins that bind LC8 from known nonbinders. We establish criteria to distinguish binders from nonbinders based on AlphaFold’s pLDDT, PAE, pTM, and ipTM scores. We explore the potential for AlphaFold scores to correlate with binding affinities as might be expected if AlphaFold has learned something about protein folding energetics. Finally, we apply these lessons to predict new LC8 binding sites in a selection of proteins that (1) are known to, but not where, bind LC8, (2) are not predicted by LC8Pred, or (3) for which LC8Pred predicts that more sites may exist than have been experimentally found. LC8Pred and AlphaFold predictions are compared to guide hypothesis generation and experimental design to find new LC8 binding sites.

## Methods

### Identification of proteins for analysis

The information on LC8 binders was collected from the literature, which included our database LC8hub (Jespersen et al., 2019), previously assembled libraries, and other reports on binders. The selection of nonbinders was determined from multiple sources, such as (1) mutants of binders in which the anchoring triad is mutated to AAA, (2) the selection of nonbinders reported in Jespersen et al. (2019), and (3) regions of well-studied, highly characterized LC8-binding proteins that are not in the binding region. The proteins chosen for investigation and detection of new LC8-binding sites were selected based on one or more of several features of interest: (1) LC8Pred predicts more binding sites than have been reported, (2) LC8hub contains no binding sequence for a known LC8 binder, (3) LC8Pred does not provide good predictions for the experimentally characterized binding sites, and (4) proteins that also contain binding sites for other proteins, suggesting a mechanism of partnership between LC8 and the other binder. This refers to Supplementary Presentation 1 as named in the production forum.

To parse long protein sequences into smaller sections for more precise identification of LC8-binding sites and to obtain lower, tenable scores for analysis, a Python script was employed that reads a full protein sequence, extracts a subsequence, adds the necessary LC8 sequences, and generates a new FASTA file for prediction.

### LC8-binding site modeling and assessment

All sequences of interest were run using ColabFold, locally installed on a server hosted by the Vanegas laboratory, with all the necessary contexts: one or two copies of the protein, wild-type or mutated anchoring site, and an LC8 dimer. The majority of predictions were prompted to return 25 structures; however, a few early runs returned five structures and were still included in the analysis, and some later runs were prompted to return 100 structures to better investigate the correlation between successful prediction rates and binding affinities. The results were downloaded locally and processed with a set of three Python scripts to extract the AlphaFold scores from each prediction, which were used throughout the rest of the analysis.

### Aggregating and analyzing the full set of processed predictions

After processing each prediction and assembling the results, additional Python scripts were used to aggregate the data into a unified dataset. These scripts assigned a binding status—binder, nonbinder, or unknown binder—to each prediction, classified each identified bound sequence into a known binder as on- or off-target, linked results from 2-client runs to the appropriate results from 1-client runs, plotted results, assessed false-positive and false-negative rates for set score thresholds, and trained an optimal classifier for differentiating binders from nonbinders, along with generating the accompanying learning curve. In training the classifier, a genetic algorithm was used to optimize the AUROC. This process was used to construct a learning curve where different amounts of the data were randomly separated for training and testing. The learning curve plots the computed accuracy of the most accurate threshold along the ROC. The thresholds used were then taken from one of the runs trained on 90% of the data, in particular, the run that achieved the highest AUROC, regardless of how the test data performed.

### Affinity analysis by linear regression and mutual information

A set of LC8-binding motifs (8-AA-long) with known binding affinities was collected from the literature. A script was written to scan through the AlphaFold predictions, searching for sequence matches among the known binders. When sequence matches were found, all pertinent scores we have described were extracted and included in new .csv files, which were further analyzed in Excel using the LINEST function to calculate R^2^ values for the relationships between the scoring parameters and binding affinities. For the calculation of the adjusted mutual information (AMI), another script was written because Excel does not support the evaluation of mutual information. This script took the .csv file output from affinity_analysis.py and used the sklearn package (Pedregosa et al., 2011) to calculate mutual information. This script binned the values of both the affinities and the scores in question. For each scoring parameter, identical bins were used for each data subset to enable direct comparison between dataset evaluations. MI quantifies how much knowledge of one variable informs an associated variable. It represents the overlap between the individual Shannon entropies of two compared variables. Adjusted mutual information corrects for the effect of agreement that comes from randomness. An expected MI is calculated under the assumption that two distributions are randomly clustered. This expected MI is then subtracted from the calculated MI, and the resulting difference is the numerator. The denominator consists of the difference between the greater entropy of the two datasets and the expected mutual information, forming the quotient used to calculate the AMI. This quotient ensures that the AMI falls in the range of 0–1. The calculations of the AMI are performed using the sklearn Python package.

### Bayesian Inference

Bayesian probability interprets probabilities as the degree of rational belief in a statement rather than as a propensity or relative frequency of occurrence. Bayesian inference uses this principle and formalizes a method for determining the probability of a given hypothesis being true based on a given piece of evidence, according to Equation 1:

$$P\left(H_{i}|E\right)=\frac{P\left(\left.E\right|H_{i}\right)*P\left(H_{i}\right)}{P\left(E\right)},$$ (1)

where H_i_ indicates some hypothesis, E indicates evidence, and | is the symbol for given. Thus, P(H|E) is the probability that hypothesis H is true, given that evidence E is true. It should be noted that when comparing two different hypotheses [P(H_1_|E)/P(H_2_|E)], the denominator cancels out because it is always the same, regardless of which hypothesis is being considered. P(E) has no dependence on H. P(H) is called the prior probability, i.e., the probability that the hypothesis is true before considering any evidence. In qualitative assessments, this term is often set as equal for all hypotheses, which means that it also cancels out in a comparative ratio. This is shown in Equation 2, and illustrates a key reason why handling these values with logarithms is advantageous.

$$\begin{array}{ll} & \log\left(\frac{P\left(H_{1}\left|E\right.\right)}{P\left(H_{2}\left|E\right.\right)}\right)=\log\left(P\left(H_{1}|E\right)\right)-\log\left(P\left(H_{2}|E\right)\right)=\log\left(P\left(E|H_{1}\right)\right)\\ & \;-\log\left(P\left(E|H_{2}\right)\right)=\log\left(\frac{P\left(\left.E\right|H_{1}\right)}{P\left(\left.E\right|H_{2}\right)}\right). \end{array}$$ (2)

As shown in Equation 2, this approach yields multiple benefits. (1) Absolute probabilities are not needed—only a difference between two log-likelihoods is important. (2) As mentioned in the main text, this is how human perception scales, making this an intuitive evaluation. (3) Combining multiple pieces of evidence is as simple as adding the log-likelihood differences for both pieces of information. (4) The problem of comparing more than two hypotheses increases linearly, rather than quadratically. If H_1_ has already been compared to H_2_ and H_3_ separately, H_2_ can be compared to H_3_ through simple subtraction of the H_1_/H_2_ and H_1_/H_3_ comparisons.

### Predicting new binding sites and their affinities

Another script searches through all the assembled AlphaFold runs, looking for predictions of sequences with an unknown LC8-binding status. The combination of all AlphaFold scores assessed was compared to the cut-off thresholds that were established in this work. Structures that achieve the cut-off threshold are predicted to bind LC8 with the false-positive and false-negative rates associated with each threshold set of values. Because all AlphaFold runs were completed before we performed our affinity analysis of different subsets, all of these predictions were run with 16 amino-acid-long sequences. The anchor motif was determined by finding the residue in the client sequence that is closest to G63 in one of the LC8 protomers in the .pdb structure file and setting that residue as position 0. Affinity predictions are rudimentary because the prediction is meant only to be taken as approximate due to the nature of the relationships and the relatively weak or moderate correlations. Equations 3 and 4 show the affinity calculations for sites containing a TQT anchor and those containing any other anchor:

$${Affinity}_{TQT}=\frac{1client\;Dimer\;PAE-0.89}{0.0035},$$ (3)

$${Affinity}_{!TQT}=\frac{Average\;1client\;Avg\;P\to L\;PAE-4.45}{0.135},$$ (4)

where “Average 1client Avg P- > L PAE” takes the average of the “1client Avg P- > L PAE” across all 25 structures in the given AlphaFold prediction. These relationships were used because they represent the strongest linear relations calculated for each subset of predictions on 16 amino-acid sequences. It should be noted that these are linear relations and so inevitably result in a negative affinity prediction for some binding sites. In such cases, we set the predicted affinity to 1 µM. To emphasize the degree of uncertainty in these predictions, it is worth mentioning that the R^2^ value for the TQT set was 0.609, and it was 0.292 for the subset with different anchors. These predictions illustrate how important it is that more LC8 binding sites be characterized so that additional homogeneous sub-datasets can be evaluated.

## Results

### Benchmarking AlphaFold predictions of LC8 dimers

**FIGURE 1:**
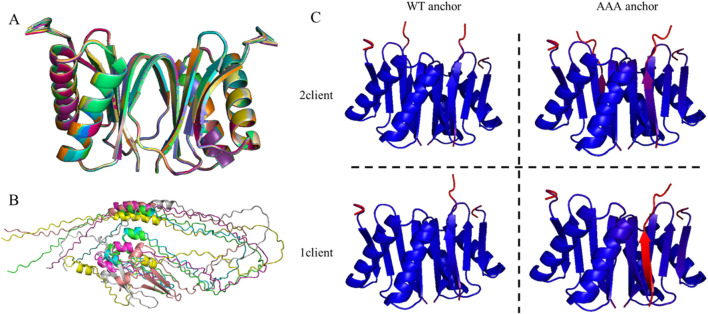
AlphaFold predicted structures. **(A)** Overlay of the 25 structures produced by an AlphaFold prediction of the LC8 dimer illustrating prediction robustness. **(B)** Overlay of six AlphaFold structure predictions of the dASCIZ LC8-binding domain, illustrating variability in the prediction. **(C)** Four predictions of LC8 with dASCIZ QT3, with WT or AAA mutant linker and predicted with 1 or 2 copies of the client. Structures are colored by pLDDT on a gradient from red to blue for values from 70 to 100. As observed, the linker mutation disfavors the nonbinding sequence, but the half-bound (1-client) structure is a better discriminator.

When assessing predictions of proteins bound to a homodimer, it is prudent to first assess the homodimer alone. When predicting the LC8 homodimer, all 25 resulting structures were found to align perfectly (Figure 1A) and consistent with the known structure of LC8, even down to the partially folded state of β3. This consistency lends confidence to further predictions involving partner proteins and suggests that low-confidence predictions are due to the client rather than a random, inaccurate LC8 prediction. Confidence scores range from 0.89 to 0.91, which signifies high-confidence predictions. Average PAE values for contacting residues in the interface (excluding the C-termini) range from 0.94 to 1.08 Å, reflecting a highly confident predicted distance between the protomers. This average PAE metric was tracked throughout the analysis to ensure that the quality of the LC8 dimer prediction remained robust. In contrast, the LC8-binding domain of ASCIZ would serve as a poor benchmark for this type of analysis. The variability in the predictions of the multivalent LC8 partner ASCIZ (Figure 1B) would make multiple comparisons difficult, if not impossible.

### Dataset characteristics

Having established confidence in AlphaFold’s ability to predict LC8, we then assessed its ability to distinguish between binders and nonbinders. When LC8 binds a partner, the intrinsically disordered partner folds as a beta-strand and binds to β3 of LC8, stabilizing β3 in the process. We co-predicted LC8 with many client sequences of various lengths and analyzed the quality of these results using AlphaFold’s self-reported scores. A series of scripts located the binding region of the client and extracted appropriate scores, such as (1) the confidence score, (2) the average pLDDT value along the length of the bound region, (3) the average PAE of β3 relative to the bound client (hereafter called LtoP for LC8 to peptide), (4) the average PAE of the bound client to β3 (PtoL), and (5) the average PAE of the LC8 dimer interface (a more in-depth discussion is in the *Methods* section). The first two PAE values appear, by definition, to be equal, but in practice, they are not and require separate evaluation. To explain anthropomorphically, one of the PAEs represents how comfortable LC8 β3 is with the environment, while the other is for the client peptide. The LtoP PAE will always be better than the PtoL PAE due to the structure of LC8 compared with the intrinsic disorder of the client peptide.

In brief, it is important to consider the entirety of a given interaction to generate a fair assessment of a method used to predict said interaction. LC8 binding is cooperative, with the first binding event incurring an entropic penalty due to the rigidification of LC8, resulting in the second binding event being more stable than the first (Estelle et al., 2023). This ensures that very little LC8 exists in a half-bound state and that LC8 binds symmetrically: different clients are not bridged (Reardon et al., 2020), and LC8 does not bind off-register when binding multivalent clients (Reardon et al., 2020). For further context, some multivalent clients bind multiple LC8 dimers cooperatively, like Nup159 (Nyarko et al., 2013), but some bind LC8 both cooperatively and anti-cooperatively at different sites (Reardon et al., 2020; Clark et al., 2018; Walker et al., 2023) like ASCIZ, depending on the function of LC8 in the interaction.

Since LC8 binding is cooperative, there may be clients that are stable when doubly bound but cannot surpass the energy barrier of the singly bound intermediate to reach the doubly bound state. As such, it is important to consider whether AlphaFold can distinguish between binders and nonbinders in either of these two states; a prediction is considered successful when AlphaFold correctly predicts a nonbinding sequence to not bind when only one copy is present, even if it incorrectly predicts binding with two copies. Therefore, AlphaFold predictions were carried out for LC8 with two copies of the partner protein (2-client) and with one copy (1-client) (Figure 1C). Furthermore, as we did when developing LC8Pred (Jespersen et al., 2019) and as cautioned by others working with AlphaFold (Lee et al., 2024), we tested AlphaFold’s ability to predict known nonbinding sequences. These were sourced from those used in developing LC8Pred, AAA mutations of the anchoring triad of a binder (illustrated in Figure 1C), non-binding regions of well-characterized binders, and nonbinding proteins.

**FIGURE 2:**
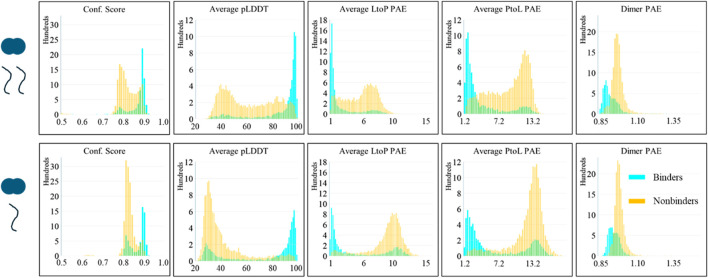
AlphaFold scores of the predictions of LC8 modeled with binders and nonbinders show distinct score differences between the average binder and the average nonbinder. (Top) Scores of the 2-client predictions. (Bottom) Scores of 1-client predictions. (left to right) Confidence score, average pLDDT of the binding site and binding sequence, average LtoP PAE, average PtoL PAE, and the average dimer interface PAE. Binding status is color-coded: binders are shown in cyan, and nonbinders are shown in orange.

Approximately 7,500 different AlphaFold predictions were run, with the majority of them producing 25 structures (a few only produced five structures). These predictions included 641 nonbinding sequences (of which 382 are AAA mutants) and 333 binding sequences with an approximately even split between 2-client- and 1-client-type predictions. The sequence lengths of the client in these predictions vary from 10 to 71 amino acids. The remaining >6,000 predictions were run with the intent of using AlphaFold to locate novel binding sites in known LC8-binding proteins: 24 proteins were parsed into 16-amino acid-long sections with an 8-amino acid overlap and predicted with LC8 in both 1-client and 2-client runs. As mentioned above, the average PAE of the LC8 dimer interface was tracked, and in all cases is consistent with the highly confident LC8-dimer-alone prediction, indicating a robust LC8 dimer is the foundation for each prediction, as shown in Figure 2.

### Prediction of LC8 binding

The assembled AlphaFold predictions and their scores were analyzed as described. An initial examination of the predicted structures shows LC8 binding in nearly all cases and might lead to us concluding that AlphaFold has no ability to predict nonbinders but will always just “put proteins together” as it does regularly. However, structures are the superficial part of the prediction, and AlphaFold’s confidence in its own predictions must also be considered. The distributions of the scores from the predictions are shown in Figure 2. Interestingly, distinct patterns emerge between the two client types analyzed; 2-client runs show good scores for binders, but a significant portion of nonbinders also yield confident scores despite being nonbinders, and 1-client runs achieve the desired deficiency of nonbinders in the confident region, but binders display a bimodal population in which a significant number of binders have scores more typical of nonbinders. Supplementary Figure S1 shows the corner pair plot of all scores, further illustrating the separation based on the binding status. This shows that AlphaFold can provide some level of discrimination between binders and nonbinders.

**FIGURE 3:**
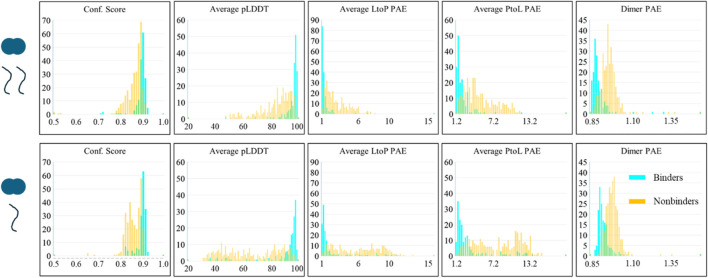
AlphaFold scores of the best predictions of LC8 modeled with binders and nonbinders show the predictability between binders and nonbinders. Panels and colors are identical to Figure 2.

It is important to note that the analysis in the previous section includes the results of all the structures in each prediction. It is common for lower-ranked AlphaFold structures to be less reliable and commonly disregarded in practice. Thus, we also considered the scores of each prediction’s best-ranked structure to inquire whether this improves the differentiation. AlphaFold structures are ranked by confidence score; however, Figure 2 shows that the confidence score is a poorer predictor than the other scores. We sorted the predictions by the average pLDDT score in the binding interface. As shown in Figure 3, considering only the structures with the highest pLDDT scores greatly improves the success rate of binders’ predictions; however, nonbinding structures also appear more confident than seen in Figure 2. Supplementary Figure S2 shows the corner pair plots of these parameters. Qualitatively, these plots all suggest a high degree of separation and predictability between binders and nonbinders.

We have established that AlphaFold has predictive ability, but the question remains “How error-prone is this predictability?” To answer this, we linked 2-client results to analogous 1-client results. Then, we utilized a genetic algorithm to train a composite threshold set for all 10 scoring parameters simultaneously. A receiver operating characteristic curve (ROC; plots of false-positive rate vs. true-positive rate of a given set of threshold values of the 10 parameters) was computed during the training, and the genetic algorithm optimized the area under the ROC (AUROC) of all threshold sets explored throughout the training. To ensure predictive quality, we generated learning curves (plotting the accuracy of the most accurate threshold found along the ROC) by training on different fractions of the available data and testing on the rest. Within each run, 10 iterations were completed on each fraction to enable the calculation of mean and standard deviations. Six replicates of this learning curve are shown in the Supplementary Presentation 3. The ROCs corresponding to the best iterations are also shown in the Supplementary Presentation 3. Of these, the iteration that achieved the highest AUROC was chosen to perform the analysis in the remainder of this paper. The final thresholds and their associated rates are provided in Table 1. It was possible to compute exhaustive ROC curves for each parameter individually. These are shown for comparison in the Supplementary Presentation 3. AUROCs can investigate the ability of the method to differentiate binders from non-binders (the perfect method has an area of 1, while random methods will have an area of 0.5). ROCs and AUROCs are provided in the Supplementary Presentation 3. Importantly, the AUROC for the composite threshold, considering all 10 scores simultaneously, is greater than that for any of the parameters alone, thus showing that it is the better method. The composite threshold achieves an AUROC value of 0.9274, whereas the best competitor (2-client LtoP) achieves an AUROC value of 0.9104. It should be noted that the entire parameter space for the composite threshold could not be exhaustively traversed, as is possible for the individual parameters. Exhaustively optimizing 10 dimensions of the parameter space is unfeasible. Consequently, although the complete ROC was found for each individual parameter, it is likely that the ROC found for the composite threshold system is not optimal, and the calculated AUROC represents a lower limit of the true AUROC.

**TABLE 1:** Parameters leading to the exclusive (low false pos.) and inclusive (low false neg.) predictions.

Exclusive	Parameter	Inclusive
>0.78	2-client Conf. Score	>0.62
>95.4	2-client Average pLDDT	>69.4
<4.97	2-client LtoP PAE	<4.80
<9.03	2-client PtoL PAE	<3.35
<0.922	2-client Dimer PAE	<1.103
>0.88	1-client Conf. Score	>0.77
>34.8	1-client Average pLDDT	>35.4
<8.14	1-client LtoP PAE	<11.30
<10.99	1-client PtoL PAE	<13.73
<1.158	1-client Dimer PAE	<1.249
5.5%	False-positive rate	27.9%
28.1%	False-negative rate	7.5%

We opted for two different thresholds, one that prioritizes low false positivity and the other prioritizing low false negativity. To make predictions for finding new LC8-binding partners, it is useful for a parameter set to minimize false positives while maintaining an acceptable false-negative rate. The exclusive threshold achieved a reduced false-positive rate of 5.5% combined with a false-negative rate of 28.1%, as shown in Table 1. On the other hand, a parameter set that minimizes false negatives while maintaining an acceptable false-positive rate can be useful for identifying elusive or noncanonical examples of binding sites in a protein that is known to bind LC8, from which our understanding of LC8 binding can be improved. The inclusive threshold has a false-negative rate of 7.5%, with a false-positive rate of 27.9%, as shown in Table 1.

This analysis alone is illustrative of the predictive ability of AlphaFold for differentiating between binders and nonbinders; however, several features of this analysis suggest that the predictability power might go beyond a simple binary and may even assess the relative stabilities of binders. This is consistent with findings that AlphaFold learned an energy function in the process of its training (Roney and Ovchinnikov, 2022; Herzberg and Moult, 2023; Ahdritz et al., 2024). Features implying this include (1) the difference between 1-client and 2-client scores, which track the cooperativity of the second LC8-binding event compared with the first, (2) the large population of binders that fail to be predicted to bind in the full set of predicted structures but are absent from the subset with only the best structure from each prediction, and (3) the presence of many of the false-positive predictions, which are AAA mutations of very strong binders.

### Affinity assessment

To evaluate whether AlphaFold predictions correlate with LC8-binding affinities, we extracted 62 sequences, eight amino acids long, with known LC8-binding affinities. This was compared with the list of binders, producing a list of 108 AlphaFold predictions with sequences present in the affinity list (some of the 62 are present multiple times). Although 62 affinities is not a small number experimentally, it is important to note that the sample space of possible LC8 binding sites is not well represented in this set. Thus, while the results may generalize well for some binders, it is expected that they may not generalize well for poorly represented binders.

**FIGURE 4:**
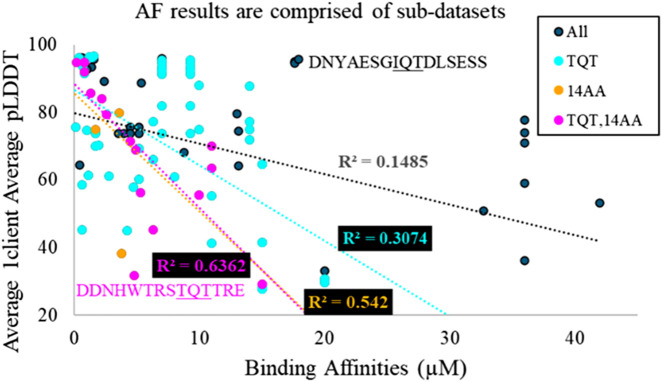
Overlaid scatter plots of four different sub-datasets of AlphaFold predictions. TQT refers to the anchoring triad of the motif. 14AA refers to the length of the sequence used in the AlphaFold prediction. The most general dataset has the worst fit, and the most specific dataset possesses the best fit. This implies that the general dataset is a heterogeneous aggregation of many smaller homogeneous datasets. Two outlier sequences are highlighted, from MUC4 in magenta and from Nup159 in black.

Experimental binding affinities were plotted against 21 scoring metrics in a grouping of 2 × 5 × 2+1 categories. To clarify, the best and average (2) of the confidence scores, average pLDDT of the binding interface, average LtoP PAE, average PtoL PAE, and average LC8 dimer PAE (5) of 1-client or 2-client predictions (2) were each plotted against binding affinities, and the coefficient of determination (R^2^) was calculated for the resulting scatterplot (Figure 4 for example). Additionally, the exclusive parameter threshold determined above was applied to all structures reported for each AlphaFold prediction to calculate success rates (success rate = # structures predicted to bind/# all structures in each prediction), which were also plotted against affinities. Given the heterogeneity present in the anchors of sequences with known affinities and of the context used in AlphaFold predictions, the analysis was completed for the full set of 108 and more homogeneous subsets, such as binders with TQT anchors, binders with different anchors, short predicted sequences, predicted sequences of specific lengths, and subsets meeting pairs of these criteria. Fit assessments are shown in Table 2 (additional subsets were considered but omitted due to low counts [<10]). Similar tables displaying RMSEs of these fits and p-values, corrected (Miller, 1991; Haynes et al., 2013) to avoid p-hacking, and assessing the likelihood of a non-zero correlation for each cell can be found in the Supplementary Presentation 2.

**TABLE 2:** R2 values for fits of different datasets correlating the scores of AlphaFold predictions to the corresponding experimentally measured affinities.

		Dataset	Full	TQT	!TQT	<18 AA	=16	=14	=12	TQT=16	TQT=14	TQT=12	!TQT=16	TQT!=12	TQT!=14	TQT!=12||14
		count	108	77	31	98	54	21	13	30	18	12	24	65	59	47
2client	Bests	Conf. Score	0.065	0.076	0.117	0.054	0.046	0.531	0.035	0.297	0.597	0.536	0.019	0.101	0.056	0.085
		Avg pLDDT	0.067	0.185	0.067	0.083	0.069	0.532	0.066	0.34	0.564	0.295	0.112	0.169	0.155	0.138
		L->P PAE	0.09	0.274	0.061	0.108	0.108	0.386	0.046	0.58	0.425	0.314	0.128	0.262	0.257	0.244
		P->L PAE	0.1	0.242	0.136	0.124	0.116	0.269	0.108	0.529	0.269	0.372	0.171	0.206	0.241	0.206
		Dimer PAE	0.054	0.221	0.00009	0.056	0.056	0.367	0.027	0.369	0.508	0.226	0.005	0.208	0.186	0.168
	Averages	Conf. Score	0.072	0.123	0.1	0.054	0.024	0.414	0.033	0.172	0.583	0.292	0.015	0.152	0.093	0.123
		Avg pLDDT	0.08	0.293	0.025	0.073	0.029	0.402	0.057	0.171	0.577	0.303	0.036	0.286	0.242	0.224
		L->P PAE	0.085	0.28	0.038	0.069	0.031	0.363	0.036	0.166	0.488	0.289	0.034	0.275	0.239	0.226
		P->L PAE	0.098	0.297	0.056	0.087	0.047	0.367	0.053	0.224	0.507	0.297	0.058	0.285	0.257	0.238
		Dimer PAE	0.01	0.112	0.017	0.01	0.004	0.372	0.013	0.1	0.562	0.226	0.008	0.104	0.075	0.064

		count	109	76	33	104	63	21	10	36	18	9	27	67	58	49
1client	Bests	Conf. Score	0.178	0.436	0.146	0.179	0.185	0.542	0.086	0.492	0.566	0.029	0.222	0.492	0.405	0.466
		Avg pLDDT	0.173	0.415	0.164	0.17	0.173	0.473	0.012	0.452	0.476	0.1	0.199	0.455	0.416	0.461
		L->P PAE	0.167	0.445	0.174	0.163	0.17	0.473	0.041	0.504	0.481	0.02	0.232	0.487	0.453	0.501
		P->L PAE	0.197	0.434	0.233	0.193	0.208	0.455	0.007	0.503	0.463	0.064	0.277	0.473	0.446	0.492
		Dimer PAE	0.147	0.434	0.042	0.143	0.182	0.479	0.127	0.609	0.579	0.026	0.098	0.484	0.417	0.483
	Averages	Conf. Score	0.154	0.294	0.171	0.155	0.152	0.522	0.143	0.325	0.636	0.001	0.241	0.339	0.235	0.273
		Avg pLDDT	0.149	0.307	0.203	0.146	0.145	0.542	0.172	0.316	0.636	0.0004	0.284	0.343	0.27	0.305
		L->P PAE	0.152	0.306	0.206	0.151	0.154	0.507	0.12	0.334	0.589	0.001	0.278	0.344	0.273	0.313
		P->L PAE	0.161	0.311	0.216	0.159	0.163	0.538	0.138	0.336	0.632	0.001	0.292	0.347	0.276	0.312
		Dimer PAE	0.099	0.333	0.012	0.094	0.082	0.488	0.079	0.388	0.696	0.014	0.02	0.375	0.273	0.323
Success Rate		correlation	0.25	0.413	0.321	0.218	0.142	0.477	0.243	0.119	0.575	0.615	0.303	0.402	0.37	0.336

“!” in column headers is the logic symbol NOT, TQT refers to the anchor sequence in the list of affinities, a number in a header indicates the length of client predicted by AlphaFold, “||” is the logic symbol OR. Row headers specify the scoring metric used, and whether it pertains to 1client or 2client predictions. “Bests” take the score from the best predicted structure. “Averages” take the average of the score across all structures for a given prediction. Success Rate takes the ratio of the number of structures that pass the threshold, and the number of structures generated for a given prediction. The color scale trends from red at the weakest R2, to white for central values, to blue for the strongest R2.

High R^2^ values mean high precision in predictions from the given model. The majority of these R^2^ values would not typically be considered quality R^2^ values. However, we must remember a few things: (1) we are investigating a utility that is not intended by the developers. (2) R^2^ is “the coefficient of determination” because it is the fraction of the variance in a dataset that is explained by the model: even R^2^ = 0.2 means that the given metric explains 20% of the variation in the data. (3) AlphaFold is a black box algorithm and is difficult to investigate directly, so any information for evaluating it is valuable. (4) AlphaFold struggles with certain cases, such as single amino acid changes (Pak et al., 2023) and fold-switching proteins (Chakravarty et al., 2024), so it is not surprising that AlphaFold predictions fail to explain a significant portion of the variation in affinities, particularly in cases where proteins switch from disorder to β-sheet and where single-amino acid changes are known to significantly impact affinity. (5) As shown in Figure 4, the overall AlphaFold dataset is a mixture of sub-datasets. As R^2^ values increase for subsets, it is likely that other subsets would also yield improved correlations compared to the full dataset.

Figure 4 highlights that, generally, a higher confidence prediction trends with stronger binders. As mentioned above, this trend is stronger in more homogeneous datasets. However, outliers exist in the broadest dataset and the most specific dataset. In particular, Figure 4 shows a high-confidence, weak binder from Nup159 and a low-confidence, strong binder from MUC4. For the MUC4-binding site, a Trp occupies position −5 of the LC8-binding motif. It is likely that AlphaFold perceives this residue as destabilizing, resulting in a low confidence prediction. The sequence from Nup159 is a weak binder but compensates for this via multivalency. LC8Pred assigns this sequence a poor volume and polarity (V&P) score, despite a decent amino acid score. AlphaFold’s assessment misses this key information regarding this binding site.

Although R^2^ is useful for linear relationships and intuitive for comparing two variables, it has limitations, such as its inability to assess non-linear relationships. There is no reason to believe that the correlations in this instance should be linear, so we analyzed the variables again, this time with mutual information. Mutual information (MI) is another way of assessing how well one variable can explain another variable; it reports how much information is shared between two sets of data. MI is related to Shannon information theory, and its calculation is similar to the calculation of Shannon entropy (Shannon, 1948). In this study, AMI was used to better handle small sub-datasets and to assess the strength of relationships. AMI modifies MI to account for expected random associations and has an upper bound of 1, unlike MI, which is unbounded. Although it is reflexive to ask, “What is a good value range?”, it misses the purpose of the analysis (as described in the previous paragraph) and of the technique. We are not aiming, primarily, to use these measures to determine whether affinity can be predicted (although that is of value); rather, we are investigating whether there are any indicators that AlphaFold has some understanding of binding energy. That being said, establishing lower limits for relevant values to consider is necessary for drawing conclusions. When comparing AMI to R^2^, lower AMI values are more credible in indicating a relationship than R^2^ values. Thus, while R^2^ > 0.4 is a strong indication of a relationship, AMI >0.2 is significant.

MI can only assess discrete variables: any analysis of continuous variables first requires binning the variables. We employed three binning strategies for both the affinities and the AlphaFold scores, which were each considered pairwise, yielding nine different AMI calculations with distinct bins. Binding affinities are typically compared with respect to the order of magnitude rather than linearly, so the three sets of bins used were set accordingly, considering both full and half orders of magnitude (10^0.5 = 0.316): [0, 0.316, 1, 3.16, 10, 31.6, 100], [0, 1, 10, 100], and [0, 0.316, 3.16, 31.6, 100], where each comma-separated value indicates a bin edge. For AlphaFold scores, bin edges were calculated for a geometric relationship, a linear relation, or with quantiles (bins all contain an equal number of items; bins will have varying widths). Bin edges were set based on the full dataset: sub-datasets were binned into the same bins as the full dataset to enable comparability across the datasets for a given binning strategy.

Many of the trends in Table 2 are also present in Table 3. The main exception is that AMI detects a relationship with sub-datasets where AlphaFold predictions use a 12-amino-acid-long client, whereas R^2^ analysis does not. This is true for the dataset with no specified anchor and the one with only TQT anchors. In both AMI and R^2^, each score on the datasets represented by the central columns (length = 14; anchor = TQT, length = 16; anchor = TQT, length = 14; and anchor = TQT, length = 12) exhibits a strong relationship with binding affinities. More subtly, both tables indicate that 1-client predictions have a closer relationship with affinities than 2-client predictions. The scores for each binning strategy separately are found in the Supplementary Tables 1-9.

Despite our statement that this analysis is not intended for achieving quality affinity predictions from AlphaFold calculations, it would be irresponsible to leave this potential application untouched. Both tables indicate that if the anchor of a binding site is TQT, AlphaFold predictions are best with a client that is 14 amino acids long, although clients 12 or 16 amino acids long will also enable decent predictions. Aside from these lengths, we did not run enough predictions to ascertain robust associations. However, if the anchor is not TQT, the predictions will be weaker because of the sparsity of affinities known for this type of LC8 client and the heterogeneity in the sequences of the motifs remaining in this dataset. For this type, only the length = 16 sub-dataset contained enough entries to analyze, and only four of the scoring metrics yield usable AMIs, namely, the best 1-client average pLDDT and PtoL PAE and the average 1-client average pLDDT and dimer PAE. It should be noted that if AMI relationships are used for predicting the affinity, predictions can only be made within the bins used in the analysis.

**TABLE 3:** Maximum AMIs for the different datasets between AlphaFold scores and corresponding experimentally measured affinities.

		Dataset	Full	TQT	!TQT	<18 AA	=16	=14	=12	TQT=16	TQT=14	TQT=12	!TQT=16	TQT!=12	TQT!=14	TQT!=12||14
		count	108	77	31	98	54	21	13	30	18	12	24	65	59	47
2client	Bests	Conf. Score	0.0550	0.0550	0.0470	0.0550	0.1010	0.1360	0.1620	0.2970	0.1380	0.2270	0.0700	0.0650	0.0690	0.1530
		Avg pLDDT	0.1470	0.2220	0.1460	0.1700	0.1950	0.2240	0.5520	0.5720	0.2090	0.6200	0.1620	0.2200	0.2550	0.2430
		L->P PAE	0.1430	0.2550	0.1470	0.2000	0.1890	0.0840	0.5520	0.5040	0.0680	0.6200	0.2000	0.2050	0.3210	0.2670
		P->L PAE	0.1090	0.1770	0.1210	0.1170	0.1330	0.1240	0.3610	0.3760	0.0930	0.4660	0.1260	0.1690	0.2740	0.3280
		Dimer PAE	0.0590	0.1550	0.0680	0.1100	0.0970	0.2310	0.4010	0.3940	0.1710	0.4330	0.1310	0.1780	0.1660	0.1960
	Averages	Conf. Score	0.0790	0.1300	0.1190	0.0830	0.0610	0.3330	0.1590	0.2060	0.5100	0.2720	0.1220	0.1780	0.1080	0.1290
		Avg pLDDT	0.0930	0.1410	0.1210	0.0720	0.1120	0.1950	0.4070	0.2450	0.2790	0.5180	0.1340	0.1610	0.1500	0.1740
		L->P PAE	0.0790	0.1470	0.1130	0.1080	0.1200	0.2820	0.3290	0.2290	0.5100	0.3820	0.1320	0.1720	0.1680	0.1780
		P->L PAE	0.1130	0.2030	0.0530	0.0910	0.1170	0.3380	0.3120	0.1900	0.4770	0.3820	0.1380	0.2330	0.2000	0.2290
		Dimer PAE	0.0650	0.1310	0.0970	0.1030	0.0780	0.1860	0.3290	0.2440	0.3760	0.3750	0.0890	0.1320	0.1890	0.1940

		count	109	76	33	104	63	21	10	36	18	9	27	67	58	49
1client	Bests	Conf. Score	0.1900	0.2390	0.1480	0.1900	0.2240	0.2210	0.2210	0.3400	0.3050	0.3440	0.1500	0.2640	0.2360	0.2670
		Avg pLDDT	0.1680	0.2220	0.1620	0.1650	0.1760	0.2240	0.1480	0.2830	0.2090	0.2180	0.2610	0.2410	0.2630	0.3210
		L->P PAE	0.1690	0.2310	0.1330	0.1650	0.1830	0.1650	0.3720	0.3380	0.2210	0.4910	0.1790	0.2500	0.2480	0.3180
		P->L PAE	0.1570	0.2260	0.1430	0.1550	0.2120	0.1650	0.1680	0.3210	0.1450	0.3440	0.3580	0.2460	0.2550	0.2760
		Dimer PAE	0.1180	0.2010	0.1010	0.1870	0.1420	0.3530	0.4060	0.2160	0.4290	0.5290	0.1240	0.1990	0.2170	0.2250
	Averages	Conf. Score	0.1320	0.2290	0.1060	0.1770	0.1640	0.3560	0.3240	0.3110	0.4940	0.1780	0.1600	0.2550	0.2420	0.2720
		Avg pLDDT	0.1100	0.1640	0.1250	0.1280	0.1620	0.3390	0.2820	0.2260	0.5250	0.1780	0.2250	0.1860	0.1820	0.2080
		L->P PAE	0.1350	0.1890	0.0930	0.1310	0.1340	0.4180	0.1850	0.2220	0.5210	0.1780	0.1730	0.1860	0.1890	0.2010
		P->L PAE	0.1030	0.1480	0.1370	0.0980	0.1160	0.4230	0.1850	0.1790	0.5250	0.1640	0.1900	0.1650	0.1600	0.1750
		Dimer PAE	0.0910	0.1560	0.1640	0.1190	0.1680	0.3090	0.3130	0.2440	0.3610	0.3490	0.2460	0.1520	0.1610	0.1660
Success Rate		correlation	0.1180	0.1690	0.1940	0.1380	0.0820	0.3090	0.5010	0.0990	0.5020	0.4800	0.2060	0.1960	0.1230	0.1380

Formats and headers match Table 2. AMIs shown are the maximum among all 9 binning strategies.

### Evaluating the possibility that AlphaFold has learned an energy function

**FIGURE 5:**
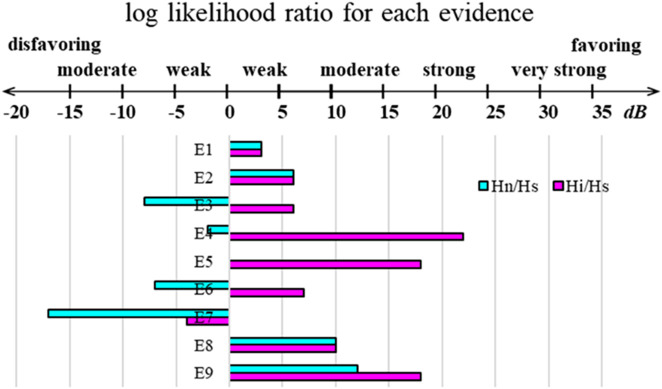
Qualitative Bayesian scale and summary plot of the logs of the ratios of likelihoods for each of the nine listed pieces of evidence for each discussed pairwise comparison. The X-axis is measured in decibels (dB).

Because it is a black box algorithm, accessing AlphaFold’s inner workings is difficult without a thorough, systematic investigation of its output. However, even without such a systematic investigation, our findings are more likely to align with certain hypotheses over others. We, therefore, utilized an approach that formalizes this type of thinking: qualitative Bayesian reasoning (Jaynes and Bretthorst, 2003; Fairfield and Charman, 2017; Fairfield and Charman, 2019). Qualitative Bayesian creates a framework in which pieces of evidence are systematically evaluated in the context of each hypothesis and then combined in a way that is grounded in mathematics and logic. With qualitative Bayesian, one posits mutually exclusive hypotheses and then establishes multiple pieces of evidence (**E**). With these in hand, each piece of evidence is judged for its likelihood while mentally inhabiting the universe of each hypothesis separately. Then, the relative likelihoods of the two hypotheses are compared pairwise. It is advantageous to express this comparison on a log-odds scale for a few reasons: (1) human perception works on a log scale (Fechner et al., 1966), so the spectrum from weakly to strongly favoring one hypothesis over another can be assigned a value on a log scale (with units of decibels, dB), as shown at the top of Figure 5, drawing inspiration from the measurements of sound; (2) log-odds allows for simple addition of the relative likelihoods of each piece of evidence; (3) this enables comparison between more than two hypotheses via simple subtraction of two pairwise comparisons to obtain the third; and (4) this mathematically based analysis reduces the potential for a judgment of one piece of evidence to bias the judgment of an unrelated piece of evidence because each is being assessed separately rather than as an aggregate. Using Bayesian inference in this manner is consistent with the methods established by the pioneers in Bayesian and qualitative Bayesian approaches (Jaynes and Bretthorst, 2003; Fairfield and Charman, 2017). We provided a more detailed discussion of Bayesianism and qualitative Bayesian in the *Methods* section.

The mutually exclusive hypotheses are as follows: during its training, **Hs** (structural): AlphaFold did not learn an energy function and only memorized structures. All predictions that appear novel are simply piecewise amalgamations of structures found in the training set. **Hn** (natural): AlphaFold learned the energy function that is at work in natural protein folding and interactions within some residual error. **Hi** (inaccurate): AlphaFold learned an inaccurate energy function that does not match the natural energy function but shares similar minima. When evaluating these hypotheses, we discuss each one separately and assess pairwise comparisons log(P(**Hn**)/P(**Hs**)) and log(P(**Hi**)/P(**Hs**)), following the format outlined in the *Methods* section. The value of log(P(**Hi**)/P(**Hn**)) is found by simple subtraction. For simplicity, we denote these comparisons as **Hn**/**Hs**, **Hi**/**Hs**, and **Hi**/**Hn** in the following discussion.

In assessing the evidence (**E**), let us consider each piece, one at a time. **E1**: As shown in Figure 2, 1-client scores are a better predictor of nonbinders than 2-client scores, consistent with the statement that the first binding event represents the energy barrier in LC8 binding. In the context of **Hs**, this is likely because when one copy of a client is present, part of the LC8 structure (β3) remains disordered without the second copy. The cooperativity of the second binding event is known to propagate through the structure of LC8, so this could lead to worse scores for 1-client predictions. In **Hn** and **Hi**, this event is slightly more likely because, in addition to the structural reasons, energetically the half-bound state is less stable than the fully bound state. Using the scale shown in Figure 5 as a guide, **Hn**/**Hs** and **Hi**/**Hs** are both 3 dB for this evidence.

**E2**: When considering all the structures produced from predictions of binders *versus* nonbinders, the 2-client scores of the binders cluster on the confident side of each plot, while the 1-client scores are bimodal, with the poorly predicted peak being populated by lower-ranked structures from the predictions of weak binders (Figure 2). In the context of **Hs**, this is a likely observation because of the bias toward more stable structures in the PDB. However, in both **Hn** and **Hi**, this is a highly likely circumstance because the proposed energy function in both cases can differentiate strong (approximate energy minima) from weak (higher energy structures) interactions. **Hn**/**Hs** and **Hi**/**Hs** are both 6 dB for this evidence.

**E3**: Depending on the cut-off used, one-sixth or one-half of the false positives are AAA mutants of strong LC8 binders. In **Hs**, this would be unlikely because the client folds as a beta-sheet upon binding, and according to the Chou–Fasman parameters (Chou and Fasman, 1974), a triple Ala stretch has a significantly higher alpha helix than beta-sheet propensity, while the TQT motif is favored to fold as a beta-sheet. This should be understood through structural training. In **Hn**, however, this observation would be even less likely; AAA mutations abolish LC8 binding. For **Hi**, this observation could be plausible; the other five amino acids are stabilizing for LC8 binding in any strong binder, and if either the quality of a TQT or the deleterious effect of AAA is underestimated, such an effect could be observed. For this, **Hn**/**Hs** is –8 dB and **Hi**/**Hs** is 6 dB, for evidence.

**FIGURE 6:**
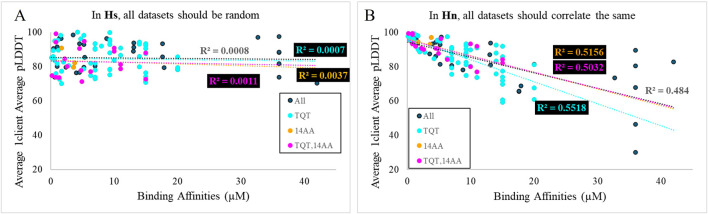
Plots of synthetic data representing the expectation for correlations of binding affinities and AlphaFold scores in the context of **(A)** Hs and **(B)** Hn.

**E4**: The scores of the full, heterogeneous dataset do not correlate with the binding affinities for each; however, the scores of several of the homogeneous subsets do correlate with affinities. In **Hs**, the first part of this piece of evidence is very likely, but combined with the second part of the statement, it becomes very unlikely. As shown in Figure 6A, all datasets should be random, but with high confidence, in the context of **Hs** because pLDDT should vary randomly, with no association to affinity. For **Hn**, the opposite holds true, where the homogeneous datasets would be expected to correlate, but the full dataset would be expected to correlate in the same way, as is shown in Figure 6B. For **Hi**, it is very likely for both things to co-occur. An inaccurate energy function will successfully exhibit correlations with affinities in datasets consisting of clients with similar properties; however, when several of these datasets are overlaid, their dissimilar correlations will obscure one another (Figure 4). **Hn**/**Hs** is −2 dB, and **Hi**/**Hs** is 22 dB.

**E5**: There is no correlation between success rates (using thresholds that minimize the false-negative rate) and affinities in the full dataset; however, correlations are found for some of the homogeneous subsets. **E5** is partly redundant with **E4** and, therefore, does not change the likelihood of **E4** in either **Hs** or **Hn**. However, the nature of the success rate calculation adds more potency to **E5** in the context of **Hi**. Taking a prediction set as a proxy of a solution at equilibrium containing 50 copies of the client peptide (or 50 pairs of client peptides in the 2-client version) and a successful prediction as “bound,” success rate = bound/50. Due to the small number of “molecules” and the significant probability that either “#bound” or “#unbound” could be 0, we cannot calculate a pseudo-Kd (#unbound^2^/#bound). However, it is important to note that in the range where solutions are valid, the success rate approximates a linear relationship with ln (pseudo-Kd), especially for success rates between 0.1 and 0.8. For context, ln(Kd) correlates linearly with ΔG. By extension, a relationship between the success rate and binding affinities approximates a relationship between pseudo-ΔG and ΔG. Thus, in the context of **Hi**, a relationship of this sort is very likely for homogeneous sub-datasets but not for heterogeneous datasets due to inconsistencies between the natural energy function and AlphaFold’s energy function in different settings. Therefore, in **E5**, the redundancy of information from **E4** dictates that **Hn**/**Hs** is 0 dB. However, the added layers make **Hi**/**Hs** an extra 18 dB for **E5**, in addition to that of **E4**, while still avoiding double counting redundant information.

**E6**: For TQT, length = 16 and TQT, length = 14 sub-datasets, affinities correlate well with individual scores. However, when considering the success rate, TQT, length = 16 has no correlation, while TQT, length = 14 remains strongly correlated. In the context of **Hs**, this is plausible because confidence in the local structure could be expected to increase unilaterally with increased context, with no regard for the relative binding stability. In **Hn**, **E6** is unlikely because energy differences between two lengths should correlate. In **Hi**, this is again likely because AlphaFold’s energy adjustment between these lengths could reasonably deviate from the reality of the experimental affinities. **Hn**/**Hs** is −7 dB, and **Hi**/**Hs** is 7 dB for **E6**.

**E7**: Several heterogeneous sub-datasets display reasonable R^2^ values but possess no relationship by AMI. This comes from one of the two phenomena: (1) the data resemble a transformed 1/x plot, where the leftmost points are mostly horizontal and the later points follow an approximately vertical trend, or (2) there is a high density of values with strong affinity with favorable prediction scores, but little to no trend beyond this dense region. In the context of **Hs**, **E7** is quite likely because strong binders are scored well, but outside this subset, there is no correlation. In the context of **Hn**, this observation is very unlikely because the values should correlate across the whole range. In the context of **Hi**, **E7** is likely because the remaining heterogeneity in the datasets results in multiple overlaid distinct relationships. **Hn**/**Hs** for **E7** is −17 dB, and **Hi**/**Hs** is −4 dB.

**E8**: Correlations between affinity and AlphaFold prediction are better in nearly all datasets for 1-client predictions than for 2-client predictions. This piece of evidence is nonredundant with **E2** because it goes beyond general observations, establishing a direct connection to affinities. In **Hs**, this is unlikely only because there should be no significant correlation. For **Hn** and **Hi**, the logic from **E2** holds: the 1-client prediction is more representative of an affinity calculated from an experiment in which the first binding event is rate-limiting. **Hn**/**Hs** and **Hi**/**Hs** are both 10 dB for **E8**.

**E9**: The “best” binning strategy is not consistent across sub-datasets. For example, binning affinity into [0, 0.316, 3.16, 31.6, 100] and scores into six quantiles yields AMI >0.4 for the majority of the 1-client average scores for the TQT, length = 14 but not for any of the 2-client best scores and is generally not correlative for the TQT, length = 12 dataset. In contrast, binning affinity into [0, 1, 10, 100] and the scores into six quantiles does the opposite, resulting in AMI > 0.4 for the majority of the TQT, length = 12 2-client best scores, but not for any of its 1-client averages scores and generally does not relate for the TQT, length = 14 datasets. In **Hs**, **E9** is unlikely because there should not be high AMI values as observed here, but if they were, it would be reasonable for them to be inconsistent. In **Hn**, this is likely. Correlations are expected, and deviations like this could be caused by random error or by a selection bias between the two sub-datasets. In **Hi**, **E9** is very likely because correlations are expected within homogeneous datasets, but because of the inaccurate energy function, they are expected to differ between different homogeneous datasets. **Hn**/**Hs** is 12 dB for **E9**, while **Hi**/**Hs** is 18 dB.

Taken altogether, **Hn**/**Hs** totals −3 dB, while **Hi**/**Hs** totals 86 dB. According to our analysis, **Hs** and **Hn** are approximately equally likely, but **Hi** is much more likely true than the other two. If we were to include a prior likelihood that favors **Hs**, to account for the expectation that AlphaFold should not be able to learn any energy function (−30 dB for **Hi**/**Hs,**) and especially not the function at work in nature (−45 dB for **Hn**/**Hs**), then **Hn**/**Hs** totals −48 dB, while **Hi**/**Hs** is 56 dB. This still strongly indicates **Hi** as the most likely true hypothesis of the three. In summary, mostly due to evidence statements **E4**, **E5**, and **E9**, we conclude that it is highly likely that AlphaFold has learned and uses an energy function in making its predictions which aligns with the true energy landscape at energy minima but is inaccurate elsewhere. Nonetheless, it is important to remember that there are many places where an inaccurate description of reality is useful for making predictions about reality. For instance, for many years, LC8-binding data have been simplistically analyzed using a model that assumes that both binding events are identical. Despite being an inaccurate picture of reality, this has still enabled years of useful research that resulted in useful lessons.

What does this mean for the field? AlphaFold possessing knowledge of an energy function is an exciting prospect: we have a tool for predicting both novel structures and their energetics and for predicting binding interactions. However, capitalizing on this prospect and applying it to another system will require a similar effort to the work done here in assessing predictions of binders and nonbinders because the energy function deviates from the natural function. Comparison of experimental data with AlphaFold scores from consistent, homogeneous datasets can be used as a Rosetta Stone to enable the translation of AlphaFold predictions into insights about the natural world. Caution is advised: evaluating the homogeneity of a dataset should be straightforward based on the strength of the correlations, but determining whether a predicted protein meets the characteristics defined by the homogeneous dataset may be more difficult. Additionally, it is likely that each time AlphaFold is trained again, it will converge on a new energy function, so each new iteration will require its own translation. Depending on the application, continuing to use older versions may be a reasonable strategy to circumvent this issue.

For LC8 interaction predictions, we need to expand the selection of known LC8-binding affinities so that more homogeneous datasets can be calibrated. LC8-binding sites that are not anchored by TQT (or a close variant) are not common, but there is a broad landscape of variety outside this motif. Additionally, motifs that are not anchored by TQT tend to have weaker affinities. These two factors together make such binding sites difficult to locate and characterize. However, as we expand the range of known, characterized binders, more homogeneous datasets can be assembled, and more correlations can be drawn to AlphaFold scores for predicting more binding sites and affinities.

### Prediction of new binding sites

The logical next step for our work was to take the lessons learned about AlphaFold’s ability to predict the binding sites for LC8 and apply them to predict previously unknown LC8-binding motifs in a selection of proteins. For this reason, we assembled a list of 24 proteins that match one of the following few criteria: (1) the presence of LC8 binding has been reported, but not the location of binding, (2) proteins that are known to bind LC8 but are not predicted by LC8Pred, or (3) LC8Pred predicts more binding sites than have been characterized experimentally. A list of these proteins and our impetus for predicting each can be found in the Supplementary Presentation 1.

We took these proteins’ full sequences and parsed them into 16 amino acid long segments to predict LC8 binding in AlphaFold. The resulting scores were compared with both the exclusive (low false positive) threshold and the inclusive (low false negative) threshold to generate lists of predicted binding sites. The exclusive threshold resulted in 247 predicted binding sites among the 24 proteins, while the inclusive threshold yielded 776 predicted binding sites. To narrow these predictions, we utilized the affinity correlations to calculate rough binding affinity predictions for each predicted binding site and used this to filter the predictions. If a binding affinity is 100 μM, it is not worth considering. Because the list of sequences with known binding affinities contains only one affinity greater than 40 μM, we disregarded predictions with predicted affinities greater than 40 µM to avoid extrapolating. For predicted binders with TQT anchors, the TQT, length = 16 (TQT = 16) 1-client best dimer PAE correlation was used, which has an RMSE value of 3.7 and is statistically significant according to Bonferroni (Miller, 1991) and Benjamini–Hochberg (Haynes et al., 2013) corrections to calculated p-values. For predicted binders, any other anchor, not TQT, length = 16 (!TQT = 16) 1-client average P- > L PAE correlation was used, which is significant according to the Benjamini–Hochberg p-value correction and has an RMSE value of 11.24 (tables of all p-values and RMSEs can be found in the Supplementary Presentation 2). Affinity filtering reduced the number of predicted binding sites to 133 for the exclusive threshold and to 213 for the inclusive threshold. Because the sequences used to develop the cut-offs were all from disordered regions and LC8 only binds to disordered regions, we also removed predicted sites within structured domains. The assignment of structured domains was taken from the AlphaFold Database (Jumper et al., 2021; Varadi et al., 2022).

**FIGURE 7:**
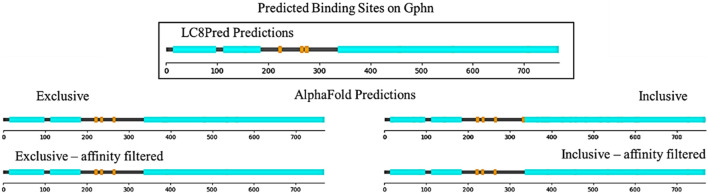
Sequence map of structured domains (cyan) and predicted binding sites (orange) on Gphn from LC8Pred and AlphaFold results as interpreted by each of the four score thresholds.

Figure 7 shows this analysis for gephyrin, a microtubule-associated protein that binds LC8 with an EDKGVQCE motif anchored at residue 224 (García-Mayoral et al., 2011). As shown, both AlphaFold and LC8Pred find this binding site and another anchored at 267, ISRGVQVL. Both methods find a third binding site, but at this point, the predictions are slightly different: AlphaFold finds a site anchored at residue 238 by VAS, which LC8Pred cannot find because of the alanine at position 0, whereas LC8Pred finds a site with a strong V&P score, but weak AA score anchored at residue 276 by an SLS triad. Figures for all 24 other predicted proteins are available in the Supplementary Presentation 1. Lists of each method’s predicted binding sites for each protein are provided in the Supplementary Datasheets 4–7.

**FIGURE 8:**
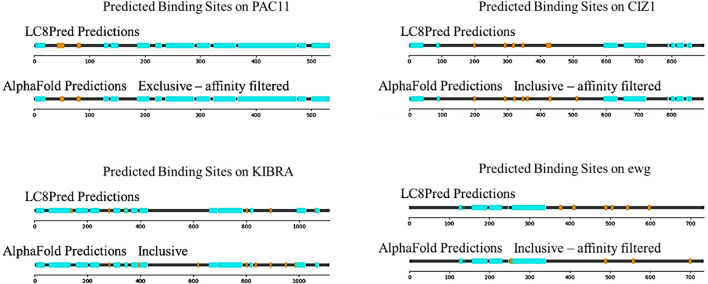
Sequence maps of the four predicted proteins PAC11, KIBRA, CIZ1, and Ewg. Structured domains (cyan) and predicted LC8-binding motifs (orange) are shown. For each protein, the LC8Pred predictions and one of the AlphaFold thresholds are included.

Figure 8 contains four more proteins, which were predicted by both LC8Pred and AlphaFold: PAC11, KIBRA, CIZ1, and ewg. These proteins were chosen to highlight different types of relationships between LC8Pred and AlphaFold predictions. In the case of PAC11, both predictions find the two binding sites that have been reported experimentally, which is an encouraging proof of concept for both methods. LC8Pred finds another site, but it overlaps with one of the known sites, so cannot bind. It must be noted that with our method, AlphaFold cannot predict overlapping binding sites because we only evaluate the scores of the best structure in each prediction. On KIBRA, both methods again accurately identify the two binding sites that have been shown experimentally, anchored at residues 283 and 892, but they each also identify new binding sites. Both methods predict a binding site anchored with a VMA triad at residue 802. AlphaFold scores provide a rough estimate of the affinity at 15 µM for this motif. AlphaFold predicts four more binding sites using the inclusive threshold: KVACVSAA anchored at 619, GRSSTQTL anchored at 836 (which LC8Pred assigns poor values to), SDSDSSTL anchored at 950 (predicted to bind with 2 µM affinity), and RSERLIRT at residue 985 (if confirmed, the first and fourth would represent unique motifs). Determining whether these sites bind will require testing their binding experimentally.

Cip1-interacting zinc finger protein (CIZ1) is an interesting case and is reminiscent of ASCIZ with its many predicted binding sites with various linker lengths between them and the presence of a zinc finger. Many of the same sites are predicted by both methods. The binding sites at residues 201, 294, 323, 348, and 430 are canonical binding sites with affinity estimates ranging from 1 to 20 µM. The binding site at 512 is also anchored by common triads, and LC8Pred detects it but scores it poorly, which is in line with AlphaFold estimates of affinity being on the weaker side at approximately 30 µM. However, the predicted binding site anchored at residue 361 is LQQKQVQP, another motif that deviates from the canonical LC8-binding motif and would be interesting to investigate further.

Ewg is one of the few examples in which LC8Pred predicts more binding sites than AlphaFold, highlighting the type of binders that AlphaFold may tend to miss. The LC8Pred predicted binding sites at 378, 411, and 598 have very strong V&P scores. This is further indication that AlphaFold fails to account for the information coded into the V&P matrix. The binding sites at 490 and 544 that are detected by both methods achieve low V&P scores but strong AA scores from LC8Pred, which further substantiates our observation. The additional AlphaFold predicted sites are an IQV- and a VQV-anchored motif.

Finally, there are proteins for which the LC8 binding site is experimentally determined, but LC8Pred cannot predict it, including pilA (Kausar et al., 2013), GCOM1 (García-Mayoral et al., 2011), PAK1 (Lightcap et al., 2008), TRPS1 (Kaiser et al., 2003), and MARK3 (Navarro‐Lérida et al., 2004). AlphaFold identifies the known binding site in all of these except MARK3. This is encouraging because it validates the aim of this paper to find a method that complements LC8Pred and enables the identification of elusive binding sites.

## Discussion

The collective information of protein folding and interactions represented by the algorithm AlphaFold complement LC8Pred and expanded the range of predicted binding sites. Our findings provide evidence that AlphaFold has learned an energy function capable of estimating trends in binding affinities for predicted binding sites. Our work emphasizes that for AlphaFold, this kind of analysis needs to be performed piecewise—broad, high-level views introduce data heterogeneity, obscuring the underlying patterns. We interrogate only one protein and its binding to a range of partners. Interestingly, this dichotomy in the scale of prediction is also present in the AlphaFold analysis: while some have utilized the confidence score to inform their conclusions (Bret et al., 2024) or warned against using PAE values as a benchmark (Lee et al., 2024), we found that the narrower metrics like PAE and pLDDT were invaluable for prediction, while the broader metric of confidence score proved less useful.

### Generalizability of the method

The approach we have developed should be generalizable to a host of different systems, including other hub proteins that have a broad range of partners, binders of SLiMs, or partners of IDPs. Research groups that require less stringent predictors could adapt this method with fewer data points than we used. However, the inclusion of non-binding examples is necessary for assessing the effectiveness of any predictive method. Reliance upon affinity correlations to obtain predicted non-binders is not strong enough given the types of fits and associated errors that we observed. It is important to apply the designed method in the context in which it was developed. In our case, all binding and nonbinding sequences are IDRs in the context of the full protein, so when predicting new binding sites, any predictions made in structured regions were deemed unreliable as such sequences were not represented in our training data.

A system-specific set of scores should be ascertained and investigated for how well they represent the system: scores of residues at or near the binding site are logical inclusions, but if other regions of the proteins are important for the interaction, domain knowledge should take precedence over attempts to best mimic our metrics. For PAE values, despite the instinct that errors should be similar from both directions, insight and differentiability can be gained by considering both directions independently. Importantly, there should be no expectation that thresholds will be at similar values for similar types of metrics; we have six different types of PAE values, and each of them behaves differently from one another based on the environment being investigated. The condensed structure of an LC8 dimer with one partner bound likely contributes to the threshold values being lower than they would be in a more elongated system. Readers should be aware that the most computationally intensive part of the process was in running AlphaFold on “unknown” binding regions to predict new LC8-binding sites because of the number of predictions that had to be made to adequately traverse a whole protein in 16-amino-acid-long segments. One could potentially investigate smaller sections of a protein if there is a justification to narrow the search: perhaps, a binding event will only occur at a disordered part of the sequence, in this case any structured parts of the protein can be ignored.

### Assessment of the binding status

Excitingly, we were able to achieve better-quality predictions of the binding status than expected based on recent work (Bret et al., 2024; Omidi et al., 2024; Lee et al., 2024; Chakravarty et al., 2024) which achieved lower AlphaFold prediction success rates than we achieved. However, as with all methods, there were inaccurate predictions for both binders and nonbinders. Supplementary Data Sheets 8-11 contains four tables listing false-positive and false-negative predictions for both inclusive and exclusive thresholds. The list of false positives for the exclusive threshold (false-positive rate = 5.5%) contains 17 predictions; of these, three are AAA mutants and 11 are from nonbinders used to train LC8Pred. For the inclusive threshold (false-positive rate = 27.9%), 86 sequences are falsely predicted, and the counts associated with each group are 43 and 25, respectively. This analysis is consistent with our finding that AlphaFold is likely to have learned an inaccurate energy function because the false-positive predictions mostly consist of sequences similar to LC8-binding sites, whether by retaining 5/8 residues of a known binder or sporting a common anchoring sequence.

On the other hand, the inclusive threshold produces 12 false-negative predictions, 7 of which are sequences from proteins sporting multiple LC8-binding sites. This indicates that the multivalent context overcomes unfavorability. Furthermore, six of the seven are from *Drosophila* ASCIZ. This is perhaps not unexpected, given that the heterogeneous nature of ASCIZ complexation with LC8 necessitates that it sports multiple weak binding sites. Some false negatives arise from sequence scans, and the binding site appears in two adjacent overlapping sections of the scan—one correctly predicted, the other not. These observations indicate that it is likely important to consider more context when making these predictions, whether from additional residues or from multivalent effects.

### Comparing our method with AlphaFold and LC8Pred

LC8Pred and AlphaFold perform similarly overall. Two distinct thresholds were also considered for LC8Pred, one reaching a 25% false-negative rate with a coupled 12% false-positive rate and the other having a 0% false-positive rate and a 43% false-negative rate. The accuracy of the less stringent threshold was 78%. For the methods reported in this study, the exclusive threshold has an accuracy of 86.7%, and the inclusive threshold has an accuracy of 79.1%. However, what is missing from this analysis is the fact that these two methods truly complement one another. The V&P matrix is an important feature of LC8Pred, and although the magnitude of these scores is smaller than the other half of the prediction, it could be said to be the more predictive of the two. Despite the importance of this metric, AlphaFold predictions tend to miss the binding sequences that are strongly indicated by V&P. Conversely because LC8Pred is built on the amino acid prevalence of known binders, it is limited to finding new binders that look like old ones.

### Consequences of the AlphaFold energy function

Before us, Roney and Ovchinnikov (2022), Herzberg and Moult (2023), and Ahdritz et al. (2024) all approached this problem through different lenses, but arrived at the same conclusion that AlphaFold learned an energy function during training. However, while Duignan (2024) proposed the theory, to our knowledge, our work is the first thorough assessment of the evidence concluding that AlphaFold learned an inaccurate energy function. Although not the initial impetus for this work, this finding reveals interesting consequences that must be considered when investigating AlphaFold’s utility for any type of analysis. Primarily, this results in situations where the analysis of large heterogeneous datasets is likely to lead to the conclusions of AlphaFold ineptitude. In these situations, the best practice would be to sort the full dataset into smaller homogeneous datasets, for example, split different classes of fold switching into smaller groups that can be considered independently, or separately analyze multiple smaller, internally consistent classes of pairwise amino acid mutations and use the defined subsets to find correlations in the system being investigated.

### Predicted binding sites

Many papers report interactions with LC8 without finding an interaction motif. Here, we predict likely binding sites for 24 proteins, including TRPS1 (Kaiser et al., 2003), CITFA (Kirkham et al., 2016), TbLAP (Zhang, 2019), and pilA (Kausar et al., 2013) (figures and tables of predictions can be found in the Supplementary Presentation 1 and Supplementary Data Sheets 4–7). Several of the predicted binding sites appear to defy the consensus sequence of canonical LC8 binding. However, this is expected and useful because there are several proteins reported to bind LC8 for which no binding site has been found, suggesting that an unknown recognition motif is present (Di Bartolomeo et al., 2010; Den Hollander and Kumar, 2006; Nakano et al., 2010; Rayala et al., 2005; Emi et al., 2005). Thus, these predictions supply a host of new sequences to test for LC8 binding to uncover these unknown binding modes. However, we are not beguiled by these predictions. We anticipate that many will not bind. We do, however, stress that even “failed” predictions contribute significantly to the field because there are not enough characterized non-binders in the literature for use in building prediction algorithms that are robust against false-positive predictions. All results are useful to increase the knowledge of the bond between AlphaFold scores and binding affinities, which enables iterative improvement of predictions.

**FIGURE 9:**
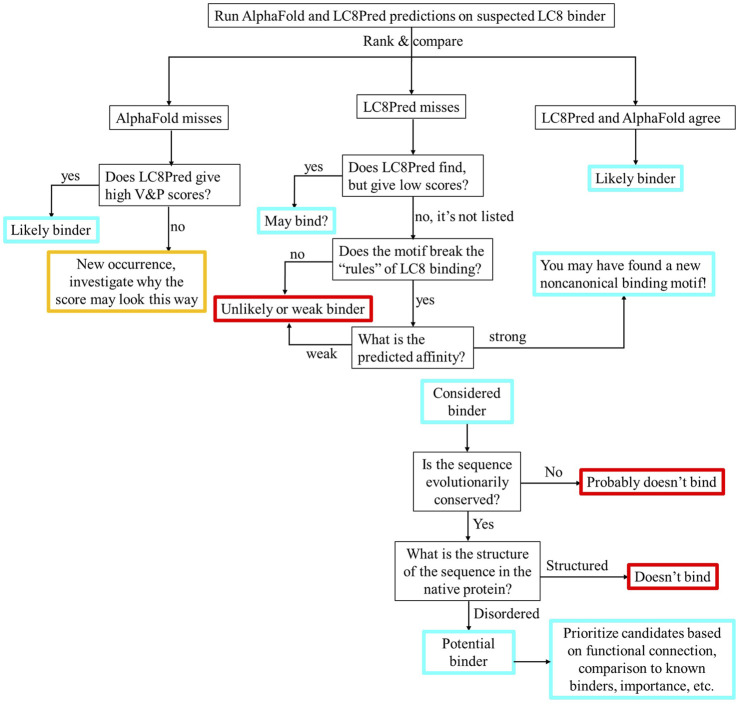
Flowchart for hypothesis design based on AlphaFold and LC8Pred results in combination. Routes ending in red likely do not bind. Routes ending in cyan in the top half will then pass through the lower half. The route ending in orange has not been observed; these scores would warrant consideration.

Our comparison of the results achieved by LC8Pred and AlphaFold suggests a scheme for using these predictions to generate hypotheses and guide experiments, as illustrated by the flowchart in Figure 9. After running LC8Pred and AlphaFold predictions (the most computationally intensive step), the thresholding methods should be applied to the AlphaFold results, and the predicted binding sites should be compared. Following this scheme should result in high-quality hypotheses. The second half of the flowchart provides an important set of steps beyond LC8-binding predictions: LC8-binding sites should be evolutionarily conserved and should not be located in structured regions of the full protein. Candidates that pass this second layer of winnowing should then be prioritized based on comparison to known binding sequences, functional overlap with LC8 and its partners, and the broader significance of the protein. This prioritized list can then be experimentally tested to identify new binders or to expand the dataset of nonbinders for training a successor to the LC8Pred model and refining the AlphaFold thresholds with additional data.

## Conclusion

In this work, we show that AlphaFold can accurately predict whether a protein binds LC8, with high success. The scores resulting from these predictions correlate with experimental binding affinities. Using Bayesian inference, we discuss evidence that AlphaFold has learned an energy function during training, although it is unlikely that this energy function resembles the natural energy function, except near minima, consistent with what has been proposed (Duignan, 2024). This work emphasizes the need to use homogeneous datasets for assessing AlphaFold’s ability to make energetic predictions. Heterogeneous datasets appear random because the resulting correlations are composed of several dissonant correlations. We then apply our findings to predict binding sites in 24 proteins known to bind to LC8. These predictions offer a path forward in identifying LC8-binding sites that deviate from the canonical binding motif, addressing a long-standing challenge in the field. Characterizing these predicted sites will be useful in designing and training less computationally expensive binding prediction algorithms.

## Data Availability

The scripts used here are available at https://github.com/DouglasRWalker/AlphaFold_BindingPrediction.

## Supplementary material

SUPPLEMENTARY DATASHEET 1 List of known binding affinities for partners of LC8. Protein name, 8-residue binding site, Kd, and PMID of the source are listed in each column.

SUPPLEMENTARY DATASHEETS 2, 3 First and second half of the list of all combined data aggregated from the results of all of the AlphaFold predictions run.

SUPPLEMENTARY DATASHEETS 4-7 Lists of the binding sites predicted by each threshold with and without affinity filtering. 4) Results from the affinity filtered, exclusive threshold. 5) Results from the unfiltered exclusive threshold. 6) Results from the affinity filtered inclusive threshold. 7) Results from the unfiltered inclusive threshold.

SUPPLEMENTARY DATASHEETS 8-11 Lists of the false positive and false negative predictions from each threshold. 8) False negative predictions from the exclusive threshold. 9) False positive predictions from the exclusive threshold. 10) False negative predictions from the inclusive threshold. 11) False positive predictions from the inclusive threshold.

SUPPLEMENTARY PRESENTATION 1 Images of the LC8Pred and AlphaFold predicted binding sites for each of the 24 proteins chosen to predict new binding sites. Reasons for selection are included for each.

SUPPLEMENTARY PRESENTATION 2 Tables of the statistical analysis of the linear regression fits between AlphaFold scores and binding affinities. Included are RMSEs and two tables of p-values with either Bonferroni or Benjamini-Hochberg corrections.

SUPPLEMENTARY PRESENTATION 3 Learning curves and receiver operating characteristics (ROC) curves for the different methods and combinations of thresholds.

SUPPLEMENTARY IMAGE 1 Corner plot of the distributions of the 10 sets of scores on the full set of structures from each prediction, showing the individual distributions and each pairwise distribution.

SUPPLEMENTARY IMAGE 2 Corner plot of the distributions of the 10 sets of scores on the set of the best structures from each prediction, showing the individual distributions and each pairwise distribution.

SUPPLEMENTARY TABLES 1-9 Tables of AMI values for the relationship between each type of score and the affinities of each subdata set for the different binning strategies. 1) Affinities binned by [0, 1, 10, 100] and scores binned according to a geometric strategy. 2) Affinities binned by [0, 1, 10, 100] and scores binned according to a linear strategy. 3) Affinities binned by [0, 1, 10, 100] and scores binned according to a quantile strategy. 4) Affinities binned by [0, 0.316, 3.16, 31.6, 100] and scores binned according to a geometric strategy. 5) Affinities binned by [0, 0.316, 3.16, 31.6, 100] and scores binned according to a linear strategy. 6) Affinities binned by [0, 0.316, 3.16, 31.6, 100] and scores binned according to a quantile strategy. 7) Affinities binned by [0, 0.316, 1, 3.16, 10, 31.6, 100] and scores binned according to a geometric strategy. 8) Affinities binned by [0, 0.316, 1, 3.16, 10, 31.6, 100] and scores binned according to a linear strategy. 9) Affinities binned by [0, 0.316, 1, 3.16, 10, 31.6, 100] and scores binned according to a quantile strategy.

SUPPLEMENTARY TABLES 10 List annotating the binding status of each protein used in the analysis as binder, non-binder, or unknown.

SUPPLEMENTARY TABLES 11 List of the results of linking 1 client and 2 client predictions for considering all 10 threshold values at the same time.

SUPPLEMENTARY FILE 1 Extended methods listing the scripts used for this project and describing their function so that others can use them for their own work.
