# Supplementary material for: Successful prediction of LC8 binding to intrinsically disordered proteins sheds light on AlphaFold’s black box
Source: Front Mol Biosci. 2025 Apr 23;12:1531793. doi: 10.3389/fmolb.2025.1531793 (PMC12057147; doi:10.3389/fmolb.2025.1531793)
Supplement: Supplementary file 6 [file Supplementaryfile1.docx]

Successful prediction of LC8 binding to intrinsically disordered proteins illuminates AlphaFold’s black box

Douglas R. Walker^1^, Gretchen Fujimura^1^, Juan M. Vanegas^1^, Elisar J. Barbar^1,*^

1. Department of Biochemistry and Biophysics, Oregon State University, Corvallis, OR 97331, USA

**SUPPLEMENTAL METHODS**

Python scripts used:

- parse_fasta.py: For parsing long protein sequences into smaller sections for more precise identification of LC8 binding sites and lower, tenable scores for analysis a python script parse_fasta.py was employed which reads in a full protein sequence and pulls out a subsequence of the protein, adds the necessary LC8 sequences, and creates a new .fasta file for the prediction. This code was modifiable to handle creating 1 or 2 copy versions of the .fasta file, to extract 12 or 16 amino acid long stretches, and to include 8 or more amino acid overlap between adjacent parsed sequences.
- analyze_structures.py takes .fasta sequence file, .pdb structure file, and .json scores files of each ranked structure to find a sequence of the intrinsically disordered partner that is bound to the LC8 dimer binding groove and extracts the relevant scores from the .json file. Important scores include the confidence score, the average of the PAE across the interface between the two LC8 protomers in a dimer, the average pLDDT score of the amino acids in the binding sequence, the average PAE of the LC8 binding site with relation to the binding sequence, and the average PAE of the LC8 binding sequence with relation to the binding site. Extra readout from this script includes a list of all pairs of alpha carbons within 5 Å of one another, individual amino acid scores for each amino acid and amino acid pairs in binding interactions. All this information for each potential binding sequence that fits the criteria of the algorithm for detecting binding sites (<5 Å between alpha carbons of an LC8 and IDP to start which then are part of a stretch of 8 amino acids for which the alpha carbons are <7.5 Å from an LC8 alpha carbon) is included.
- organize_analysis.py takes the readout of analyze_structures.py for each structure of a full prediction, extracts the most important values from the best scoring sequence and organizes this for easy aggregation with all the other sequence predictions.
- run_output_analysis.py goes into the subfolders of the current folder that correspond to each prediction output located therein, and runs analyze_structures.py on each structure, followed by running organize_analysis.py on each set of predictions, thereby expediting the process.
- combine_and_analyze_all_datasets.py searches subfolders for files with “analysis_summary.csv” in the title to combine them into a master .csv file with identifiers of client type, WT or AAA, binding status, full sequence, and off-target analysis. For this, every prediction output folder was inspected and a .txt file named either binder.txt or nonbinder.txt was manually created for each entry. An additional .csv file which lists and identifies each structure containing an off-target predicted binding site was manually curated.
- link_2to1clients.py is a necessary script because while all the predictions for a set of with 1 client or with 2 client proteins are pre-associated with each other due to all being predicted together, the same is not true between the two sets, but analyzing the results of the two sets together affords very useful, discriminatory information. This script reads through full combined_analysis_data.csv file and associates results from the same sequences in the appropriate order of scoring rank.
- plot_analysis_data.py takes the results of link_2to1clients.py and plots bar plots and pair plots of the predictions of various desired subsets of the results. It was modified to plot all results, the top 20% of the results for each prediction set, or only the very best result of each prediction set. Additionally, this script takes the filtered result set specified and calculates a false negative and false positive rate of the predictions given a particular cutoff threshold for the set of scores associated with each result. Different combinations of score thresholds were investigated manually to achieve threshold combinations for optimal false positive and false negative rates for a given application.
- learning_curve_validation_GA.py utilizes the differential_evolution method from the scipy.optimize package to train a high performing classifier. The data was divided randomly into training data and testing data at defined fractions (10%, 20%, …, 90%) and a genetic algorithm was used to optimize the cumulatve AUROC across all thresholds visited. For each defined fraction, 10 iterations of randomizing and genetic algorithm optimization were done to calculate a mean and standard deviation of both training data and testing data performance along a learning curve. Consideration of the learning curve determined whether we had sufficient data to reach performance plateau. At the end of this process, the 90% trained iterations were compared and the iteration with the highest AUROC (without considering the performance of the test data) was used to determine the thresholds used for subsequent parts of this paper.
- affinity_analysis.py scanned through the AlphaFold predictions looking for sequence matches among known binders. When sequence matches were found, all the pertinent scores we have described were extracted and included in new .csv files
- mutual_info.py took the .csv files output by affinity_analysis.py to accomplish its calculations using the sklearn48 package for calculating mutual information. This script binned the values of both the affinities and the scores in question. For each scoring parameter, identical bins were used for each data subset to enable direct comparison between dataset evaluations.
- pred_bind_extract.py searches through all the assembled AlphaFold runs, looking for predictions of sequences which are unknown as to whether they bind to LC8. The combination of all AlphaFold scores assessed were compared to the cut-off thresholds that were established in this work. Structures that achieve the cut-off threshold are predicted to bind LC8 with the false positive and false negative rates associated with each threshold set of values.
