# Supplementary material for: Successful prediction of LC8 binding to intrinsically disordered proteins sheds light on AlphaFold’s black box
Source: Front Mol Biosci. 2025 Apr 23;12:1531793. doi: 10.3389/fmolb.2025.1531793 (PMC12057147; doi:10.3389/fmolb.2025.1531793)
Supplement: Supplementary file 7 [file Presentation1.pptx]

## Slide 1
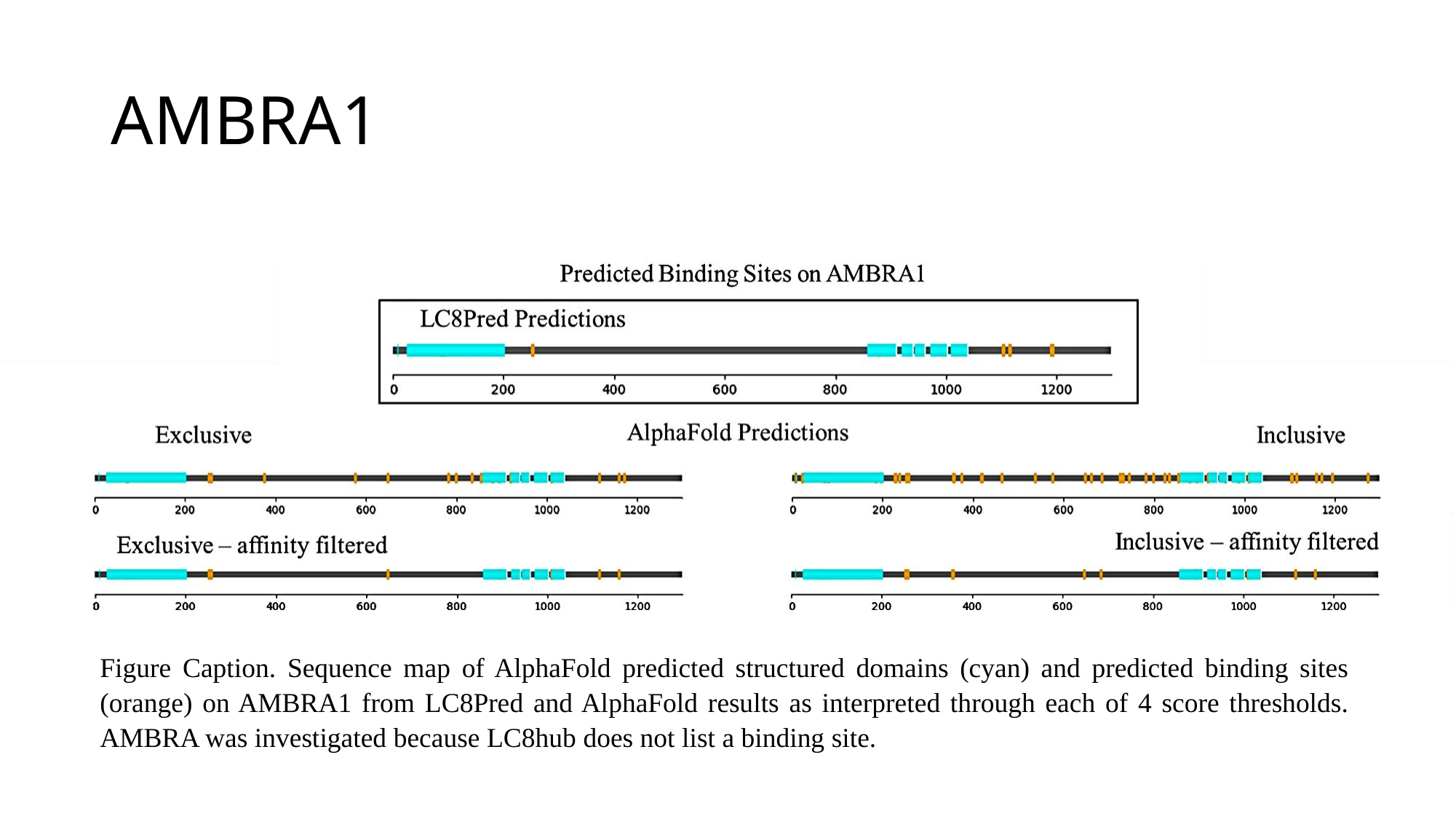

# AMBRA1
Figure Caption. Sequence map of AlphaFold predicted structured domains (cyan) and predicted binding sites (orange) on AMBRA1 from LC8Pred and AlphaFold results as interpreted through each of 4 score thresholds. AMBRA was investigated because LC8hub does not list a binding site.

## Slide 2
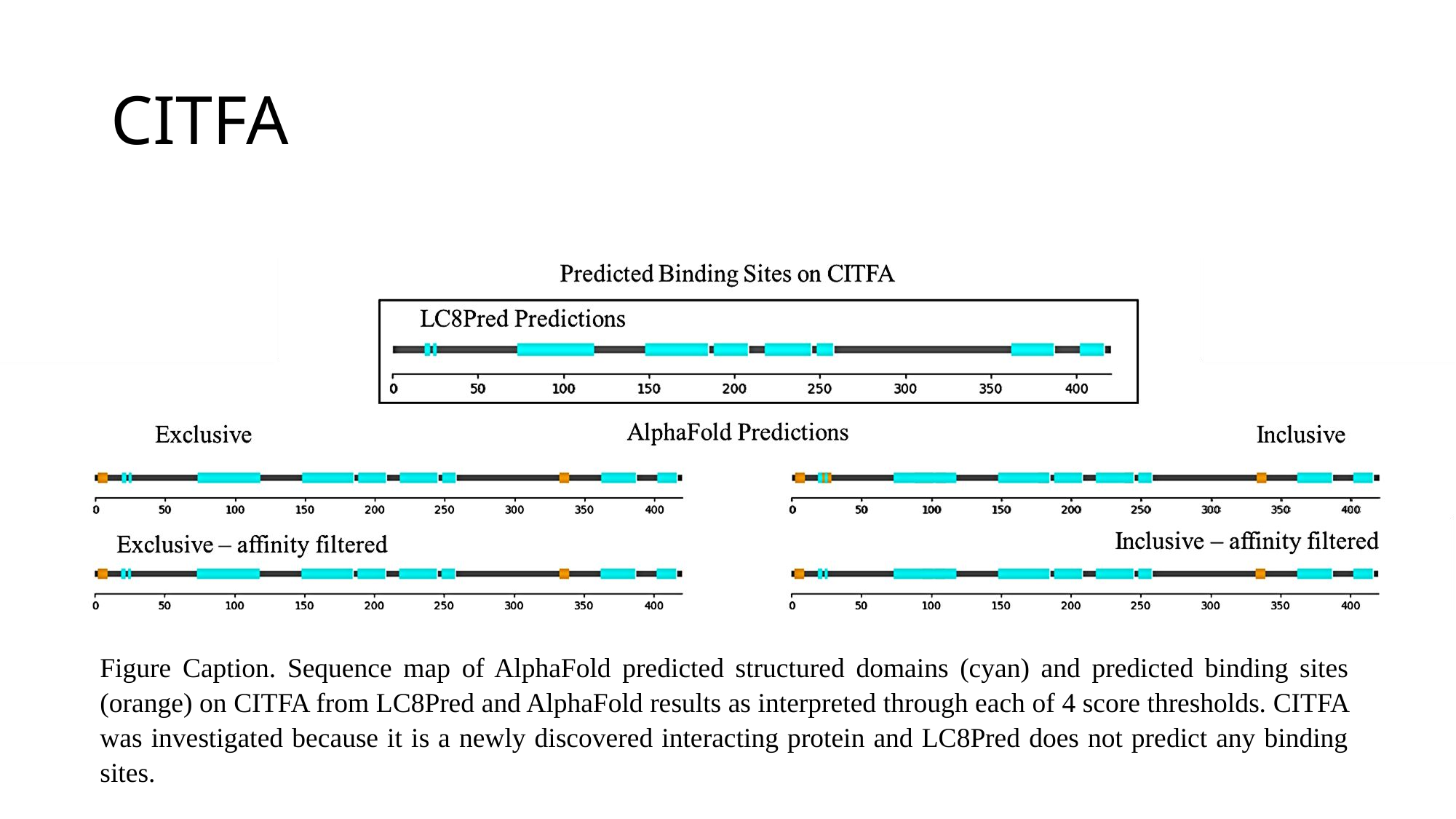

# CITFA
Figure Caption. Sequence map of AlphaFold predicted structured domains (cyan) and predicted binding sites (orange) on CITFA from LC8Pred and AlphaFold results as interpreted through each of 4 score thresholds. CITFA was investigated because it is a newly discovered interacting protein and LC8Pred does not predict any binding sites.

## Slide 3
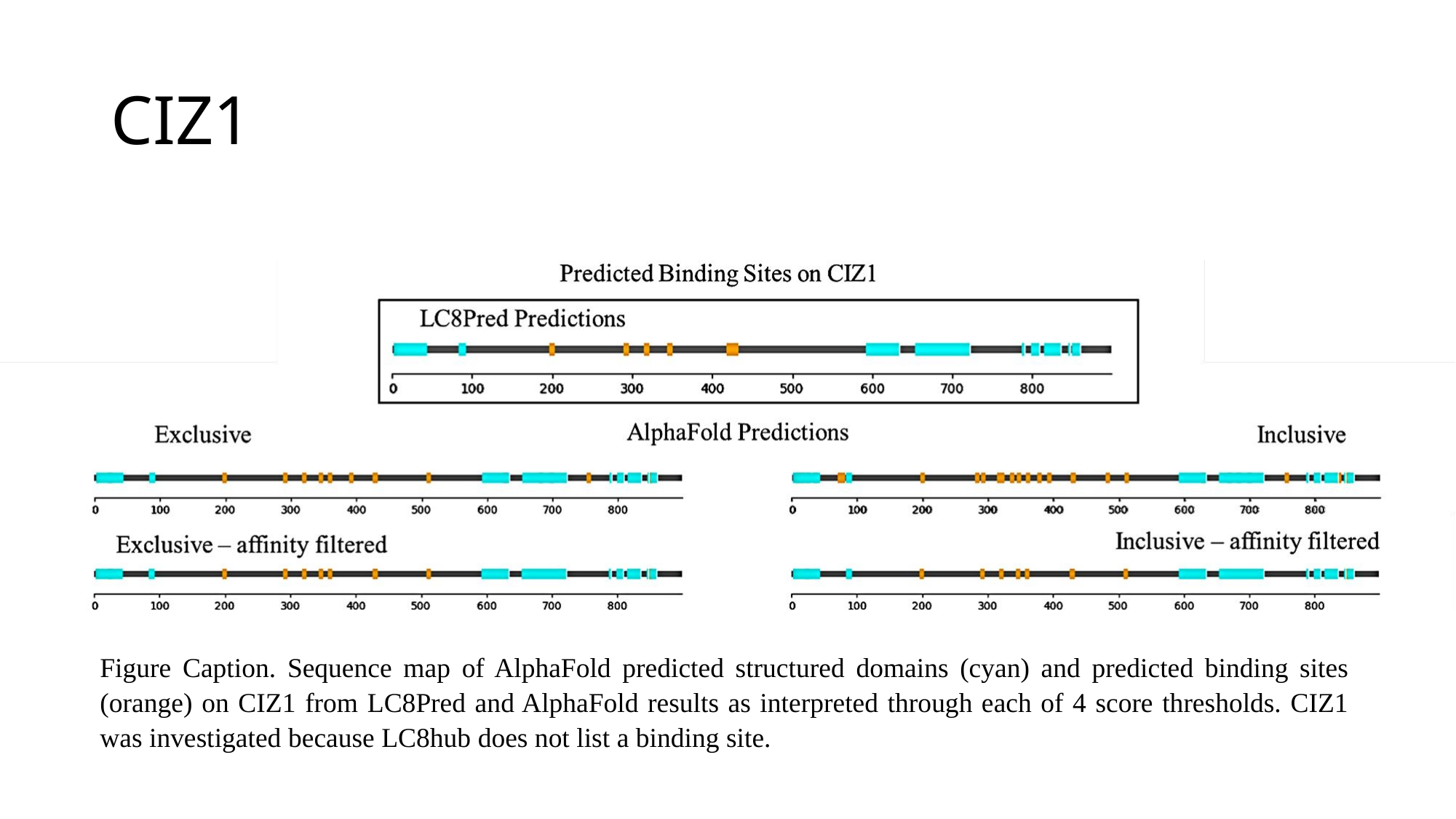

# CIZ1
Figure Caption. Sequence map of AlphaFold predicted structured domains (cyan) and predicted binding sites (orange) on CIZ1 from LC8Pred and AlphaFold results as interpreted through each of 4 score thresholds. CIZ1 was investigated because LC8hub does not list a binding site.

## Slide 4
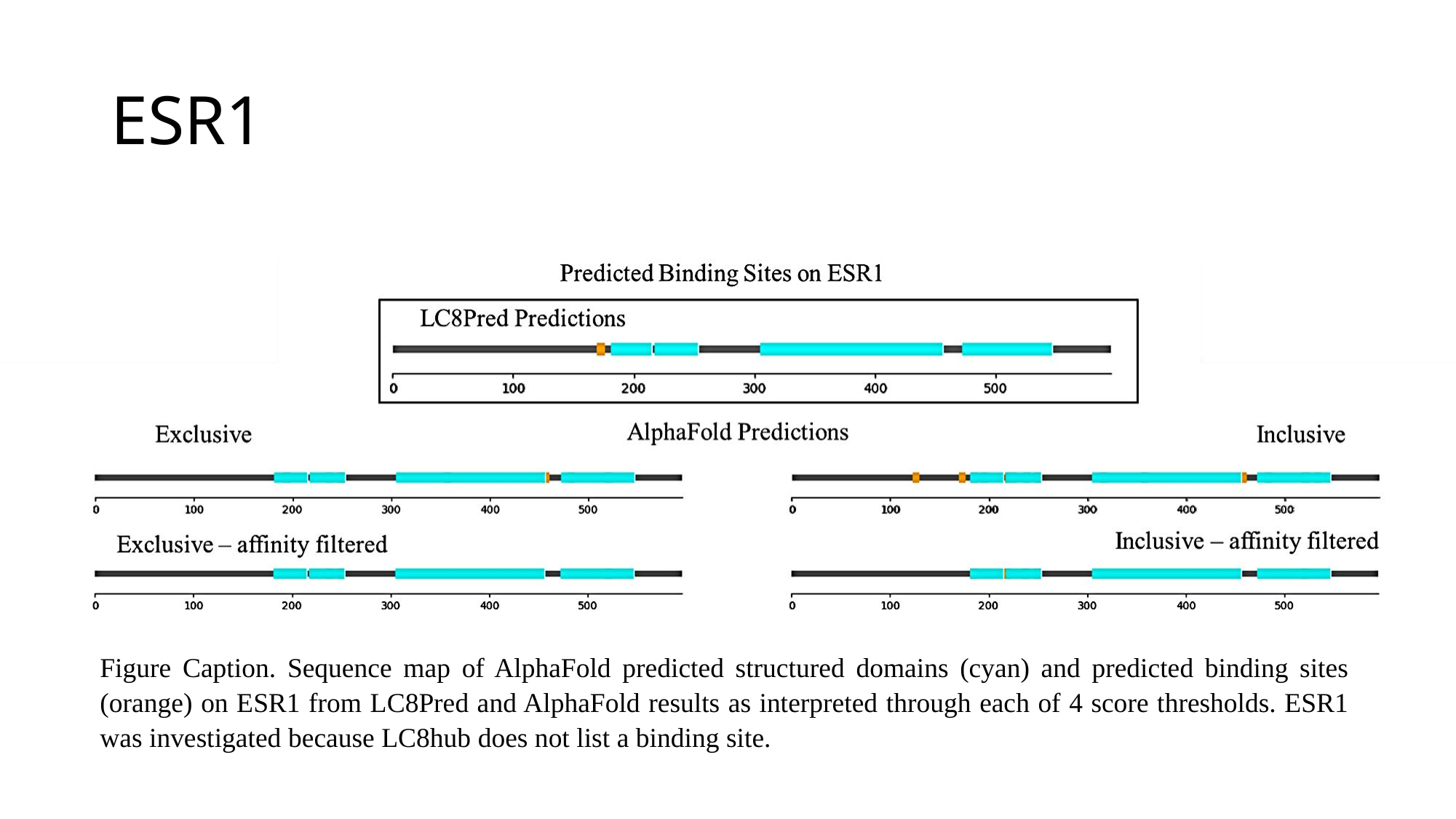

# ESR1
Figure Caption. Sequence map of AlphaFold predicted structured domains (cyan) and predicted binding sites (orange) on ESR1 from LC8Pred and AlphaFold results as interpreted through each of 4 score thresholds. ESR1 was investigated because LC8hub does not list a binding site.

## Slide 5
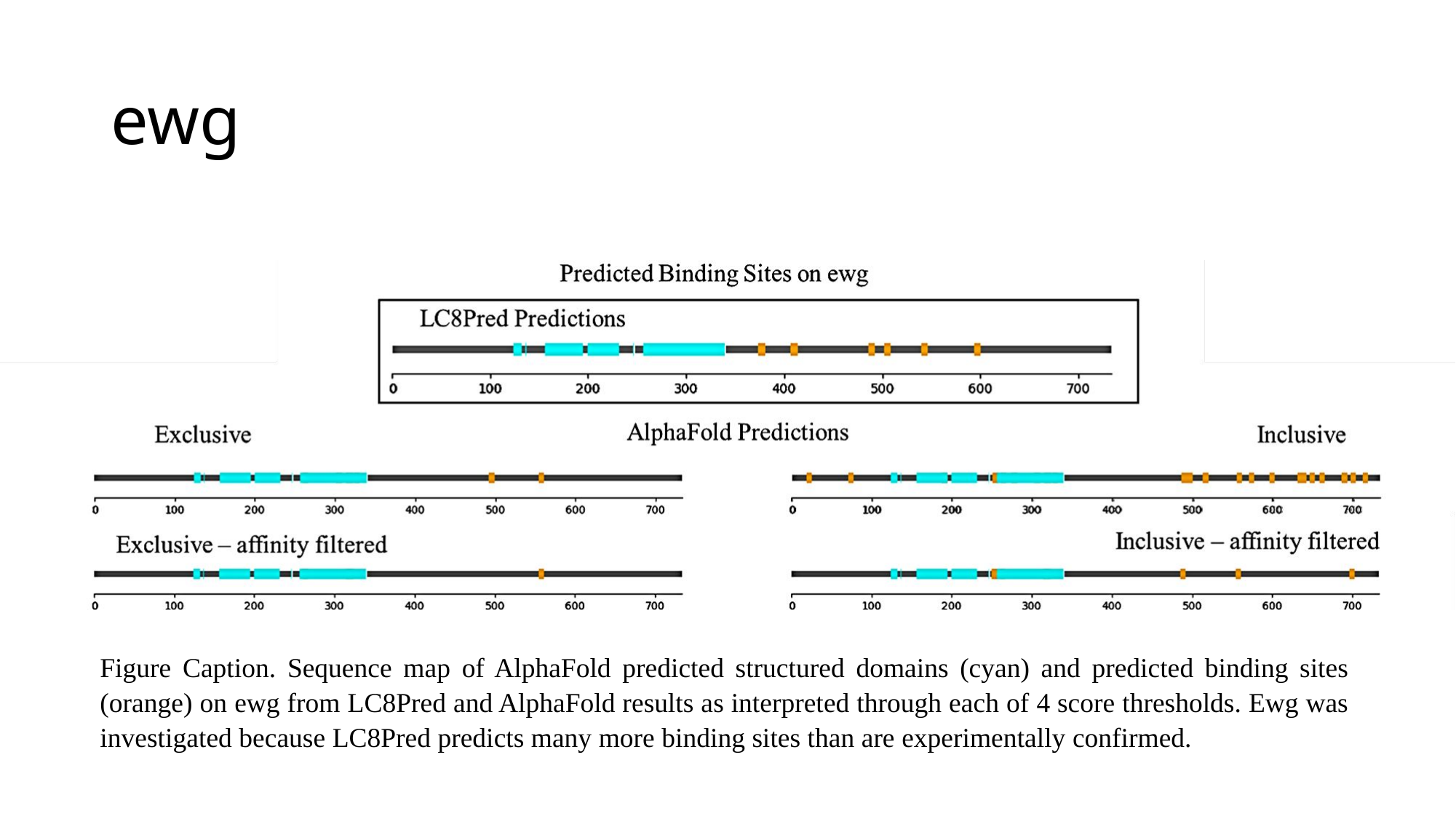

# ewg
Figure Caption. Sequence map of AlphaFold predicted structured domains (cyan) and predicted binding sites (orange) on ewg from LC8Pred and AlphaFold results as interpreted through each of 4 score thresholds. Ewg was investigated because LC8Pred predicts many more binding sites than are experimentally confirmed.

## Slide 6
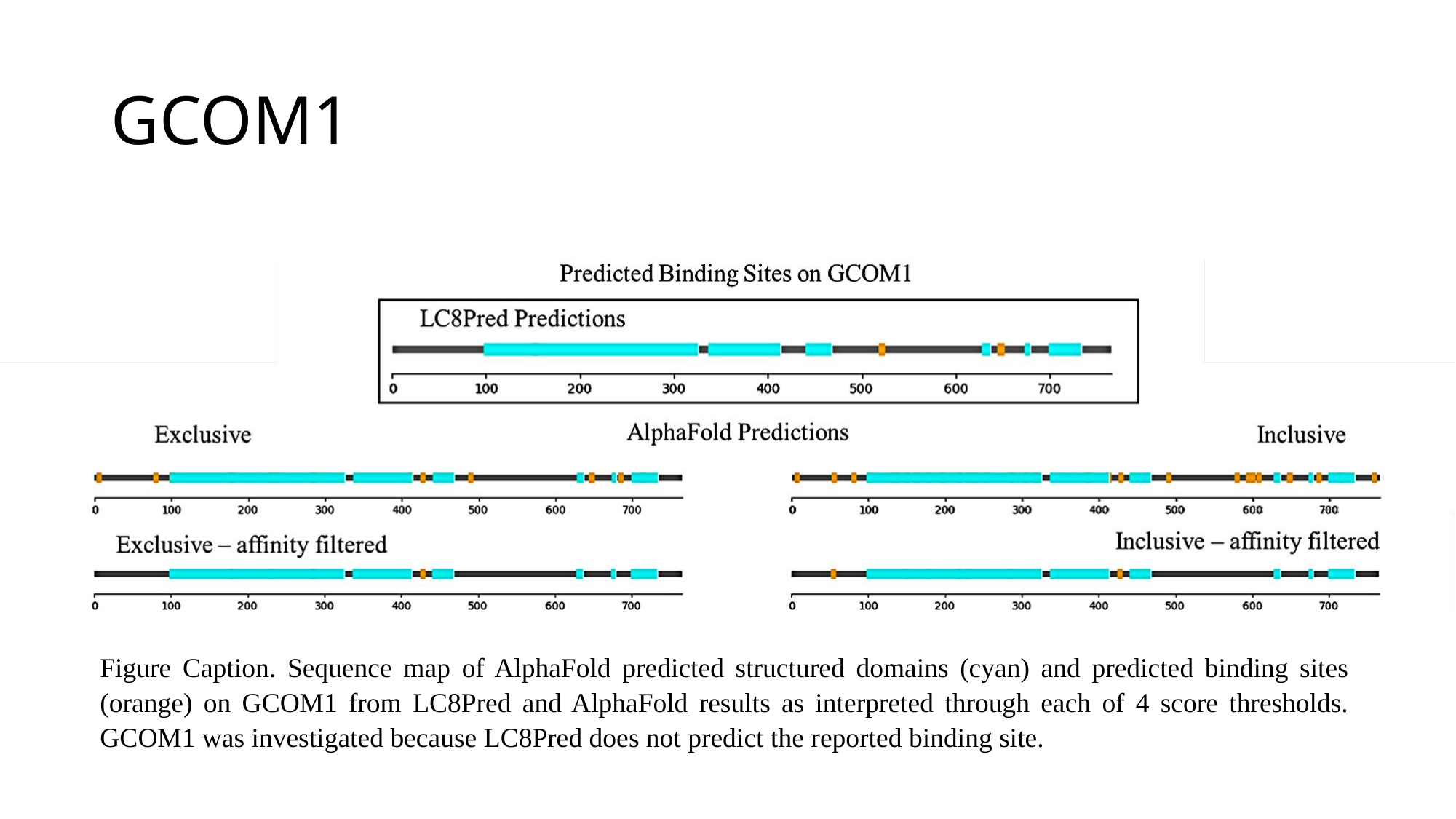

# GCOM1
Figure Caption. Sequence map of AlphaFold predicted structured domains (cyan) and predicted binding sites (orange) on GCOM1 from LC8Pred and AlphaFold results as interpreted through each of 4 score thresholds. GCOM1 was investigated because LC8Pred does not predict the reported binding site.

## Slide 7
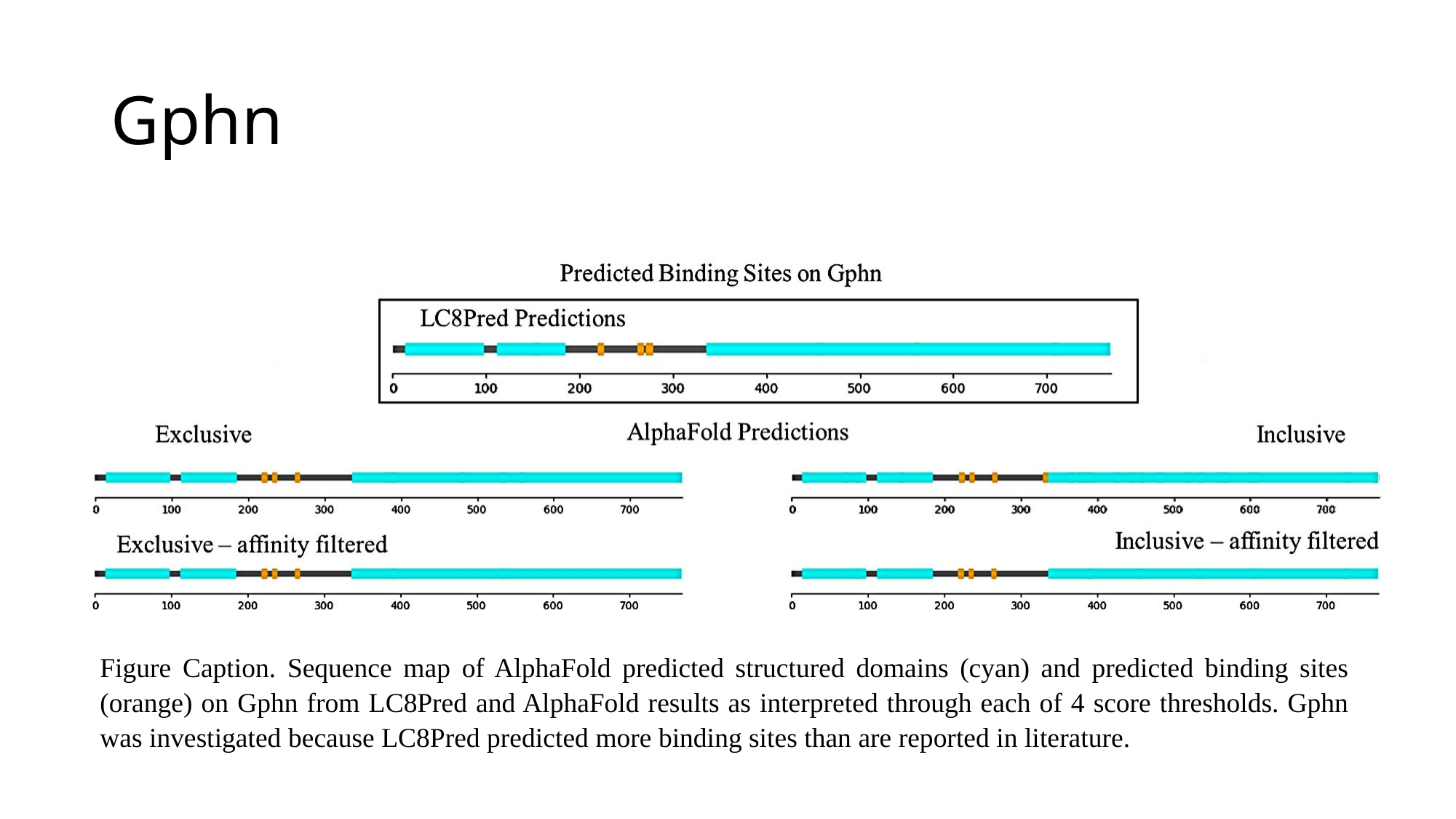

# Gphn
Figure Caption. Sequence map of AlphaFold predicted structured domains (cyan) and predicted binding sites (orange) on Gphn from LC8Pred and AlphaFold results as interpreted through each of 4 score thresholds. Gphn was investigated because LC8Pred predicted more binding sites than are reported in literature.

## Slide 8
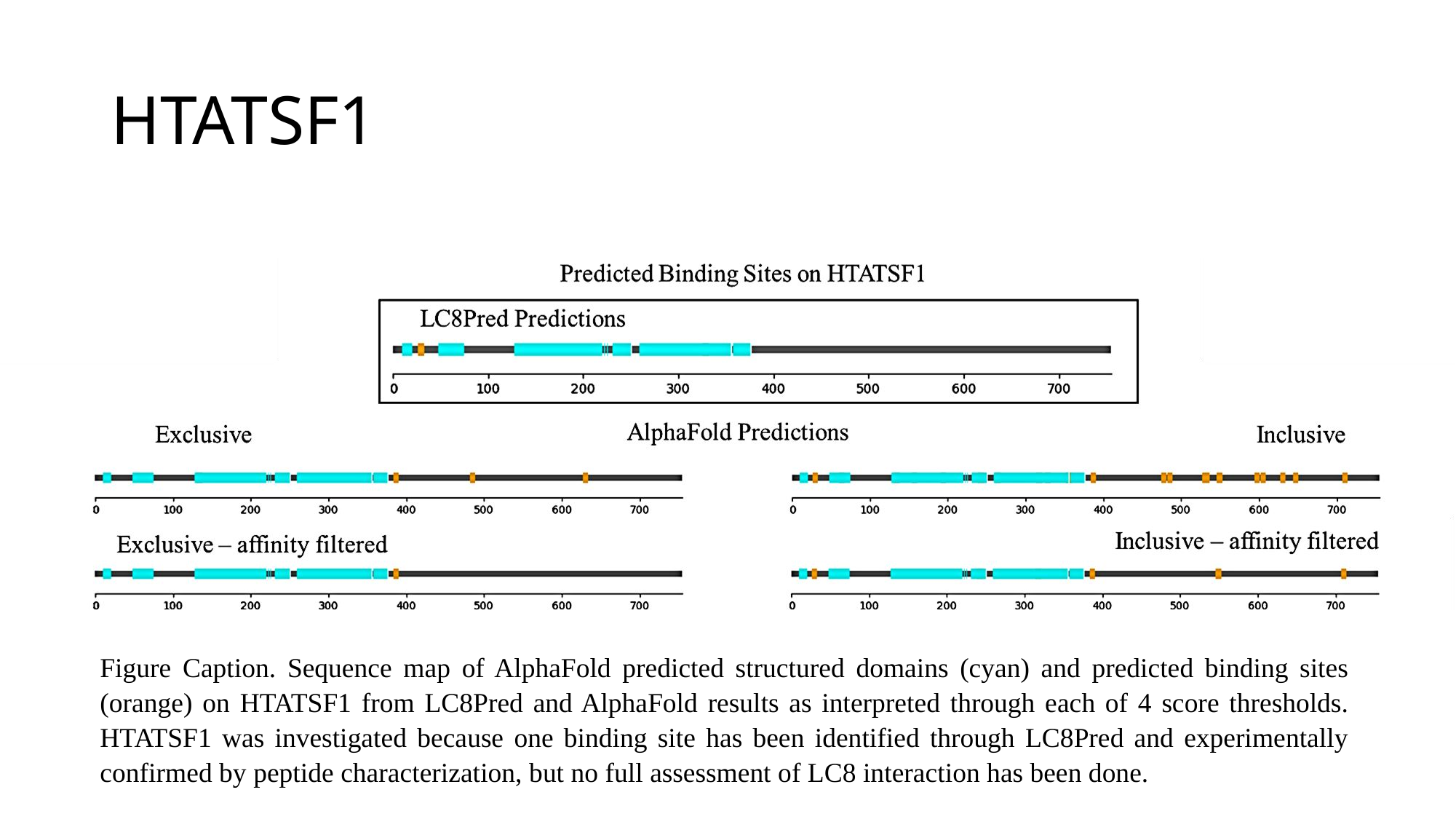

# HTATSF1
Figure Caption. Sequence map of AlphaFold predicted structured domains (cyan) and predicted binding sites (orange) on HTATSF1 from LC8Pred and AlphaFold results as interpreted through each of 4 score thresholds. HTATSF1 was investigated because one binding site has been identified through LC8Pred and experimentally confirmed by peptide characterization, but no full assessment of LC8 interaction has been done.

## Slide 9
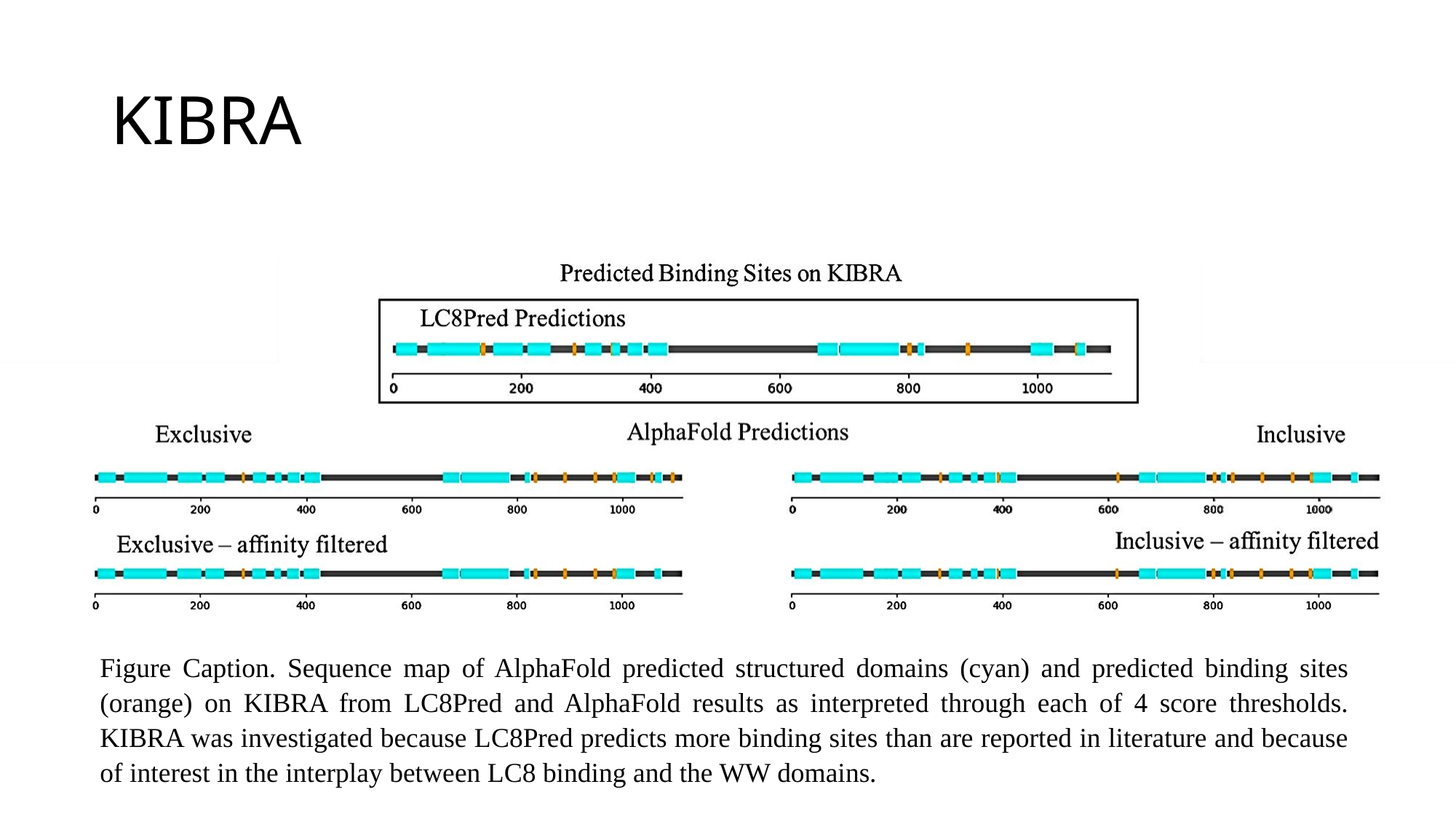

# KIBRA
Figure Caption. Sequence map of AlphaFold predicted structured domains (cyan) and predicted binding sites (orange) on KIBRA from LC8Pred and AlphaFold results as interpreted through each of 4 score thresholds. KIBRA was investigated because LC8Pred predicts more binding sites than are reported in literature and because of interest in the interplay between LC8 binding and the WW domains.

## Slide 10
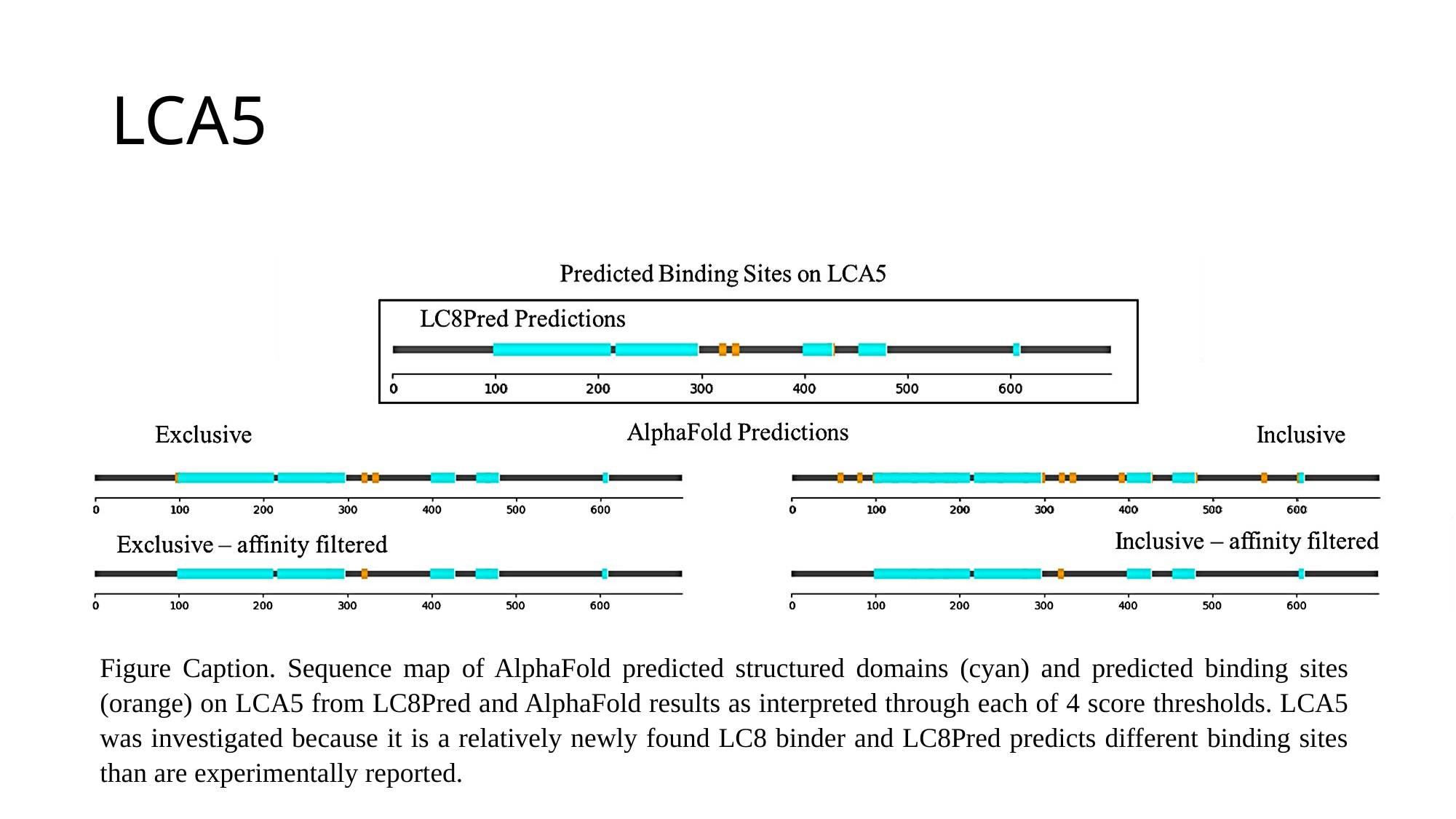

# LCA5
Figure Caption. Sequence map of AlphaFold predicted structured domains (cyan) and predicted binding sites (orange) on LCA5 from LC8Pred and AlphaFold results as interpreted through each of 4 score thresholds. LCA5 was investigated because it is a relatively newly found LC8 binder and LC8Pred predicts different binding sites than are experimentally reported.

## Slide 11
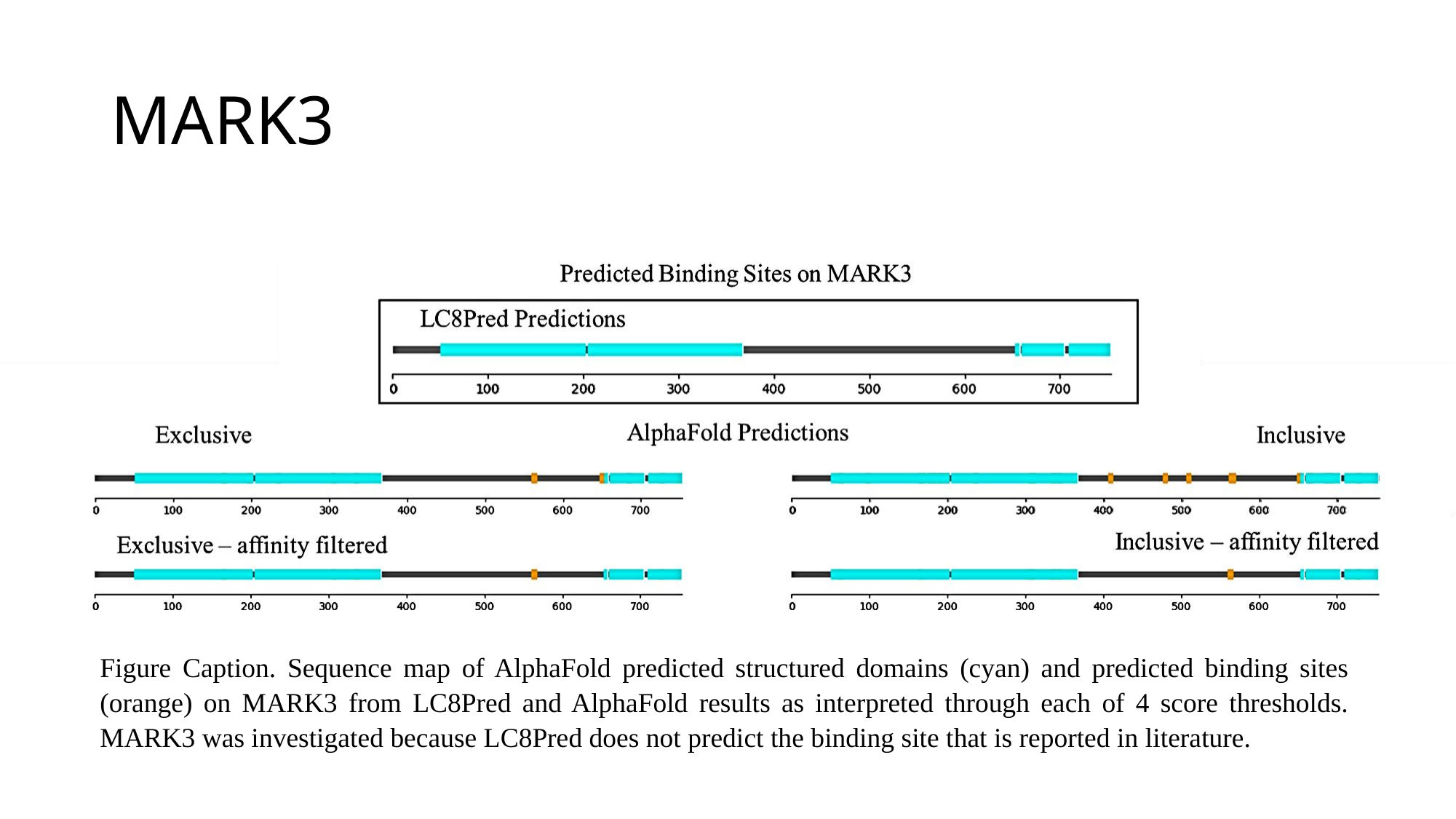

# MARK3
Figure Caption. Sequence map of AlphaFold predicted structured domains (cyan) and predicted binding sites (orange) on MARK3 from LC8Pred and AlphaFold results as interpreted through each of 4 score thresholds. MARK3 was investigated because LC8Pred does not predict the binding site that is reported in literature.

## Slide 12
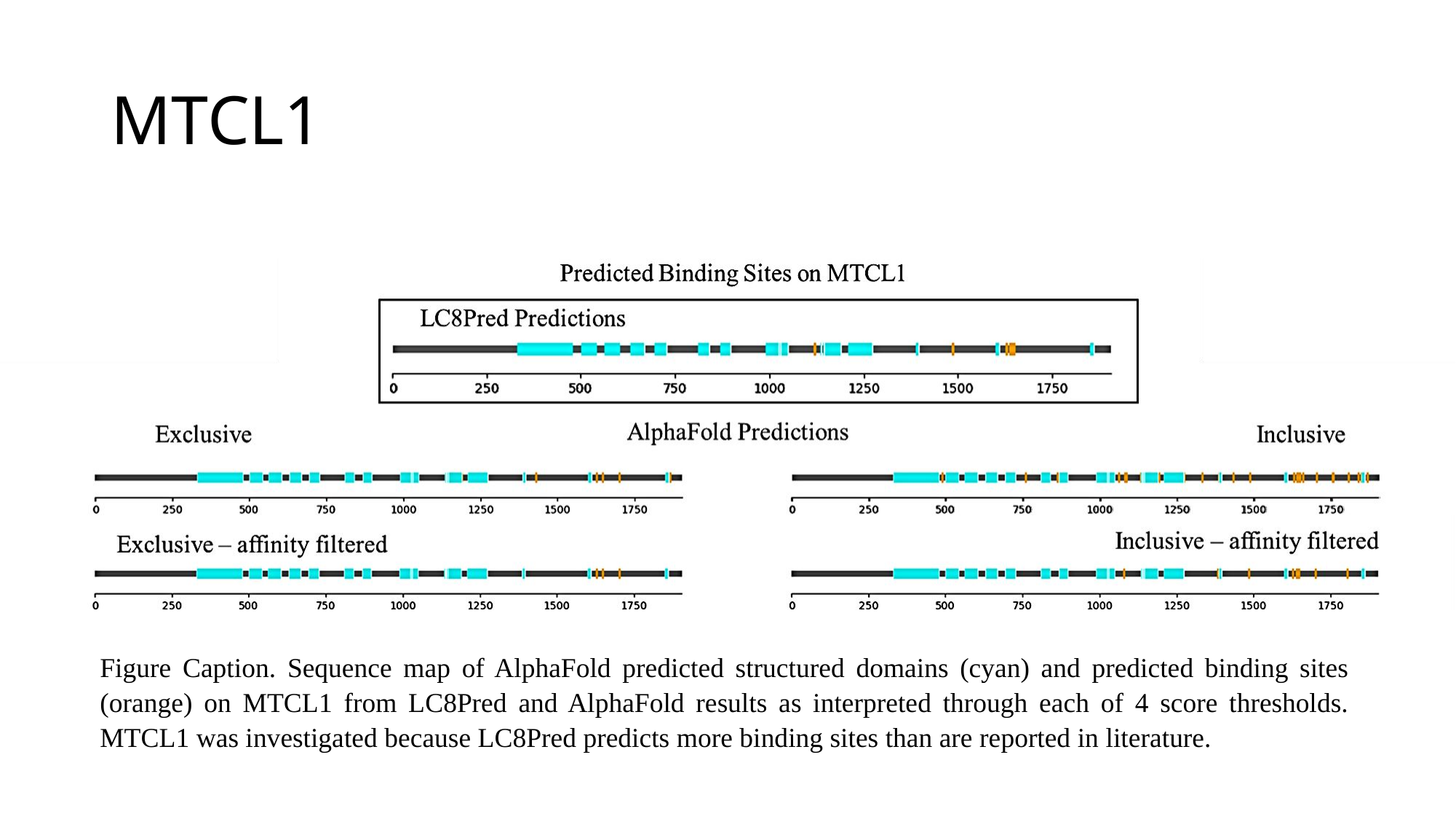

# MTCL1
Figure Caption. Sequence map of AlphaFold predicted structured domains (cyan) and predicted binding sites (orange) on MTCL1 from LC8Pred and AlphaFold results as interpreted through each of 4 score thresholds. MTCL1 was investigated because LC8Pred predicts more binding sites than are reported in literature.

## Slide 13
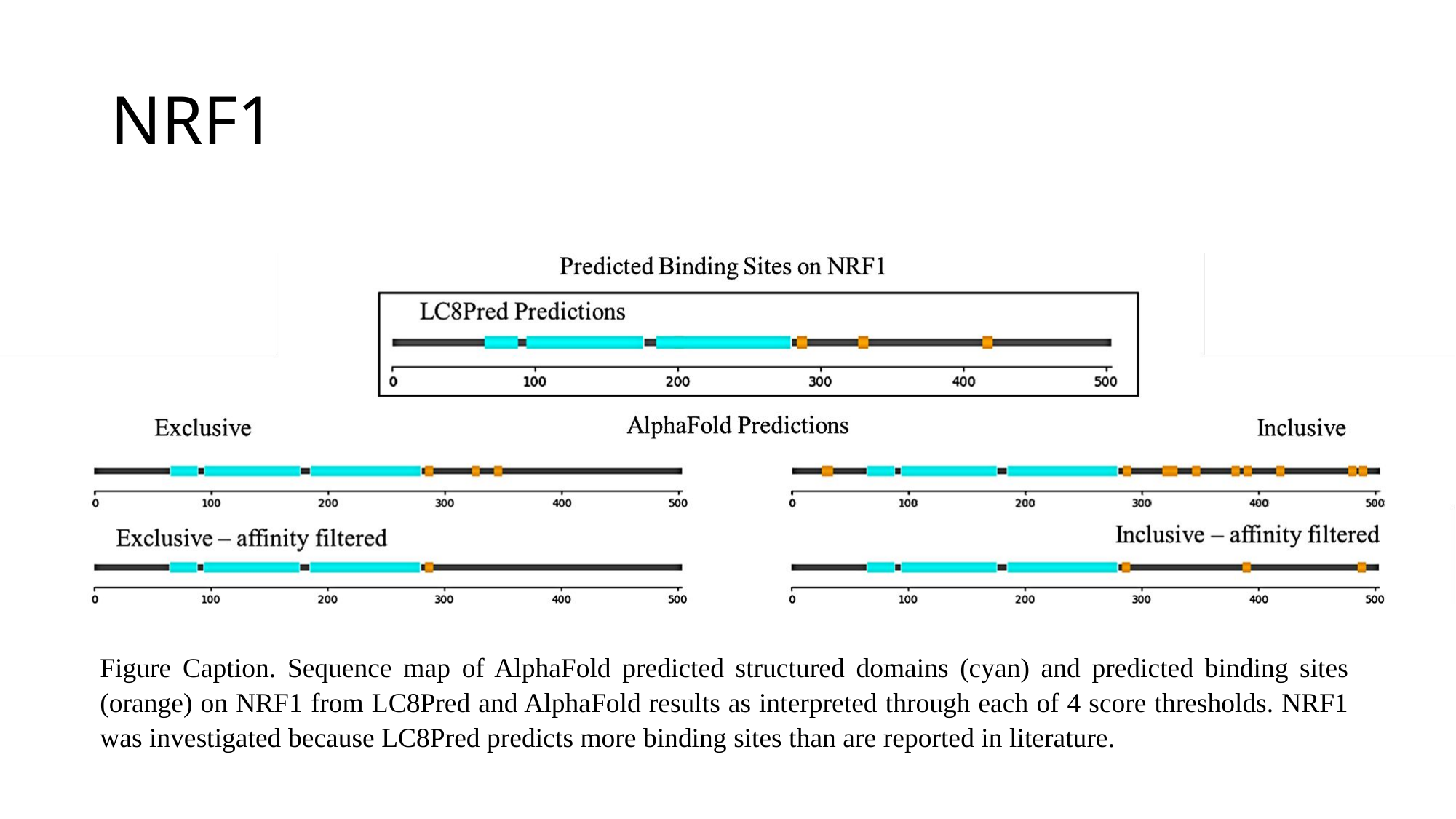

# NRF1
Figure Caption. Sequence map of AlphaFold predicted structured domains (cyan) and predicted binding sites (orange) on NRF1 from LC8Pred and AlphaFold results as interpreted through each of 4 score thresholds. NRF1 was investigated because LC8Pred predicts more binding sites than are reported in literature.

## Slide 14
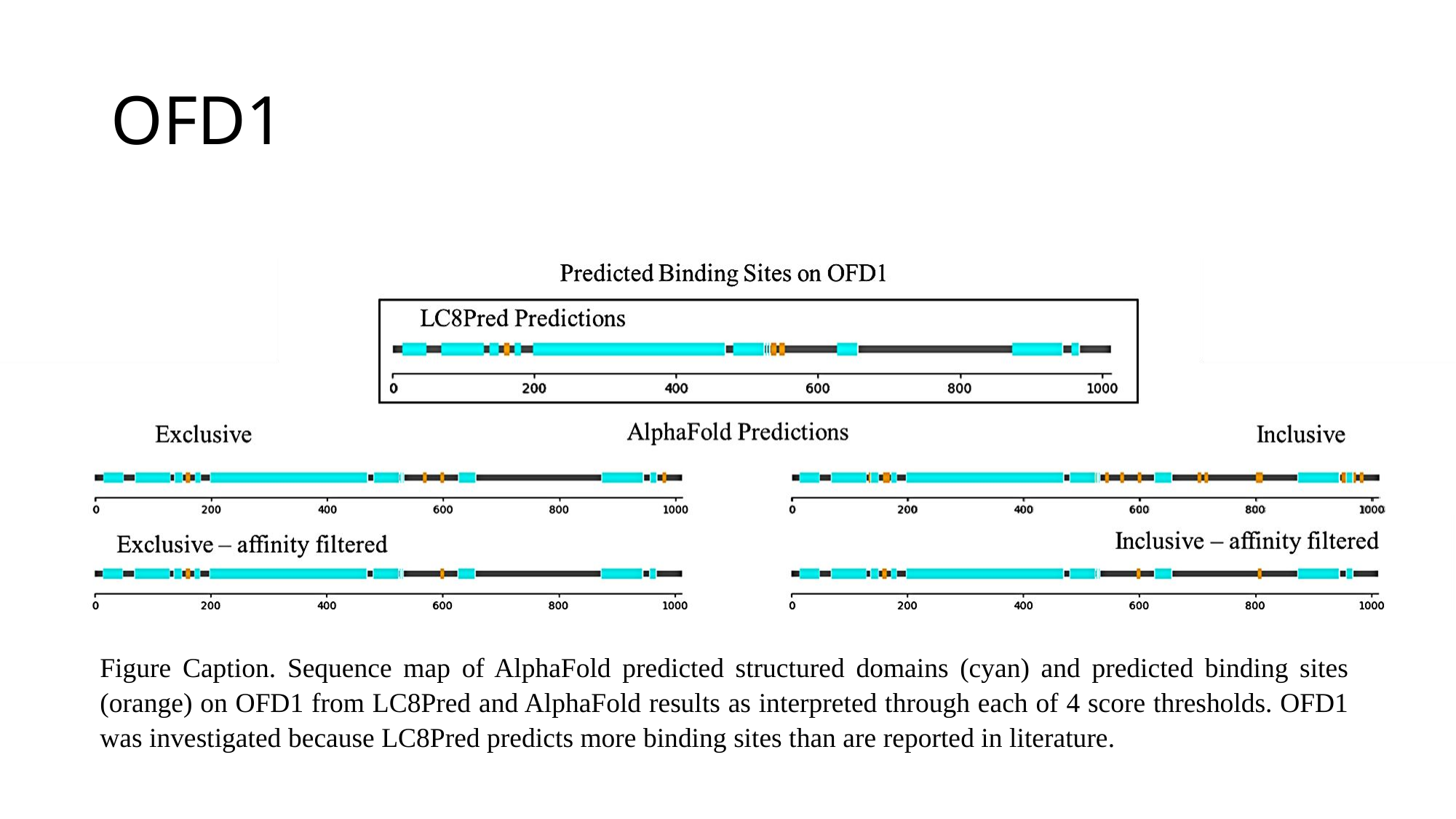

# OFD1
Figure Caption. Sequence map of AlphaFold predicted structured domains (cyan) and predicted binding sites (orange) on OFD1 from LC8Pred and AlphaFold results as interpreted through each of 4 score thresholds. OFD1 was investigated because LC8Pred predicts more binding sites than are reported in literature.

## Slide 15
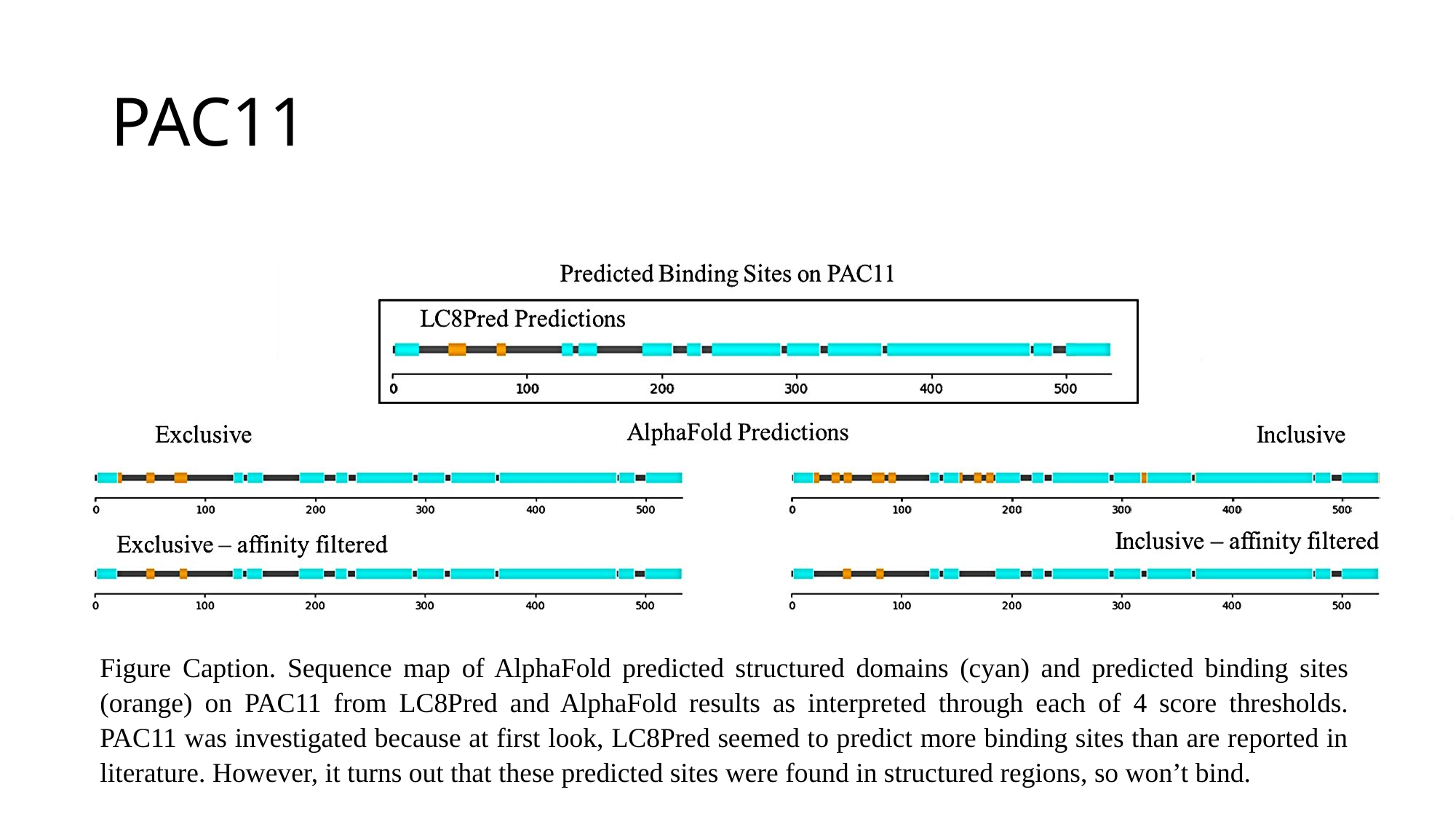

# PAC11
Figure Caption. Sequence map of AlphaFold predicted structured domains (cyan) and predicted binding sites (orange) on PAC11 from LC8Pred and AlphaFold results as interpreted through each of 4 score thresholds. PAC11 was investigated because at first look, LC8Pred seemed to predict more binding sites than are reported in literature. However, it turns out that these predicted sites were found in structured regions, so won’t bind.

## Slide 16
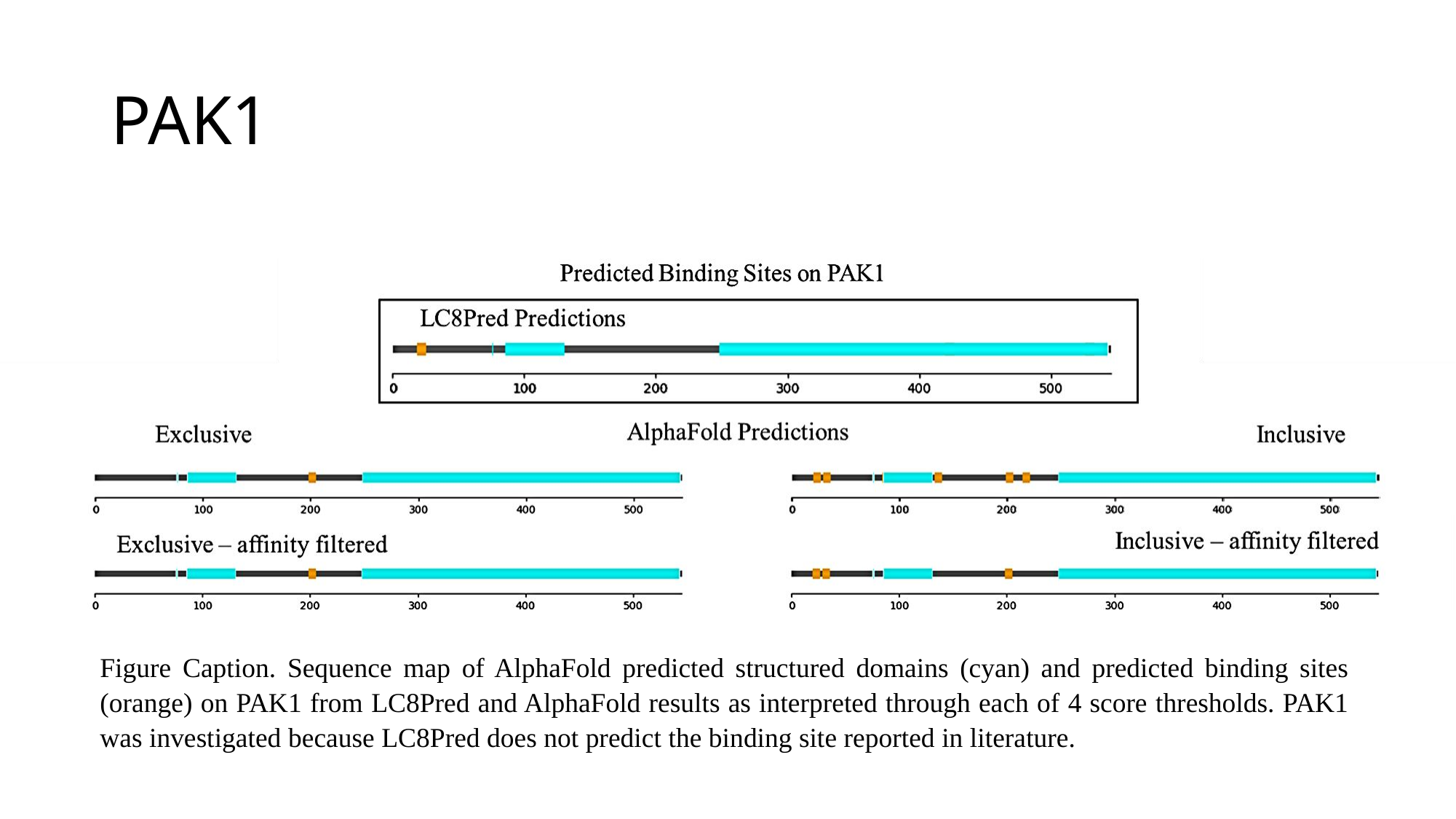

# PAK1
Figure Caption. Sequence map of AlphaFold predicted structured domains (cyan) and predicted binding sites (orange) on PAK1 from LC8Pred and AlphaFold results as interpreted through each of 4 score thresholds. PAK1 was investigated because LC8Pred does not predict the binding site reported in literature.

## Slide 17
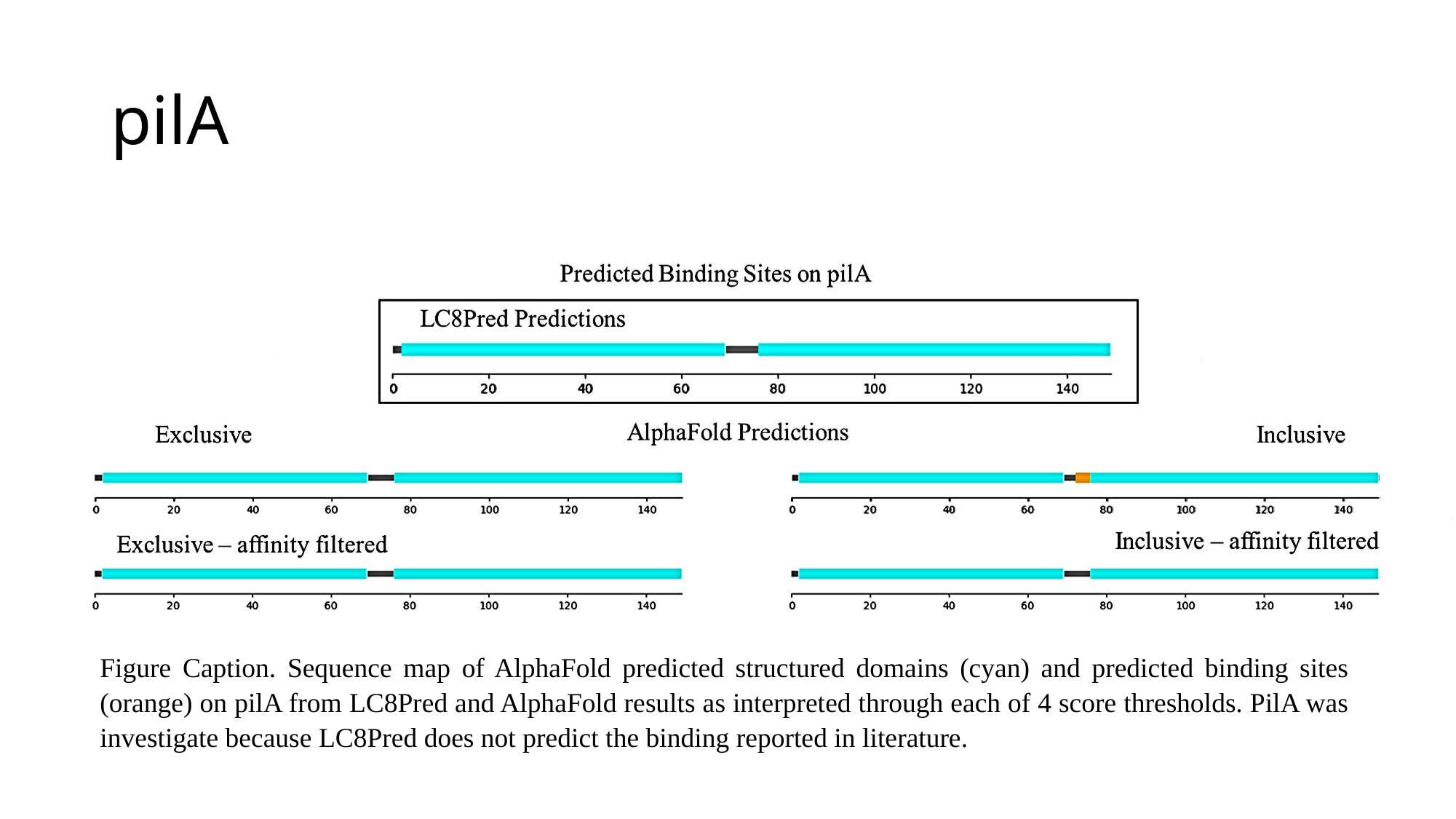

# pilA
Figure Caption. Sequence map of AlphaFold predicted structured domains (cyan) and predicted binding sites (orange) on pilA from LC8Pred and AlphaFold results as interpreted through each of 4 score thresholds. PilA was investigate because LC8Pred does not predict the binding reported in literature.

## Slide 18
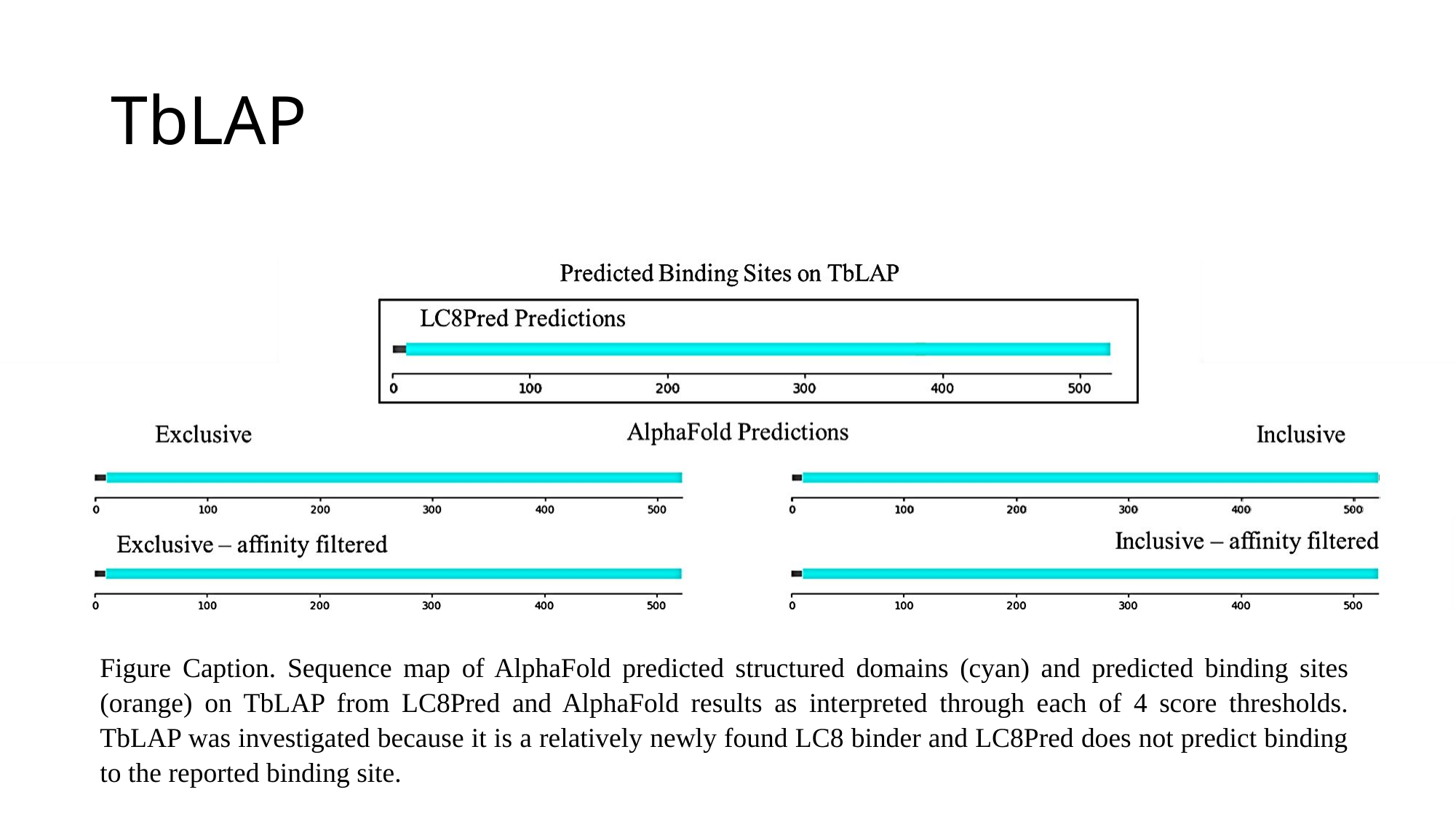

# TbLAP
Figure Caption. Sequence map of AlphaFold predicted structured domains (cyan) and predicted binding sites (orange) on TbLAP from LC8Pred and AlphaFold results as interpreted through each of 4 score thresholds. TbLAP was investigated because it is a relatively newly found LC8 binder and LC8Pred does not predict binding to the reported binding site.

## Slide 19
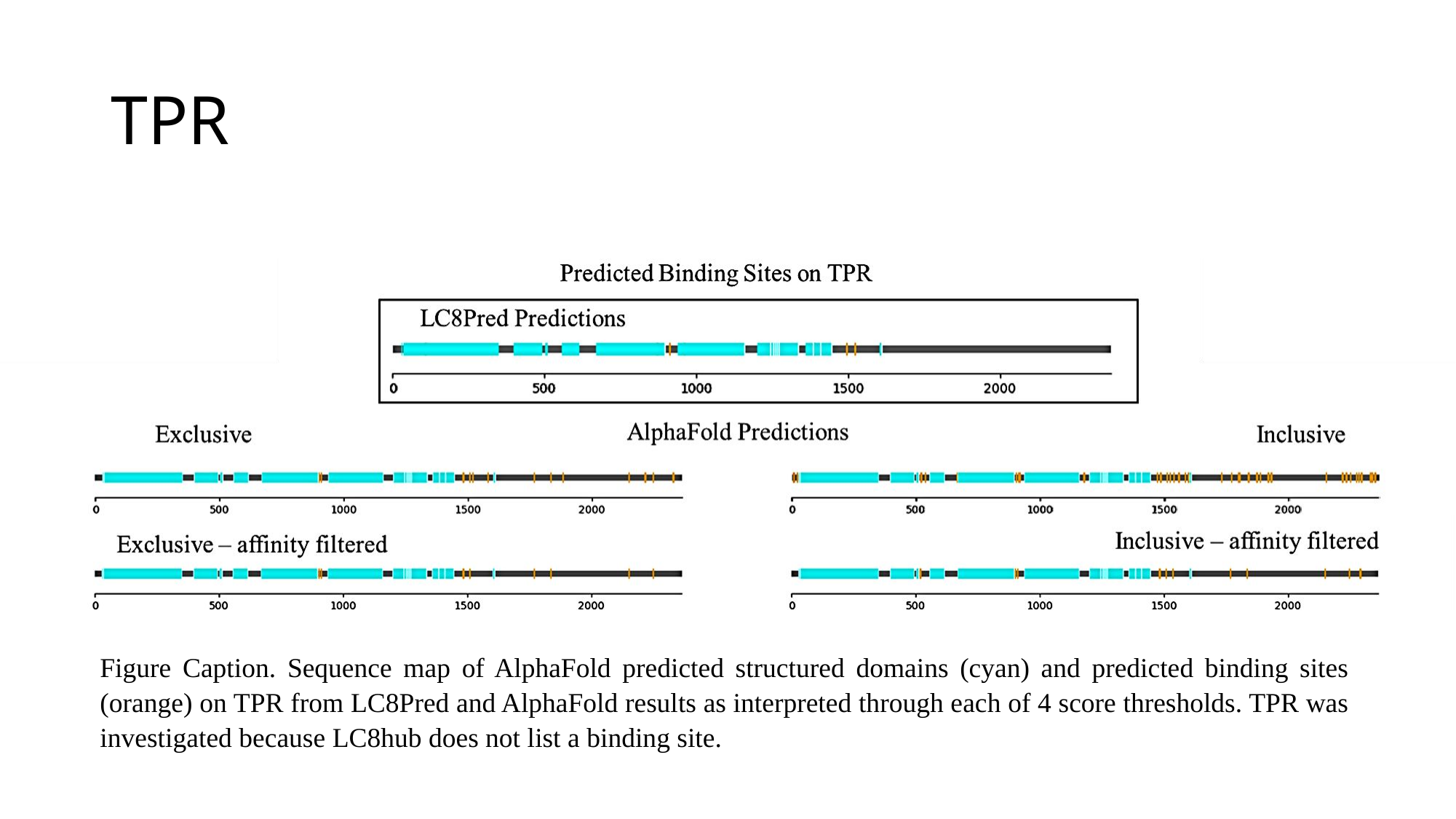

# TPR
Figure Caption. Sequence map of AlphaFold predicted structured domains (cyan) and predicted binding sites (orange) on TPR from LC8Pred and AlphaFold results as interpreted through each of 4 score thresholds. TPR was investigated because LC8hub does not list a binding site.

## Slide 20
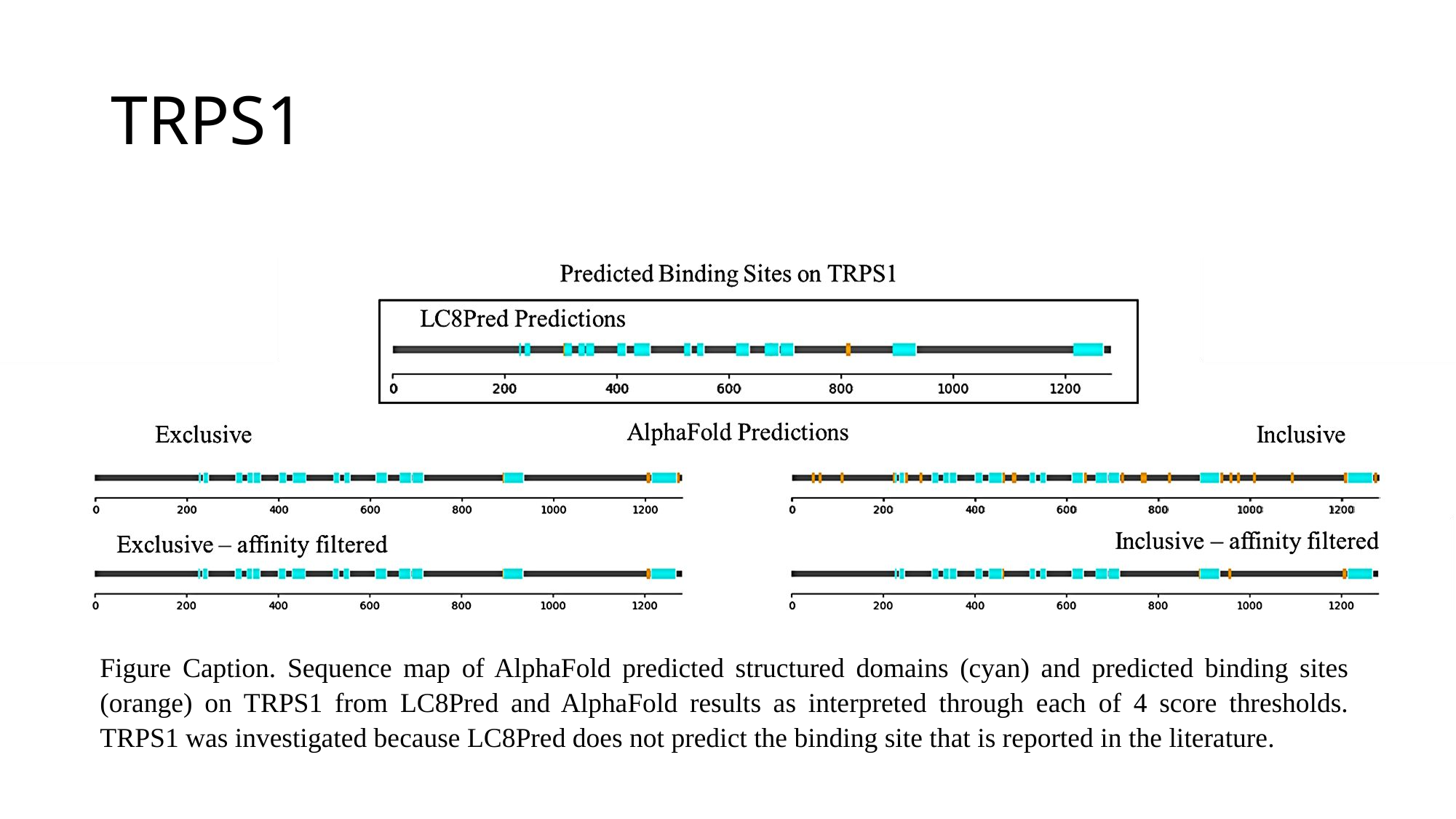

# TRPS1
Figure Caption. Sequence map of AlphaFold predicted structured domains (cyan) and predicted binding sites (orange) on TRPS1 from LC8Pred and AlphaFold results as interpreted through each of 4 score thresholds. TRPS1 was investigated because LC8Pred does not predict the binding site that is reported in the literature.

## Slide 21
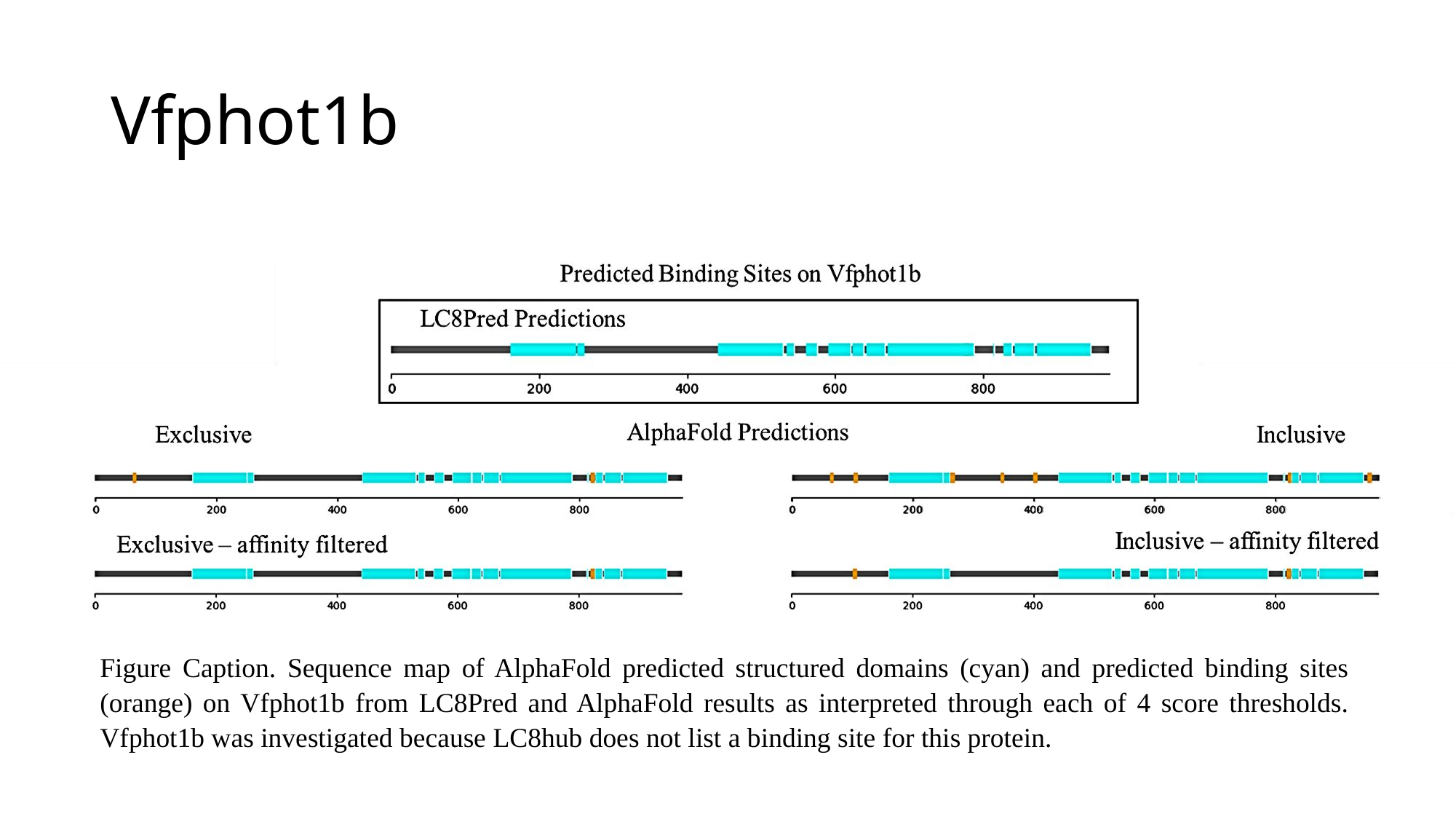

# Vfphot1b
Figure Caption. Sequence map of AlphaFold predicted structured domains (cyan) and predicted binding sites (orange) on Vfphot1b from LC8Pred and AlphaFold results as interpreted through each of 4 score thresholds. Vfphot1b was investigated because LC8hub does not list a binding site for this protein.

## Slide 22
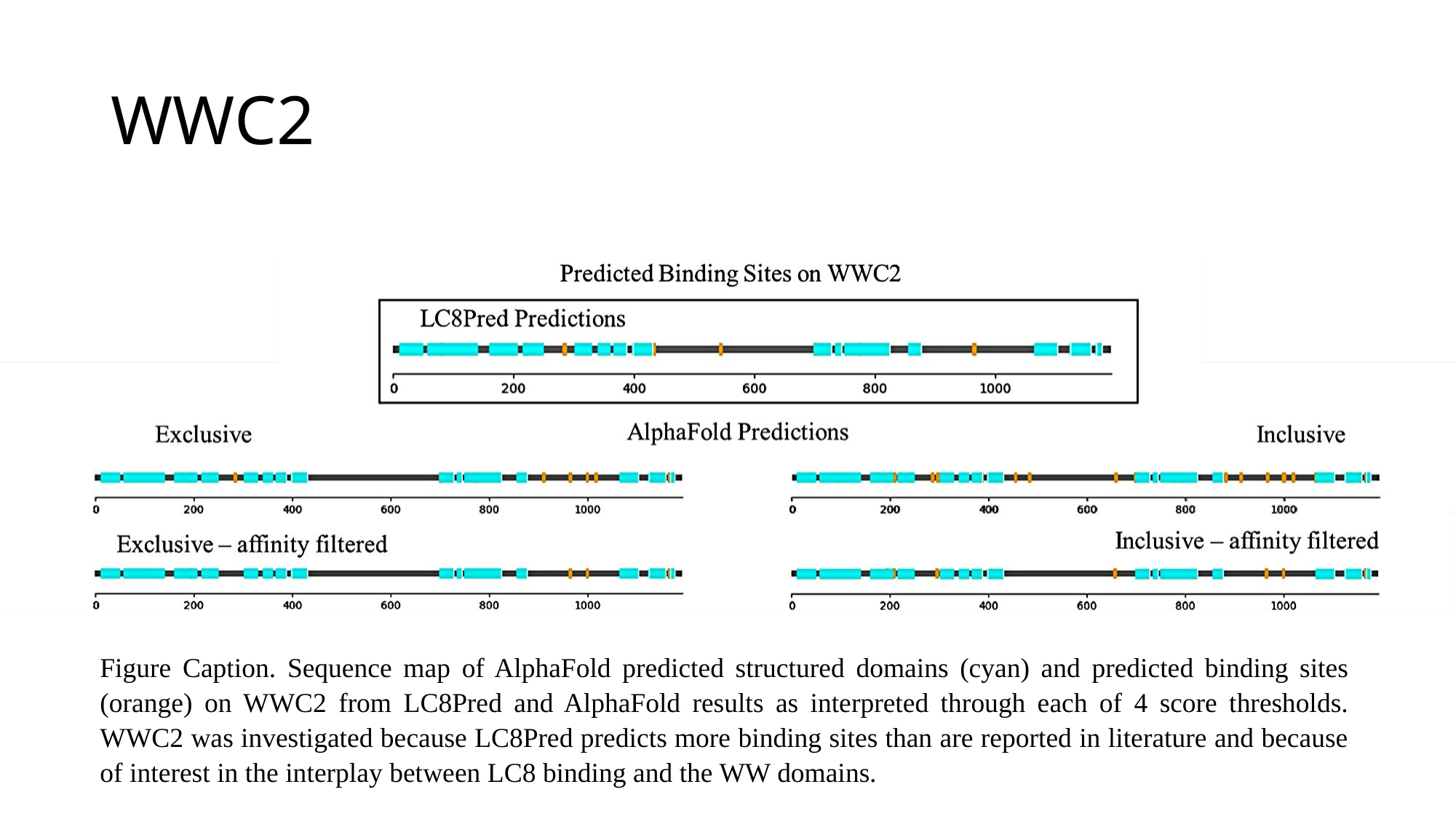

# WWC2
Figure Caption. Sequence map of AlphaFold predicted structured domains (cyan) and predicted binding sites (orange) on WWC2 from LC8Pred and AlphaFold results as interpreted through each of 4 score thresholds. WWC2 was investigated because LC8Pred predicts more binding sites than are reported in literature and because of interest in the interplay between LC8 binding and the WW domains.

## Slide 23
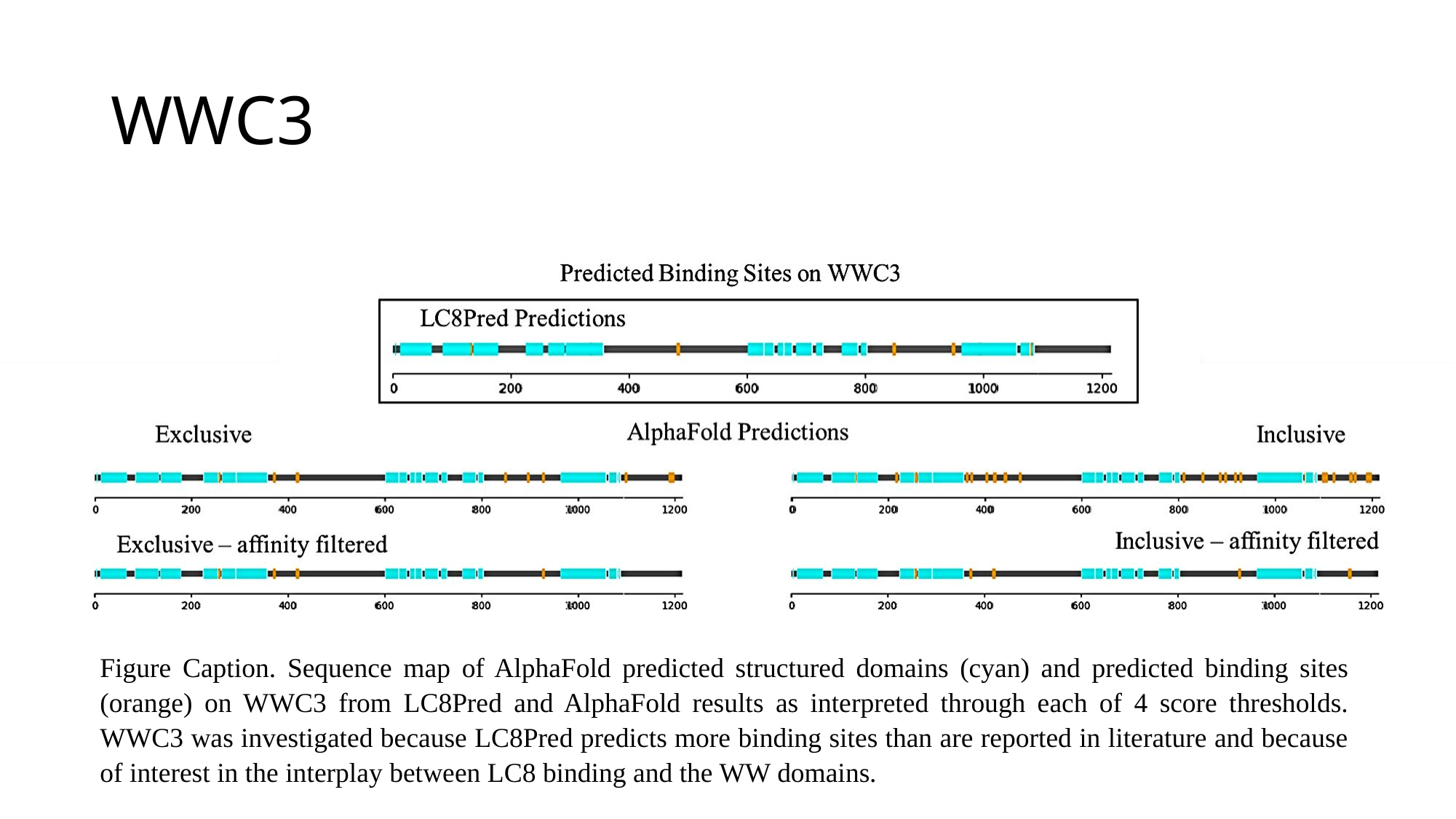

# WWC3
Figure Caption. Sequence map of AlphaFold predicted structured domains (cyan) and predicted binding sites (orange) on WWC3 from LC8Pred and AlphaFold results as interpreted through each of 4 score thresholds. WWC3 was investigated because LC8Pred predicts more binding sites than are reported in literature and because of interest in the interplay between LC8 binding and the WW domains.

## Slide 24
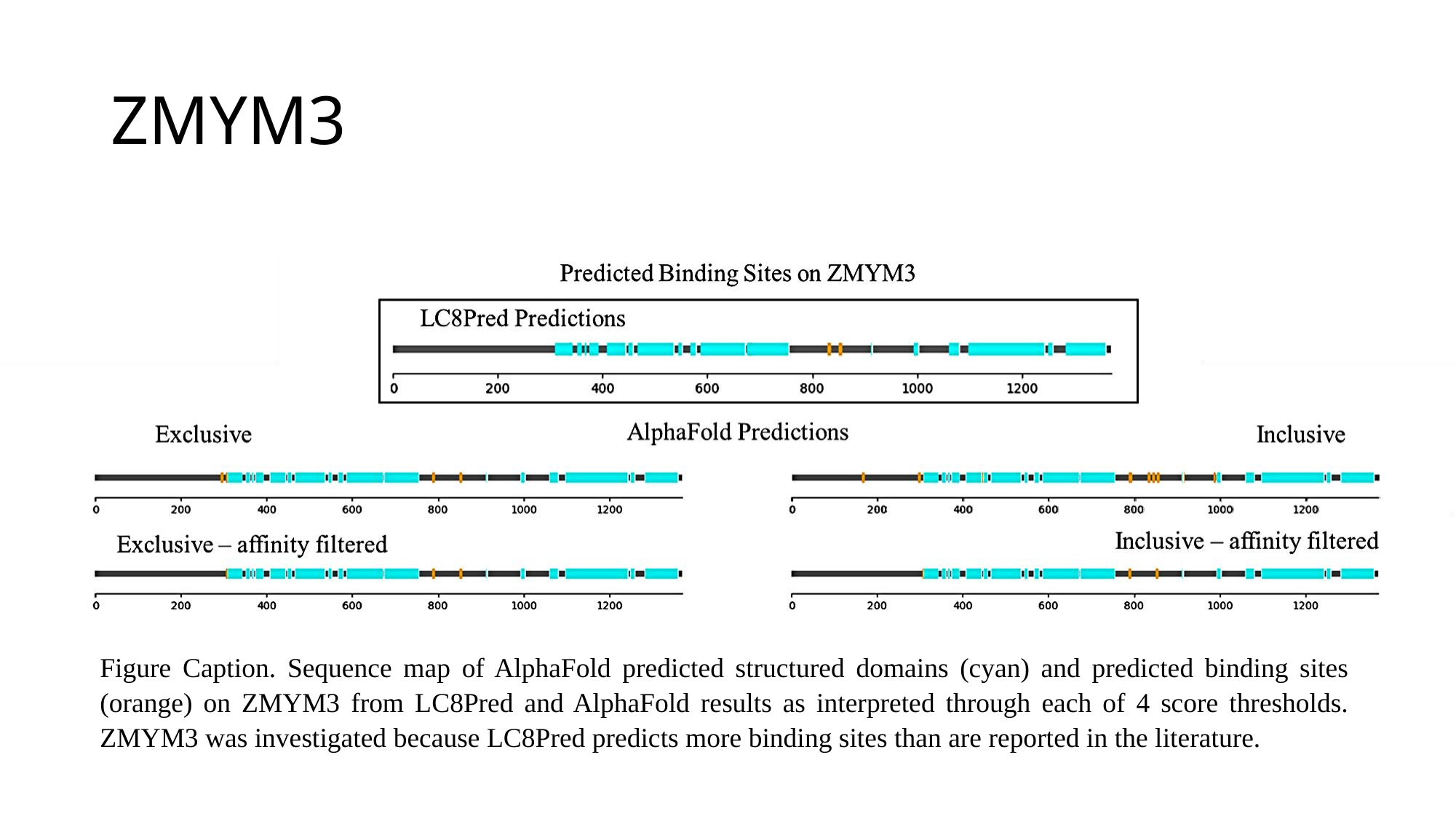

# ZMYM3
Figure Caption. Sequence map of AlphaFold predicted structured domains (cyan) and predicted binding sites (orange) on ZMYM3 from LC8Pred and AlphaFold results as interpreted through each of 4 score thresholds. ZMYM3 was investigated because LC8Pred predicts more binding sites than are reported in the literature.
