# Supplementary figures and images for: Successful prediction of LC8 binding to intrinsically disordered proteins sheds light on AlphaFold’s black box
Source: Front Mol Biosci. 2025 Apr 23;12:1531793. doi: 10.3389/fmolb.2025.1531793 (PMC12057147; doi:10.3389/fmolb.2025.1531793)

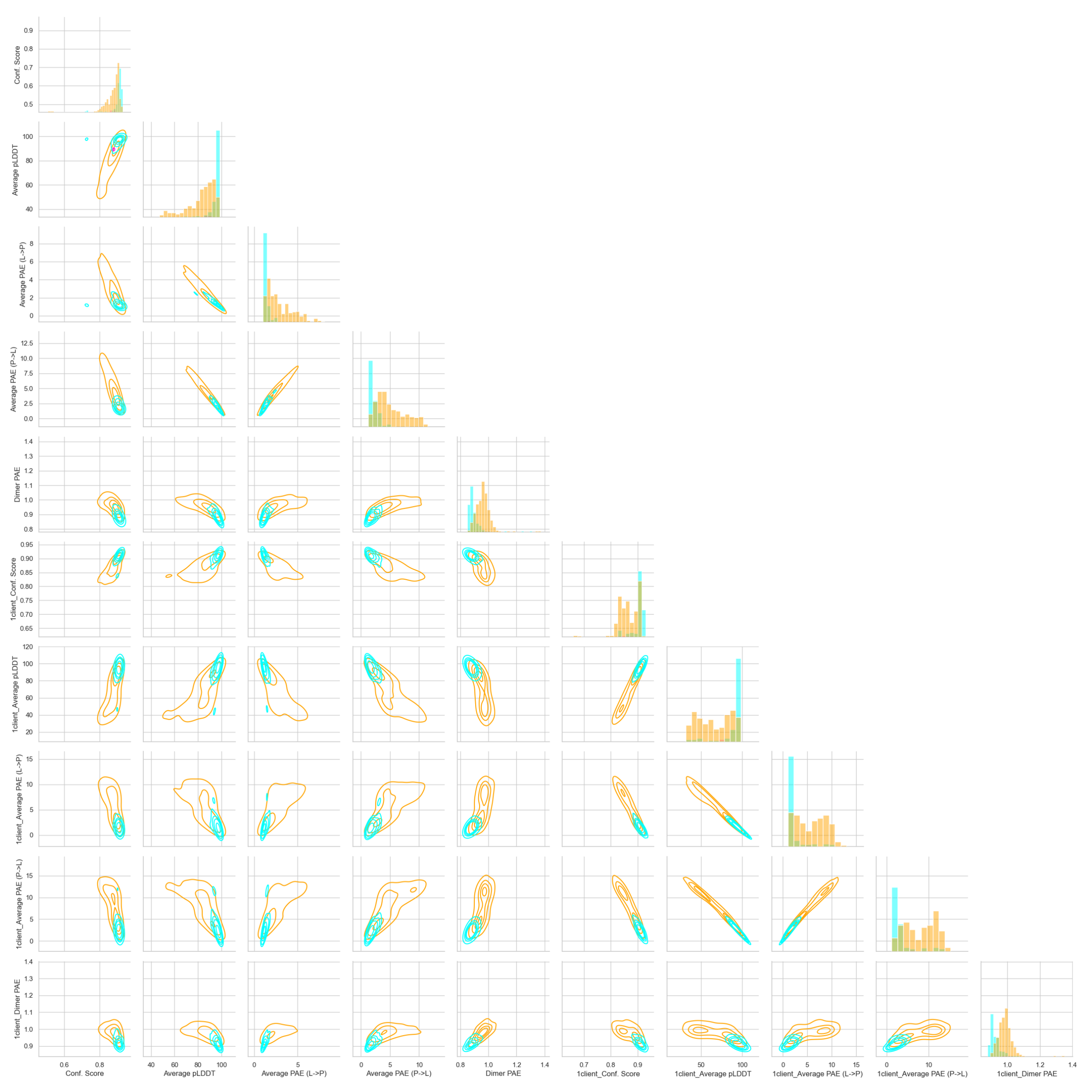

Supplement: Supplementary file 16 [file Image2.png]

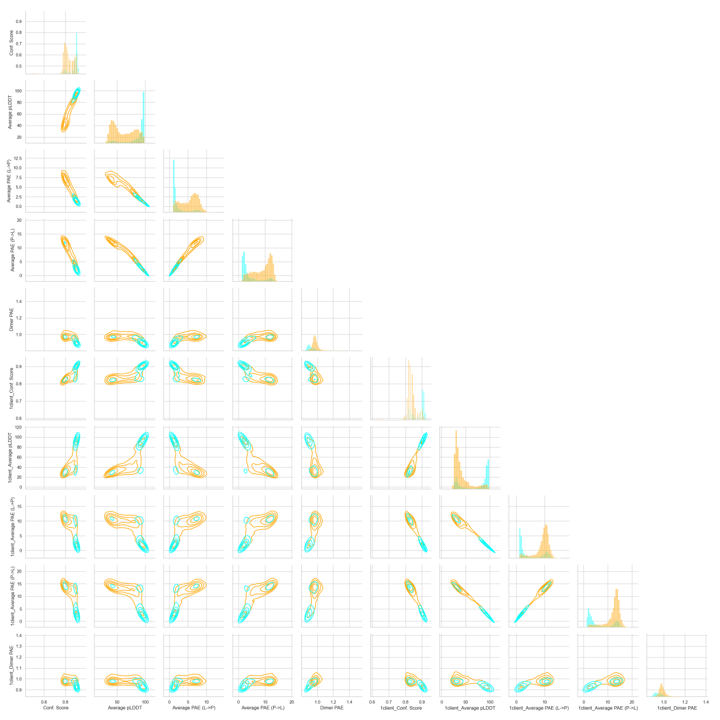

Supplement: Supplementary file 20 [file Image1.png]
