# Supplementary material for: Successful prediction of LC8 binding to intrinsically disordered proteins sheds light on AlphaFold’s black box
Source: Front Mol Biosci. 2025 Apr 23;12:1531793. doi: 10.3389/fmolb.2025.1531793 (PMC12057147; doi:10.3389/fmolb.2025.1531793)
Supplement: Supplementary file 18 [file Presentation3.pptx]

## Slide 1
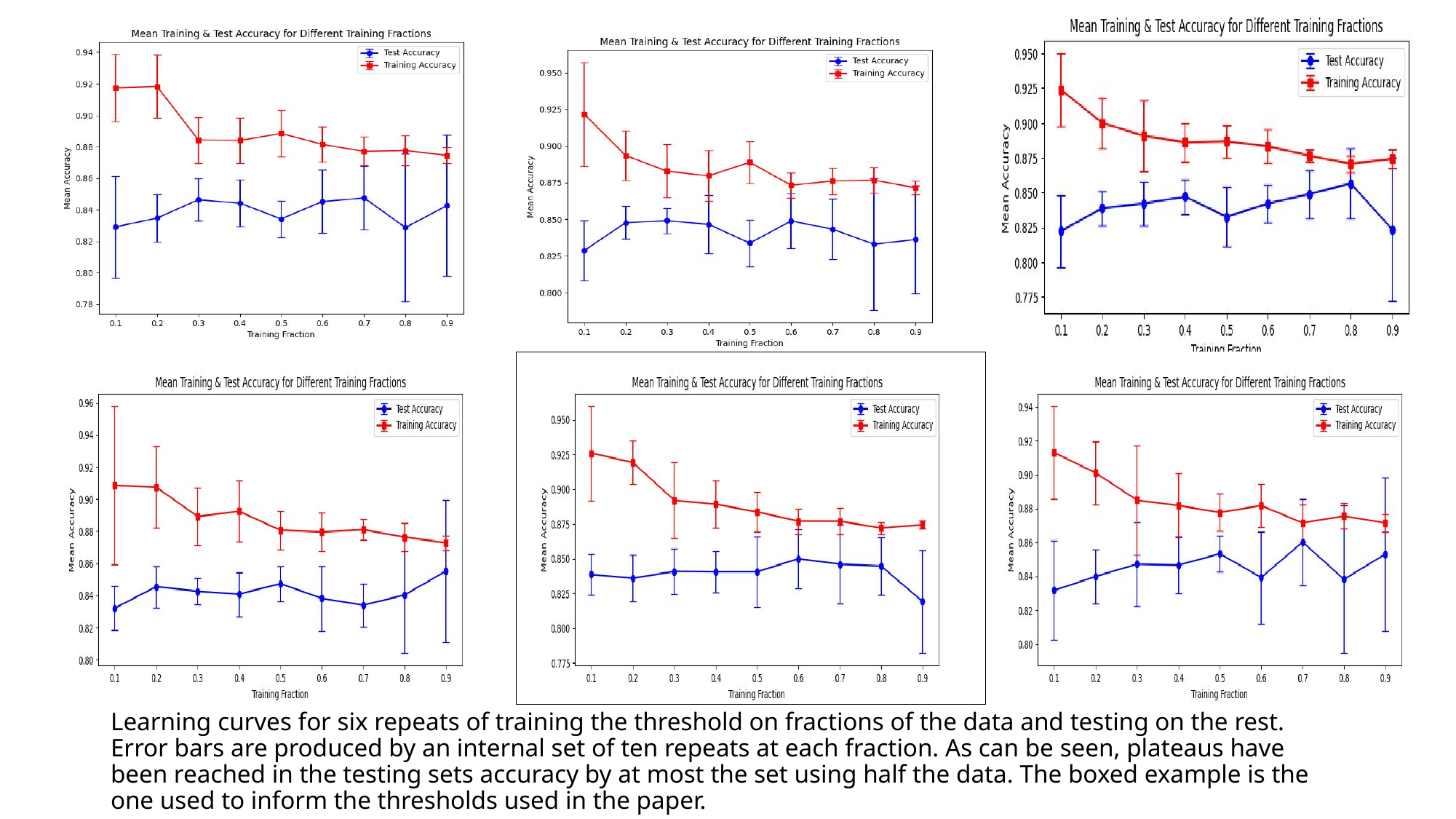

Learning curves for six repeats of training the threshold on fractions of the data and testing on the rest. Error bars are produced by an internal set of ten repeats at each fraction. As can be seen, plateaus have been reached in the testing sets accuracy by at most the set using half the data. The boxed example is the one used to inform the thresholds used in the paper.

## Slide 2
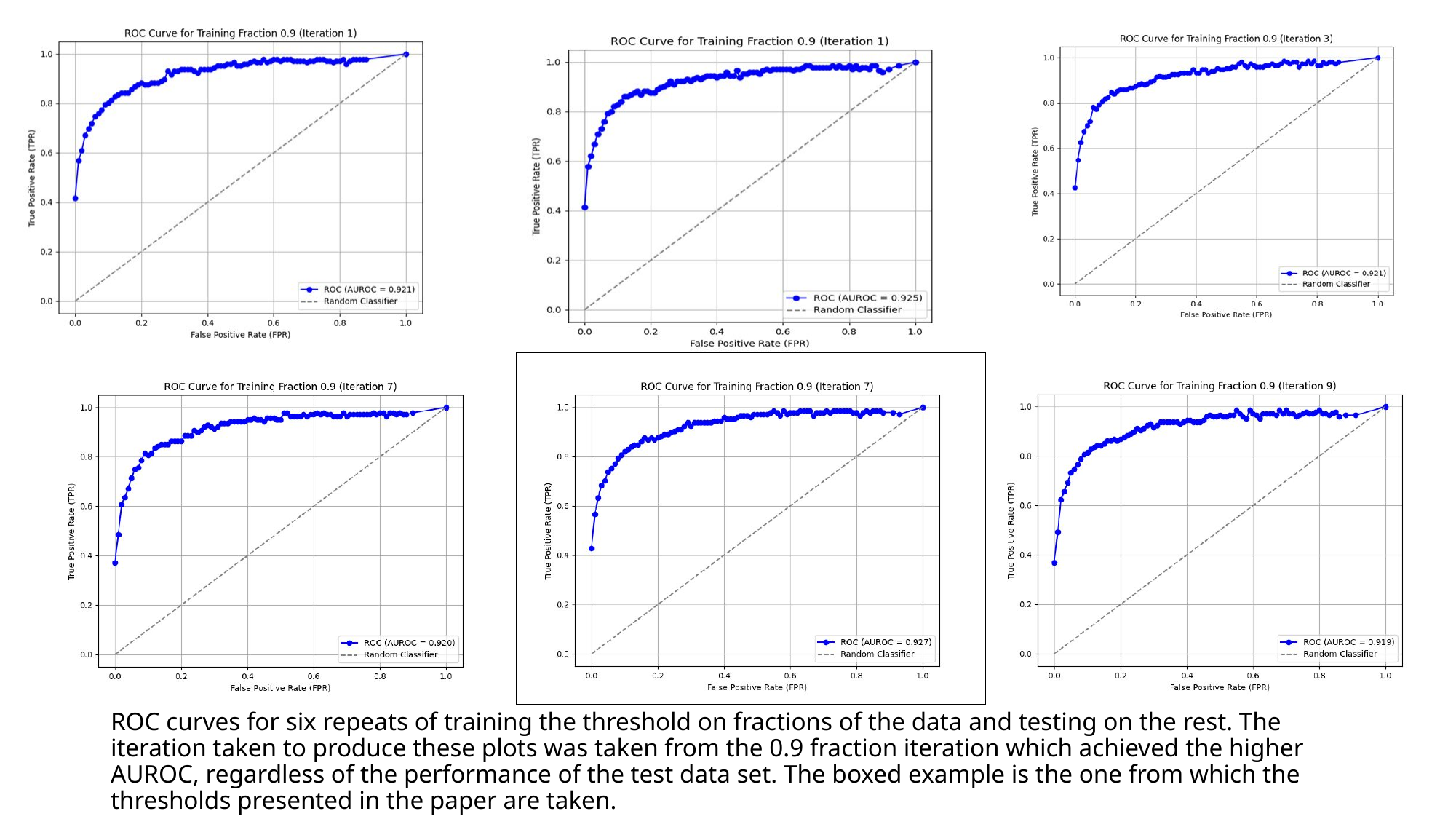

ROC curves for six repeats of training the threshold on fractions of the data and testing on the rest. The iteration taken to produce these plots was taken from the 0.9 fraction iteration which achieved the higher AUROC, regardless of the performance of the test data set. The boxed example is the one from which the thresholds presented in the paper are taken.

## Slide 3
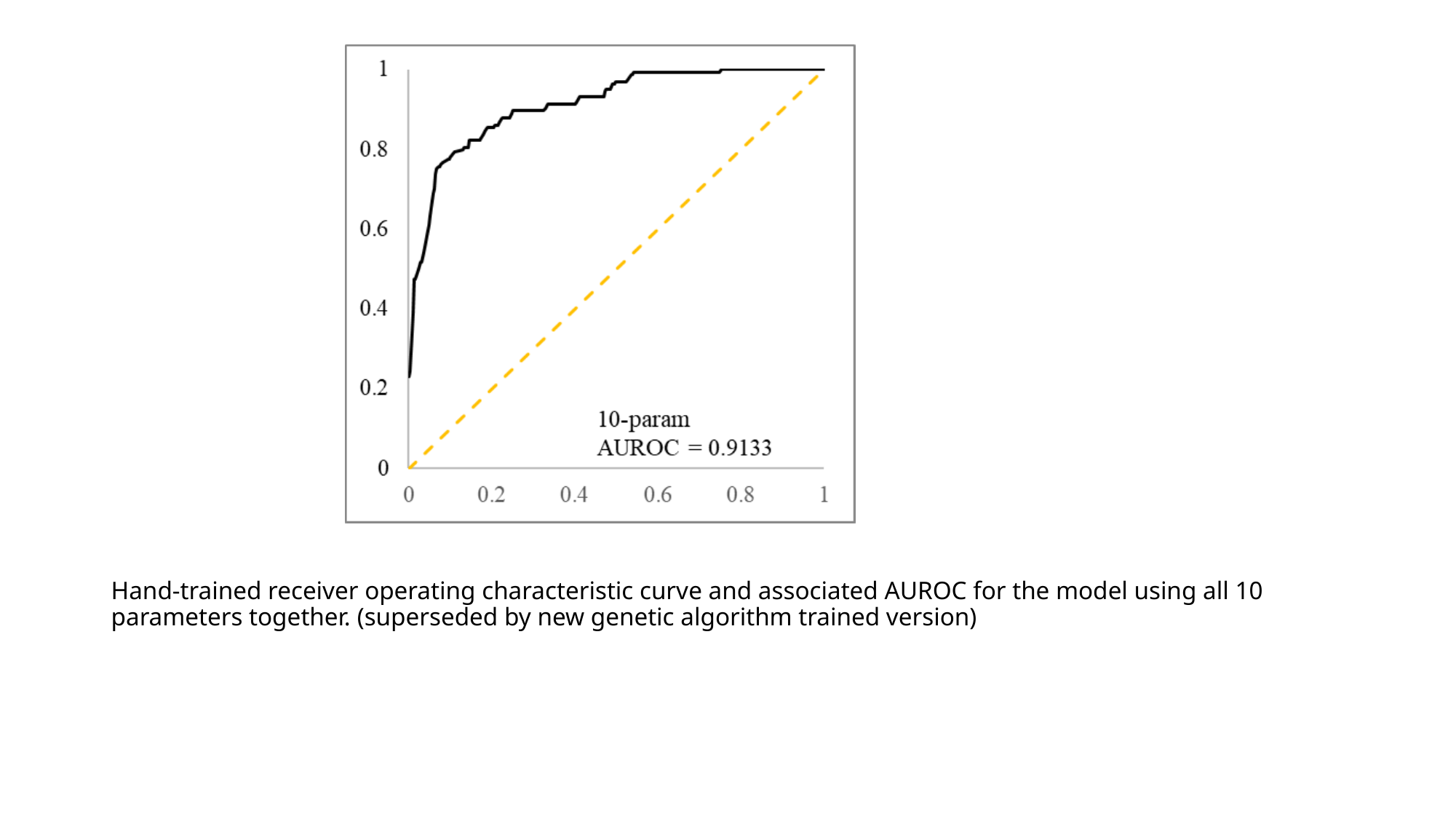

Hand-trained receiver operating characteristic curve and associated AUROC for the model using all 10 parameters together. (superseded by new genetic algorithm trained version)

## Slide 4
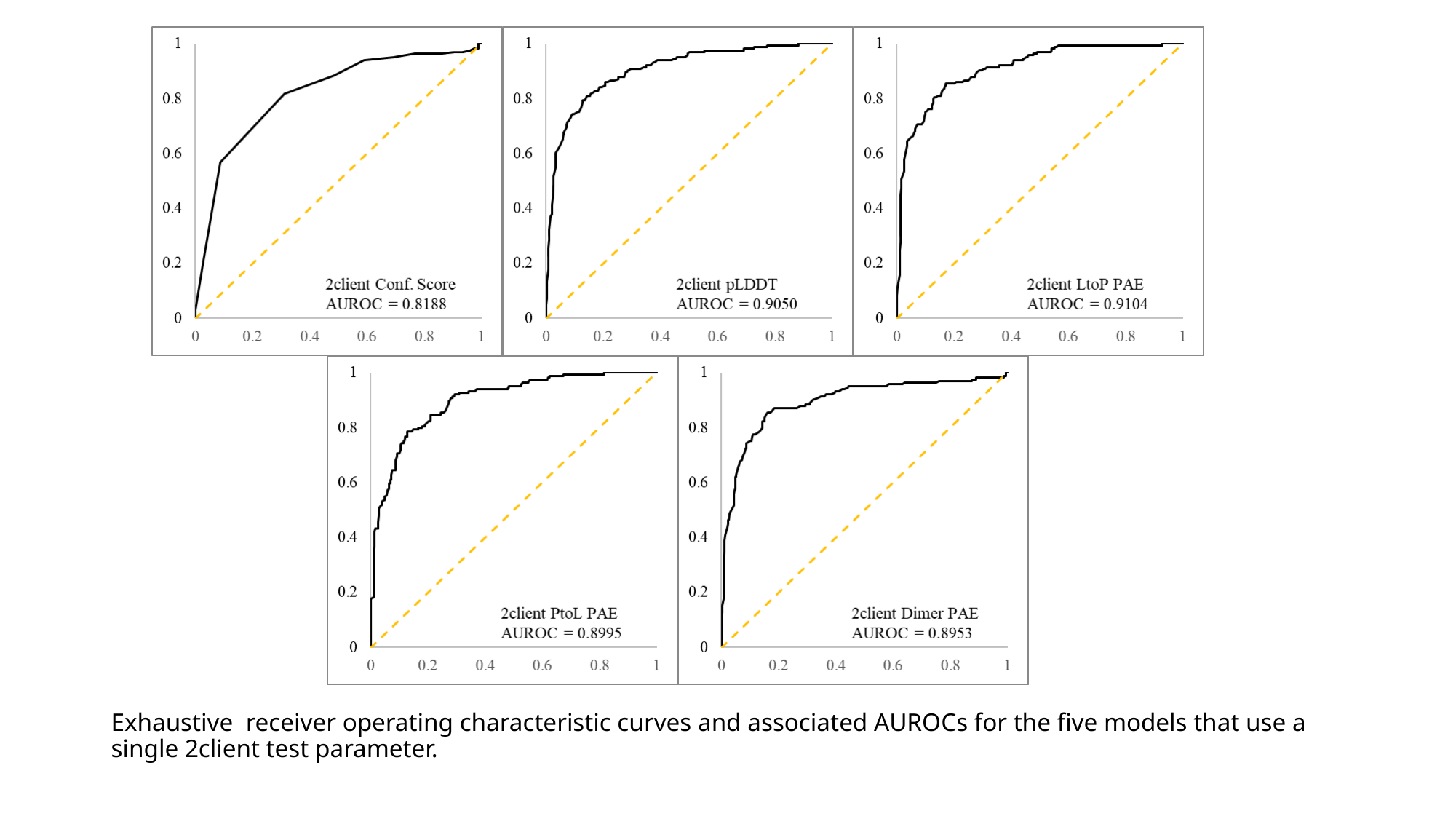

Exhaustive receiver operating characteristic curves and associated AUROCs for the five models that use a single 2client test parameter.

## Slide 5
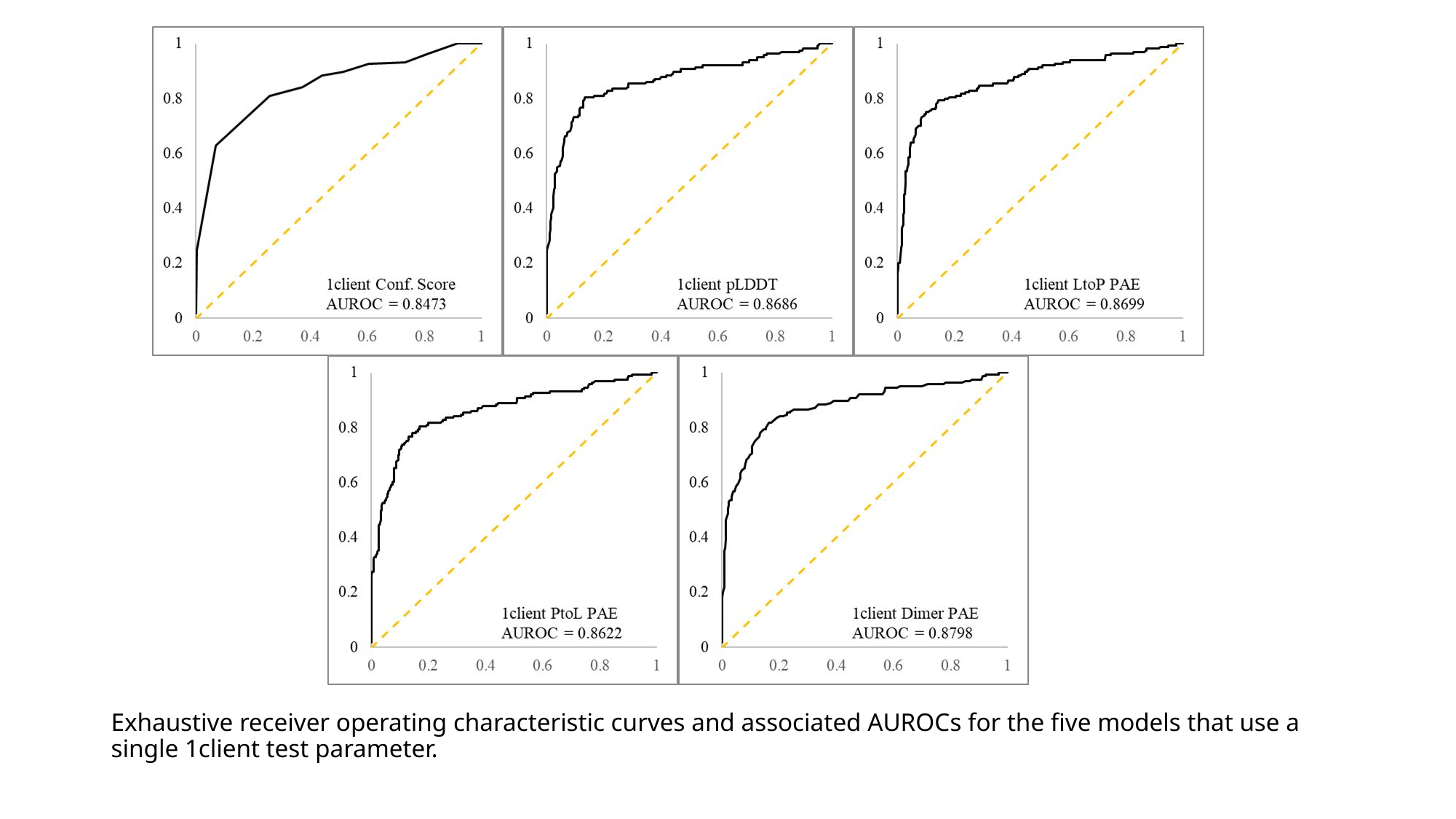

Exhaustive receiver operating characteristic curves and associated AUROCs for the five models that use a single 1client test parameter.
