# Supplementary material for: Successful prediction of LC8 binding to intrinsically disordered proteins sheds light on AlphaFold’s black box
Source: Front Mol Biosci. 2025 Apr 23;12:1531793. doi: 10.3389/fmolb.2025.1531793 (PMC12057147; doi:10.3389/fmolb.2025.1531793)
Supplement: Supplementary file 19 [file Presentation2.pptx]

## Slide 1
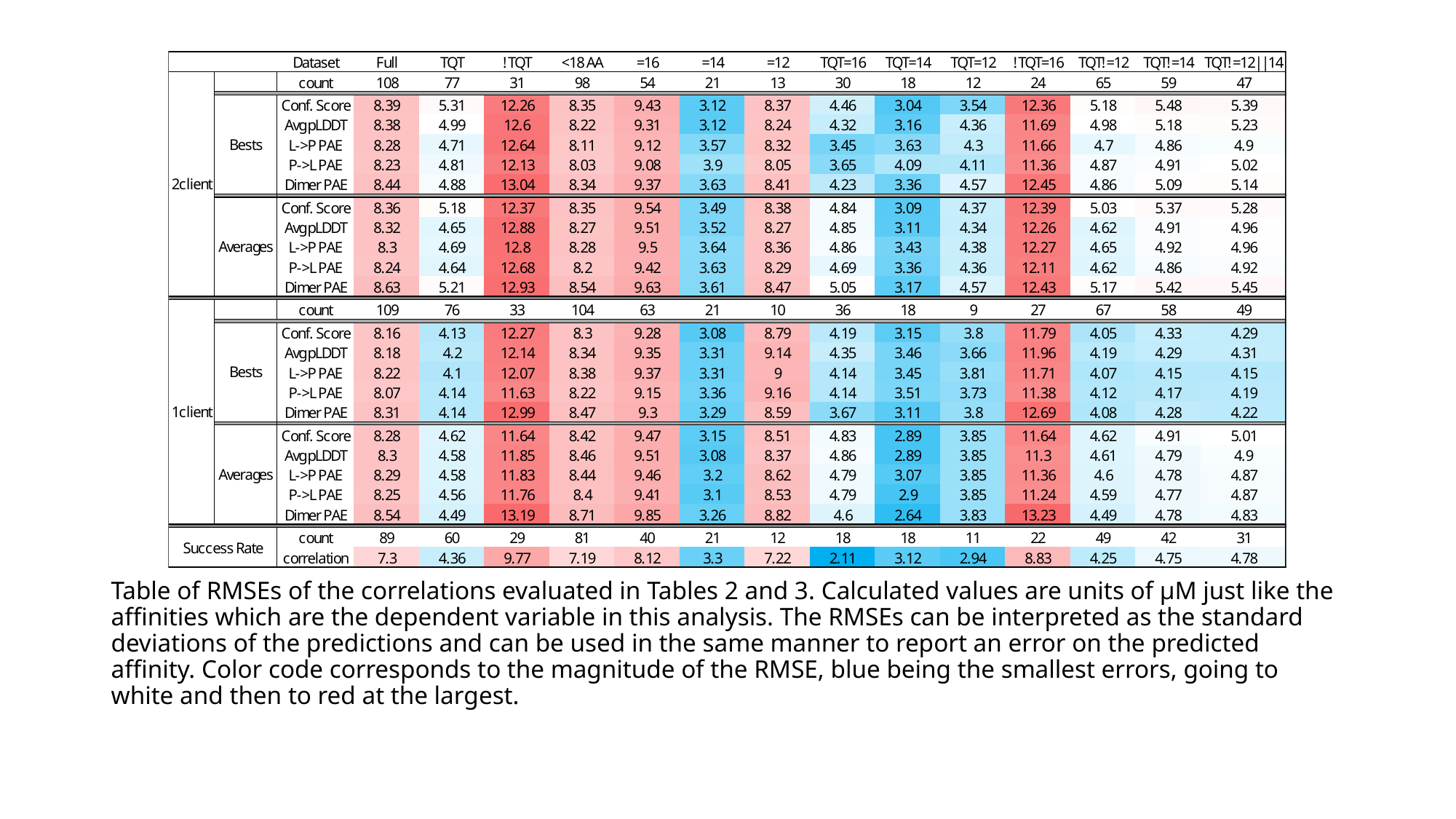

Table of RMSEs of the correlations evaluated in Tables 2 and 3. Calculated values are units of µM just like the affinities which are the dependent variable in this analysis. The RMSEs can be interpreted as the standard deviations of the predictions and can be used in the same manner to report an error on the predicted affinity. Color code corresponds to the magnitude of the RMSE, blue being the smallest errors, going to white and then to red at the largest.

## Slide 2
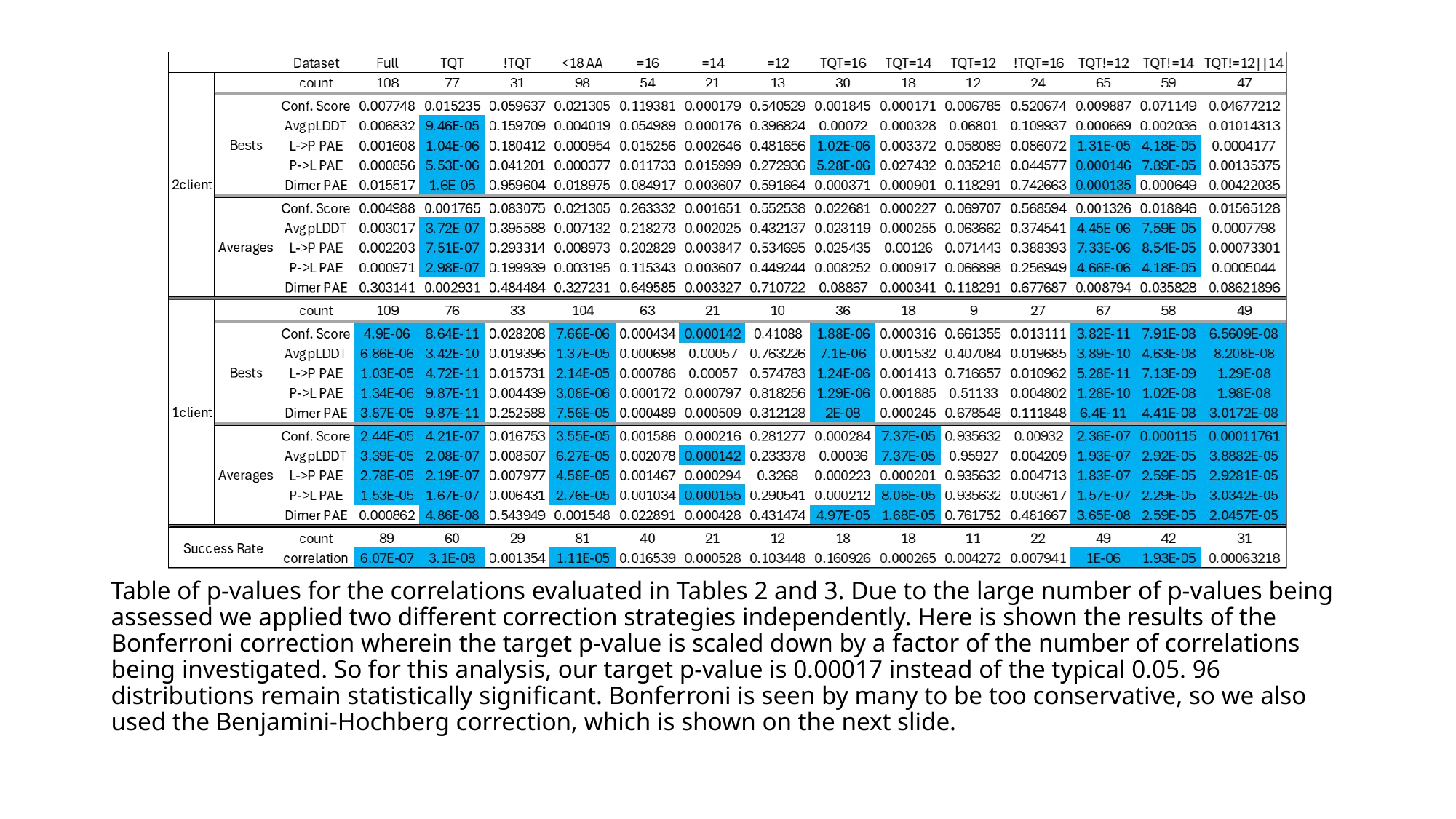

Table of p-values for the correlations evaluated in Tables 2 and 3. Due to the large number of p-values being assessed we applied two different correction strategies independently. Here is shown the results of the Bonferroni correction wherein the target p-value is scaled down by a factor of the number of correlations being investigated. So for this analysis, our target p-value is 0.00017 instead of the typical 0.05. 96 distributions remain statistically significant. Bonferroni is seen by many to be too conservative, so we also used the Benjamini-Hochberg correction, which is shown on the next slide.

## Slide 3
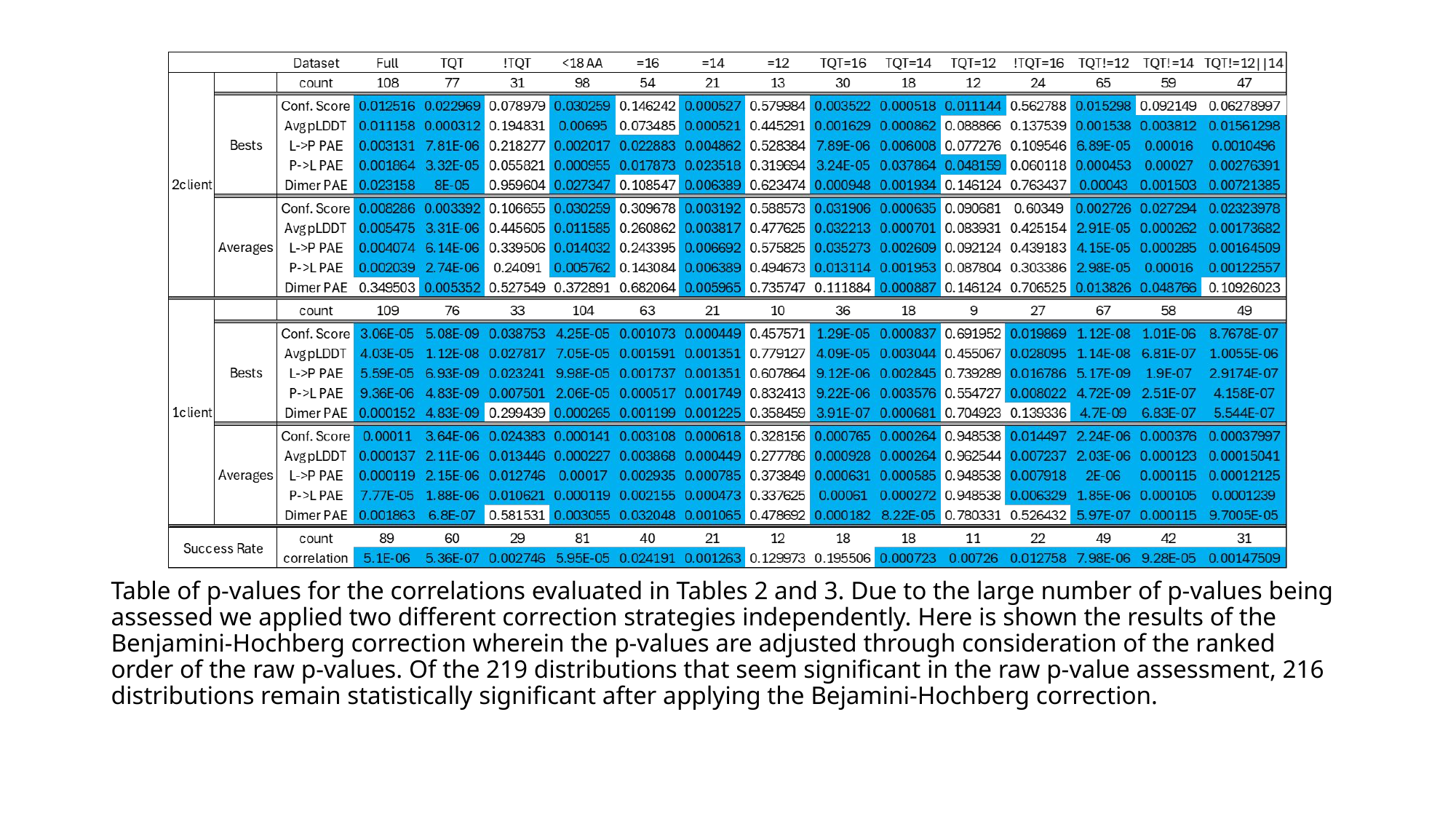

Table of p-values for the correlations evaluated in Tables 2 and 3. Due to the large number of p-values being assessed we applied two different correction strategies independently. Here is shown the results of the Benjamini-Hochberg correction wherein the p-values are adjusted through consideration of the ranked order of the raw p-values. Of the 219 distributions that seem significant in the raw p-value assessment, 216 distributions remain statistically significant after applying the Bejamini-Hochberg correction.
